# Facile N═N Bond Cleavage of *Cis*‐Azobenzene with Bis‐silylenes

**DOI:** 10.1002/anie.202507560

**Published:** 2025-05-10

**Authors:** Yun Xiong, Shenglai Yao, Matthias Driess

**Affiliations:** ^1^ Department of Chemistry: Metalorganics and Inorganic Materials Technische Universität Berlin Strasse des 17. Juni 115, Sekr. C2 10623 Berlin Germany

**Keywords:** Azobenzene, Cooperativity, Cycloaddition, Dinitrogen activation, Silylenes

## Abstract

The very different features of cooperative disilicon(II)‐mediated N═N bond activation of *trans*‐ vs. *cis*‐azobenzene are reported, employing two bis‐silylenes with distinct intramolecular Si···Si distances, PhN(LSi:)₂ **1** (L = PhC(*^t^* BuN)₂, Si···Si: 2.9 Å) and XT(LSi:)₂ **2** (XT = 9,9‐dimethyl‐xanthene‐4,5‐diyl, Si···Si: 4.3 Å). While *trans*‐azobenzene reacts with both bis‐silylenes to form C─H and N═N π bond activation products, the *cis*‐isomer undergoes only N═N bond scission. Thus, the reaction of **1** with *cis*‐azobenzene at room temperature affords the unprecedented N═N bond cleavage product **4**, featuring a bis‐silaimine with terminal and bridging Si═N moieties. In contrast, the reaction of **2** with *cis*‐azobenzene at –30 °C in THF allows for the isolation of the [1+2] cycloaddition intermediate **6**, containing a three‐membered SiN₂ ring (siladiazirane), which rearranges to the N═N bond cleavage product **8** at room temperature. Compound **6** reacts with one additional equivalent of *cis*‐azobenzene to form bis‐silaazirane **7** with two SiN₂ rings. Density functional theory (DFT) calculations support stepwise Si(II)···Si(II) cooperative activation mechanisms and provide insights into the role of bis‐silylenes for selective N═N cleavage reactions.

## Graphical abstract

The unexpectedly easy N═N bond scission of *cis*‐azobenzene using two bis‐silylenes with different spacers and distinct Si^II^···Si^II^ distances is reported. While both bis‐silylenes enable C─H and N═N π‐bond activation of *trans*‐azobenzene, the cooperative disilicon(II)‐mediated activation of *cis*‐azobenzene leads to bis‐silamines (Si═N) and related novel products. Density functional theory (DFT) calculations shed light on the mechanism and nature of the intermediates of the N═N cleavage products.

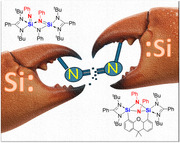

## Introduction

Featuring an N═N π‐bond, azobenzene is an invaluable starting material for the synthesis of organonitrogen compounds with diverse applications. It has also attracted considerable attention due to its intriguing photoisomerization properties^[^ ^1^, ^2^ ^]^ and its relevance in fields such as molecular devices^[^ ^3^ ^]^ and pharmacology.^[^ ^4^ ^]^ The cleavage of the N═N bond in RN═NR compounds is not only essential for developing novel transformations that incorporate azo compounds as a source for [R─N] moieties in synthetic chemistry, but also vital as model compounds for understanding the mechanisms of dinitrogen fixation, scission, and functionalization.^[^ ^5^, ^6^, ^7^ ^]^ To date, most studies on N═N bond cleavage in azo compounds involve transition metals. Low‐valent transition‐metal complexes can mediate N═N bond scission in azo species to afford mono‐ or dinuclear metal imido complexes.^[^ ^8^, ^9^, ^10^, ^11^, ^12^, ^13^, ^14^, ^15^ ^]^ At the same time, many transition metals also facilitate *ortho*‐CH bond activation of the phenyl groups using *trans*‐azobenzene.^[^ ^16^, ^17^ ^]^

**Scheme 1:**
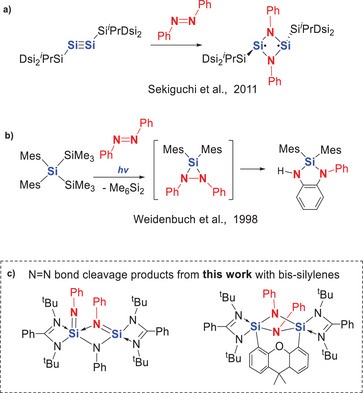
Known examples of N═N cleavage mediated by: a) a Si≡Si disilyne species (Dsi = CH(SiMe_3_)_2_); b) in situ generated Mes_2_Si: silylene (Mes = 2,4,6‐Me_3_C_6_H_2_) c) The novel N═N bond cleavage products from this work are mediated by two bis‐silylenes.

In the same vein, N═N bond activation of azo compounds can also be accomplished with subvalent main‐group elements. For example, dinuclear low‐valent main‐group complexes such as [(PhC(N*^t^* Bu)_2_)_2_Ge_2_],^[^ ^18^ ^]^ ArGaGaAr (Ar = C_6_H_3_‐2,6‐(C_6_H_3_‐2,6‐*^i^* Pr_2_)_2_),^[^ ^19^ ^]^ [RMgMgR] (R = [(Ar´NCMe)_2_CH], Ar´ = C_6_H_3_‐2,6‐*^i^* Pr_2_),^[^ ^20^ ^]^ and [ArEEAr]; E = Ge, Sn)^[^ ^21^ ^]^ have demonstrated both their suitability to bind the N═N moiety of *trans*‐azobenzene and to activate an *ortho*‐CH bond of the phenyl groups. Interestingly, the N═N scission of *trans*‐azobenzene can be achieved by a dinuclear Al^II^─Al^II^ complex featuring a chelating diamide ligand [(2,6‐*^i^* Pr_2_C_6_H_3_)NC(Me)]_2_^[^ ^22^ ^]^ and the disilyne [R´SiSiR´]^[^ ^23^ ^]^ (R´ = Si*^i^* Pr[CH(SiMe_3_)_2_]_2_) (Scheme 1a), which mimic subvalent 3d transition‐metal complexes.

Notably, divalent group‐14 compounds such as silylenes and their heavy analogs have been shown to activate *trans*‐azobenzene under mild conditions.^[^ ^24^, ^25^, ^26^, ^27^ ^]^ While these species readily bind azobenzene and induce *ortho*‐CH bond activation on the phenyl ring, the N─N σ‐bond of azobenzene is typically preserved. In 1998, Weidenbruch and co‐workers reported a reaction in which the photolytically generated dimesitylsilylene reacted with *trans*‐azobenzene to form an intermediate containing a SiN_2_ three‐membered ring, followed by the insertion of the N−N single bond into the *ortho‐CH* bond of the phenyl ring (Scheme 1b).^[^ ^28^ ^]^ Moreover, the Power group described a related germylene‐induced cleavage of *trans*‐azobenzene, also involving *ortho‐*CH activation.^[^ ^29^ ^]^ To the best of our knowledge, the cleavage of the N═N double bond in azobenzene under mild conditions—without concomitant *ortho*‐CH activation—by an isolable silylene remains unknown.

In recent years, our group has successfully designed and synthesized a series of isolable chelating bis‐NHSi (NHSi = *N*‐heterocyclic silylene) scaffolds featuring two divalent silicon centers with varying Si···Si distances for the activation of inert chemical bonds^[^ ^30^, ^31^, ^32^, ^33^ ^]^ and for the stabilization of low‐valent main‐group elements.^[^ ^34^, ^35^, ^36^ ^]^ Notably, the aniline‐based bis‐silylene PhN(LSi:)_2_ [L = PhC(*^t^* BuN)_2_]^[^ ^37^ ^]^ (**1**) with a Si···Si distance of 2.9 Å and the xanthene‐based XT(LSi:)_2_ (XT = 9,9‐dimethyl‐9H‐xanthene‐4,5‐diyl)^[^ ^38^ ^]^ (**2**) with a Si···Si distance of 4.3 Å have exhibited an intriguing reactivity toward small molecules, including the unusual adduct formation with PhC≡CPh and CO activation,^[^ ^31^, ^33^ ^]^ respectively. This prompted us to study whether the *trans*‐ and *cis*‐isomers of azobenzene show a distinct reactivity and the different Si···Si distances in **1** and **2** will have an impact on the nature of activation products. Herein, we report the quite different outcome of the N═N bond activation in *trans*‐ vs. *cis*‐azobenzene by bis‐silylenes **1** and **2**.

## Results and Discussion

### Activation of Azobenzene with Bis(silylene) 1

**Scheme 2:**
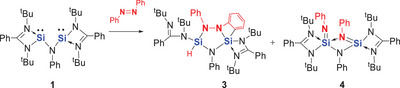
Reaction of aniline‐based bis‐silylene **1** and *trans*‐azobenzene.

Treatment of *trans*‐azobenzene with an equimolar amount of bis‐silylene **1** in THF at room temperature led to an orange solution after one week. Compound **3** was isolated from the resulting mixture as colorless crystals in 31% yield after work‐up (Scheme 2). Further work‐up enabled the isolation of the N═N cleavage product **4** in 25% yield.

**Figure 1:**
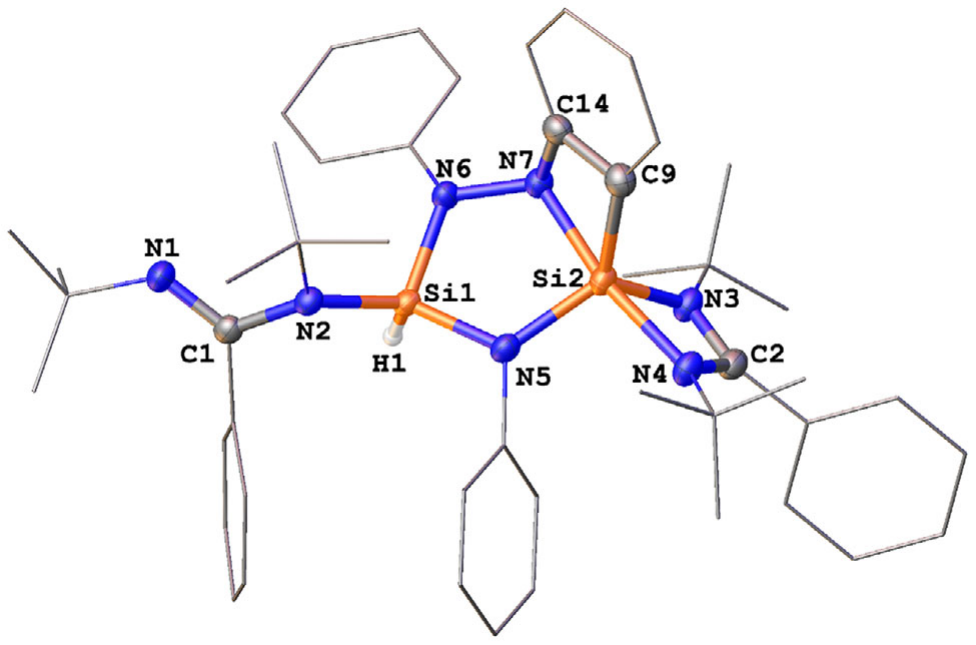
Molecular structure of **3**.^[^ ^44^ ^]^ Thermal ellipsoids are drawn at the 50% probability level. H atoms, except Si─H, and solvent molecules are omitted for clarity. Selected bond lengths (Å) and angles (°): Si1─N2 1.746(1), Si1─N5 1.729(1), Si1─N6 1.741(1), Si2─N5 1.759(1), Si2─N3 1.828(1), Si2─N4 1.931(1), Si2─N7 1.873(1), Si2─C9 1.884(2), C1─N1 1.268(2), C1─N2 1.431(2), N7─Si2─N4 169.55(5), N3─Si2─N5 117.30(6), C9─Si2─N5 123.68(6), N3─Si2─C9 118.90(6).

The molecular structure of **3** was established with a single‐crystal X‐ray diffraction analysis (scXRD) (Figure 1). Compound **3** features fused five‐ and four‐membered Si_2_N_3_ and SINC_2_ rings. The structure results from the cycloaddition of the N═N bond of *trans*‐azobenzene with **1**, accompanied by *ortho*‐CH activation of a phenyl ring. Consequently, the two silicon centers adopt distinct geometries: one is four‐coordinate with a tetrahedral configuration, while the other is five‐coordinate with a distorted trigonal bipyramidal geometry. The Si─H bond formation in **3** is confirmed by ^1^H NMR spectroscopy with a singlet at δ = 5.36 ppm and ^29^Si satellites (^1^ *J*_SiH_ = 258 Hz) and by IR spectroscopy with a Si─H stretching vibration mode at 2240 cm^−1^. The corresponding ^29^Si{^1^H} NMR resonance appears at δ = −32.4 ppm.

**Scheme 3:**
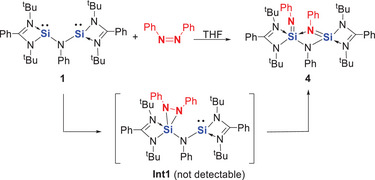
Formation of **4** from the aniline‐based bis‐silylene **1** and *cis*‐azobenzene via the proposed intermediate **Int1**.

**Figure 2:**
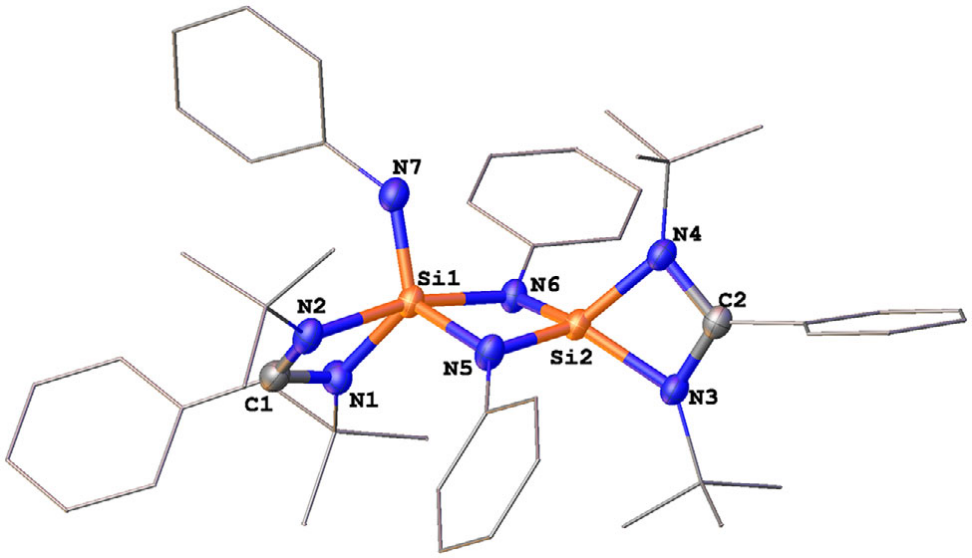
Molecular structures of **4** (two molecules in the asymmetric unit are observed; only one is shown).^[^ ^44^ ^]^ Thermal ellipsoids are drawn at the 50% probability level. H atoms and solvent molecules are omitted for clarity. Selected bond lengths (Å) and angles (°) (the data in brackets are for the other molecule): Si─N1 1.851(1) [1.859(1)], Si1─N2 2.007(1) [1.983(1)], Si1─N7 1.657(1) [1.656(1)], Si1─N5 1.818(1) [1.824(1)], Si1─N6 1.912(1) [1.912(1)], Si2─N3 1.793(1) [1.794(1)], Si2─N4 1.798(1) [1.799(1)], Si2─N5 1.680(1) [1.674(1)], Si2─N6 1.665(1) [1.663(1)], N7─Si1─N1 123.06(7) [123.56(5)], N7─Si1─N2 96.82(6) [97.65(6)], N7─Si1─N5 120.17(7) [121.96(7)], N7─Si1─N6 96.03(6) [96.18(6)], N4─Si2─N3 73.13(6) [73.22(6)], N5─Si2─N6 90.92(6) [91.15(6)], N4─Si2─N6 122.66(6) [123.17(6)], N3─Si2─N5 119.13(6) [118.67(6)], N6─Si1─N2167.15(6) [166.13(6)].

While the reaction of **1** with *trans*‐azobenzene afforded the N═N bond cleavage product **4** in only 25% yield, interestingly, treatment of **1** in THF with *cis*‐azobenzene, prepared from the *trans*‐isomer,^[^ ^39^ ^]^ furnished compound **4** quantitatively (Scheme 3). Remarkably, the reaction is complete within a few seconds at room temperature, yielding orange crystals of **4** in 78% isolated yield. The scXRD analysis of **4** confirmed its molecular structure, revealing two distinct silaimine moieties featuring terminal and bridging Si═N units (Figure 2). One silaimine silicon atom is four‐coordinate, adopting a tetrahedral geometry, while the other is five‐coordinate with a distorted trigonal‐bipyramidal configuration. The Si1─N7 (1.657(1) Å), Si2─N5 (1.680(1) Å), and Si2–N6 (1.665(1) Å) bonds are significantly shorter than the other Si─N bonds (ranging from 1.793(1) to 2.007(1) Å) in this molecule, suggesting a partial Si─N double‐bond character. Similar Si═NAr moieties with Si─N distances of 1.589(5) and 1.581(1) Å have been previously reported.^[^ ^40^, ^41^ ^]^

Although the two silicon atoms adopt distinct coordination geometries in the solid state, the ^1^H NMR spectrum of **4** recorded in *d_8_*‐THF shows only one single resonance (δ 1.11 ppm) for the four *^t^* Bu groups. Similarly, only one signal at −82.74 ppm appeared in the ^29^Si{^1^H} NMR spectrum at room temperature (Figure S9a *, bottom*). Moreover, the ^13^C{^1^H} NMR spectrum of **4** (Figure S8) exhibits only two sets of resonances for the five Ph groups, indicating that all three NPh groups and both CPh groups in **4** are dynamically equivalent at room temperature, respectively. This observation can be attributed to rapid intramolecular coordination exchange between the PhN groups and/or the *^t^* BuN groups at both Si centers in solution at room temperature. A similar situation has been previously observed by us for related amidinato silicon systems.^[^ ^42^ ^]^ However, at low temperature (208 K), two signals at δ −107.59 and −111.88 ppm in the ^29^Si{^1^H} NMR spectrum of **4** in *d*_8_‐THF appeared (Figure S9a *, top*). In line with that, two signals can also be observed at δ −44.66 and −121.38 ppm in the solid‐state ^29^Si NMR spectrum (Figure S9b).

### Activation of Azobenzene with Bis(silylene) 2

**Scheme 4:**
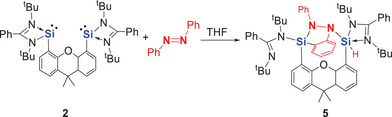
Reaction of xanthene‐based bis(siylene) **2** with *trans*‐azobenzene to furnish the C─H activation product **5**.

To investigate the influence of the Si···Si distance in bis‐silylenes on N═N bond activation and to better understand the mechanism of its cleavage, we also explored the reaction of azobenzenes with the xanthene‐based bis‐silylene **2**, which features a larger Si···Si separation (4.3 vs. 2.9 Å in **1**). We first examined the reaction of **2** with *trans*‐azobenzene (Scheme 4). Compared to the aniline‐based bis‐silylene **1**, the xanthene‐based system reacts much more rapidly with *trans*‐azobenzene. In fact, in THF, the activation occurs instantly even at −20 °C, yielding compound **5** as colorless crystals in a 69% isolated yield.

**Figure 3:**
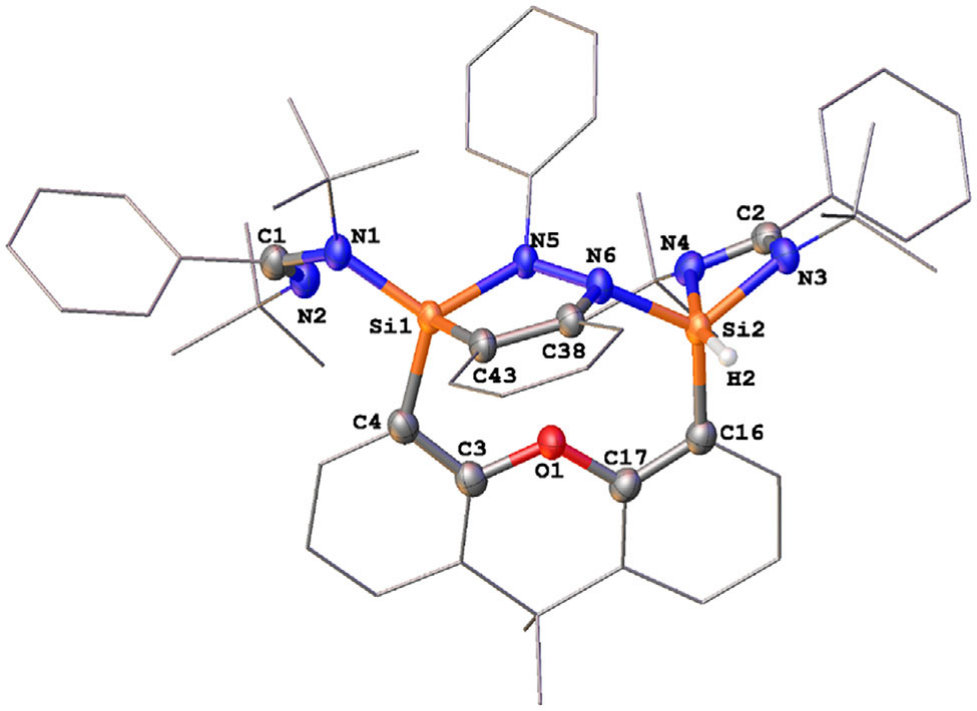
Molecular structure of **5**.^[^ ^44^ ^]^ Thermal ellipsoids are drawn at the 50% probability level. H atoms, except the SiH, and solvent molecules are omitted for clarity. Selected bond lengths (Å) and angles (°): Si1─N1 1.749(2), Si1─N5 1.785(1), Si1─C4 1.891(2), Si1─C43 1.846(2), Si2─N3 1.787(1), Si2─N4 2.207(2), Si2─N6 1.785(2), Si2─C16 1.888(2), Si2─H2 1.34(2), N4─Si2─H2 162.8(8), N5─Si1─N1 115.46(7), N1─Si1─C4 112.9(1), C4─Si1─N5 119.05(8), C4─Si1─C43 103.69(8), N3─Si2─N6 119.52(7), N6─Si2─C16 121.53(8), N3─Si2─C16 115.68(8).

The formation of a Si─H bond in **5** is confirmed by ^1^H NMR (δ = 6.54 ppm, Si satellites: ^1^ *J*_SiH_ = 243 Hz), ^29^Si NMR (δ = −38.38 ppm), IR (*v*_Si─H _= 2192 cm⁻¹), and scXRD (Figure 3). The molecular structure of **5** reveals an end‐on binding of the N─N bond of azobenzene by the two silicon centers of **2** along with an *ortho*‐C─H activation of one phenyl ring.

**Scheme 5:**
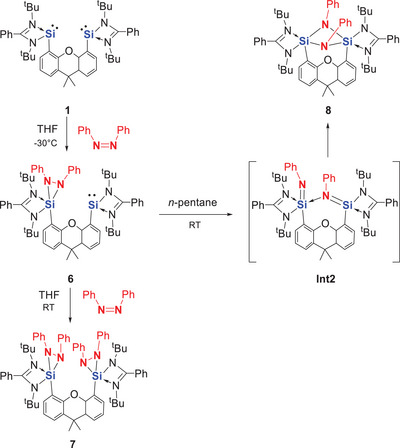
N═N bond cleavage mediated by the xanthene‐based bis‐silylene **2** to afford **8** via **6** and the proposed intermediate **Int2**. The reaction of **6** with an additional equivalent of *cis*‐azobenzene leads to **7**.

**Figure 4:**
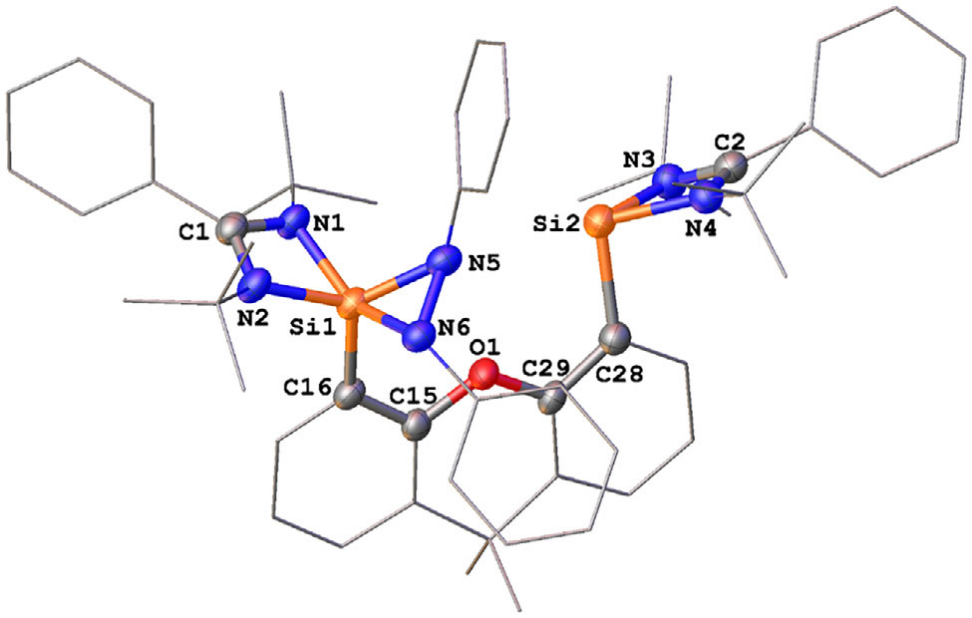
Molecular structure of **6**.^[^ ^44^ ^]^ Thermal ellipsoids are drawn at the 50% probability level. H atoms and solvent molecules are omitted for clarity. Selected bond lengths (Å) and angles (°): Si1─N1 1.908(2), Si1─N2 1.816(2), Si1─N5 1.727(2), Si1─N6 1.810(2), Si1─C16 1.870(2), Si2─N3 1.901(2), Si2─N4 1.889(2), Si2─C28 1.963(3), N5─N6 1.525(3) N6─Si1─N1 152.4(1), N2─Si1─C16 112.0(1), C16─Si1─N5 122.3(1), N2─Si1─N5 123.8(1), N3─Si2─C28 95.8(1), N4─Si2─C28 99.4(1), N3─Si2─N4 68.53(9).

We then investigated the reactivity of **2** toward *cis*‐azobenzene. Since the equimolar reaction at room temperature yielded a mixture, we carried out the reaction in THF at −30 °C by adding a solution of one molar equivalent of *cis*‐azobenzene in THF to a solution of **2** in THF. Interestingly, this led to a clean reaction, affording compound **6** as yellow crystals in 68% isolated yield after work‐up (Scheme 5). An scXRD analysis revealed that only one silylene moiety of **2** reacted, forming a SiN_2_ three‐membered ring (Figure 4). Compound **6** results from a [1+2]‐cycloaddition reaction, reminiscent of the product obtained from the *tert*‐butyl substituted amidinatosilylene L(*^t^* Bu)Si: with azobenzene, as previously reported by Roesky and co‐workers.^[^ ^24^ ^]^ In the molecular structure of **6**, the reacted silicon atom (Si1) is five‐coordinate, and the N─N bond becomes a single bond (1.525(3) Å), closely resembling the activation product mediated by L(*^t^* Bu)Si: (1.524(2) Å).^[^ ^24^ ^]^ Due to the steric hindrance, the SiN_2_ ring is oriented away from the Si_2_ atom, causing the loss of mirror‐plane symmetry. Consistent with this, the ^1^H NMR spectrum of **6** exhibits four singlets at δ 0.98, 1.17, 1.27, and 1.46 ppm for the four distinct *^t^* Bu groups, along with two singlets at δ = 1.44 and 1.53 ppm for the two methyl groups on the xanthene backbone. In the ^29^Si{^1^H} NMR spectrum, a singlet at δ *=* 14.83 ppm corresponds to the Si2 nucleus in the three‐coordinate silylene moiety, comparable to that in **2** (δ = 17.3 ppm).^[^ ^38^ ^]^ Notably, the five‐coordinate Si1 atom gives rise to a signal at δ = −115.84 ppm, significantly shifted upfield with respect to the activation product mediated by L(*^t^* Bu)Si: (δ = −99.6 ppm).^[^ ^24^ ^]^

**Figure 5:**
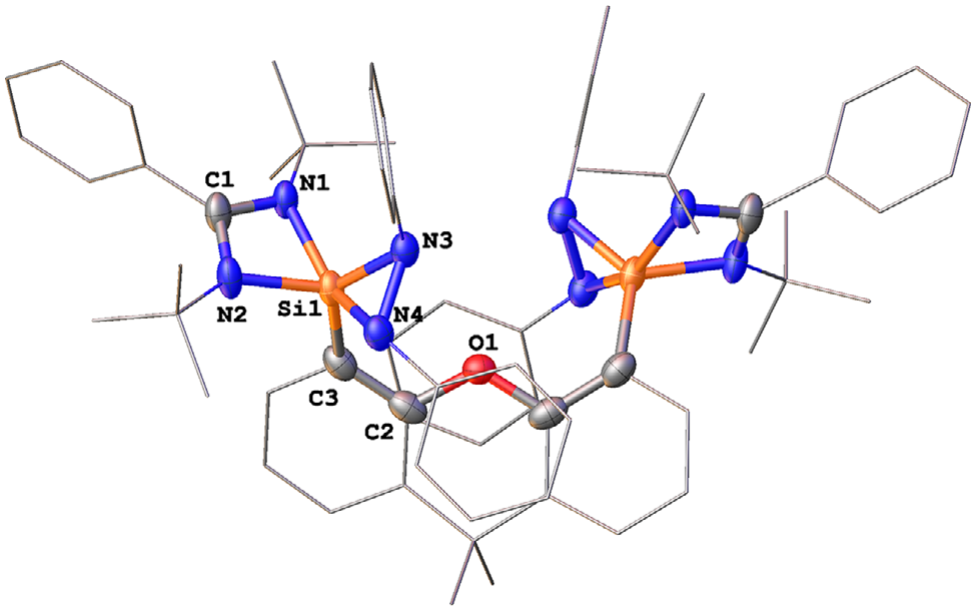
Molecular structure of **7**.^[^ ^44^ ^]^ Thermal ellipsoids are drawn at the 50% probability level. H atoms and solvent molecules are omitted for clarity. Selected bond lengths (Å) and angles (°): Si1─N1 1.899(1), Si1─N2 1.815(1), Si1─N3 1.746(1), Si1─N4 1.784(1), Si1─C3 1.880(2), N4─Si1─N1 150.92(6), N2─Si1─C3 111.18(7), C3─Si1─N3 128.90(7), N2─Si1─N3 111.95(7).

Since compound **6** retains one amidinato silylene moiety, an additional equivalent of *cis*‐azobenzene was introduced to a THF solution of **6** at room temperature, yielding compound **7** in 82% isolated yield (Scheme 5). Alternatively, compound **7** can be readily obtained by reacting bis‐silylene **2** with two molar equivalents of *cis*‐azobenzene in THF at room temperature. Compound **7** has also been fully characterized. In the ^1^H NMR spectrum, only two singlets appear at δ = 1.02 and 1.46 ppm for the four *^t^Bu* groups, along with only one signal at δ = 1.44 ppm for the two methyl groups. Consistently, the ^29^Si{^1^H} NMR spectrum shows only one signet at δ *=* −115.28 ppm, corresponding to the five‐coordinate Si atoms. Crystallization of **7** from diethyl ether solutions afforded colorless rods. An scXRD analysis revealed two five‐coordinate Si atoms, each featuring a SiN_2_ three‐membered ring and adopting a distorted trigonal‐bipyramidal coordination geometry (Figure 5). The structure parameters of both SiN_2_ rings are comparable to those observed in **6**.

**Figure 6:**
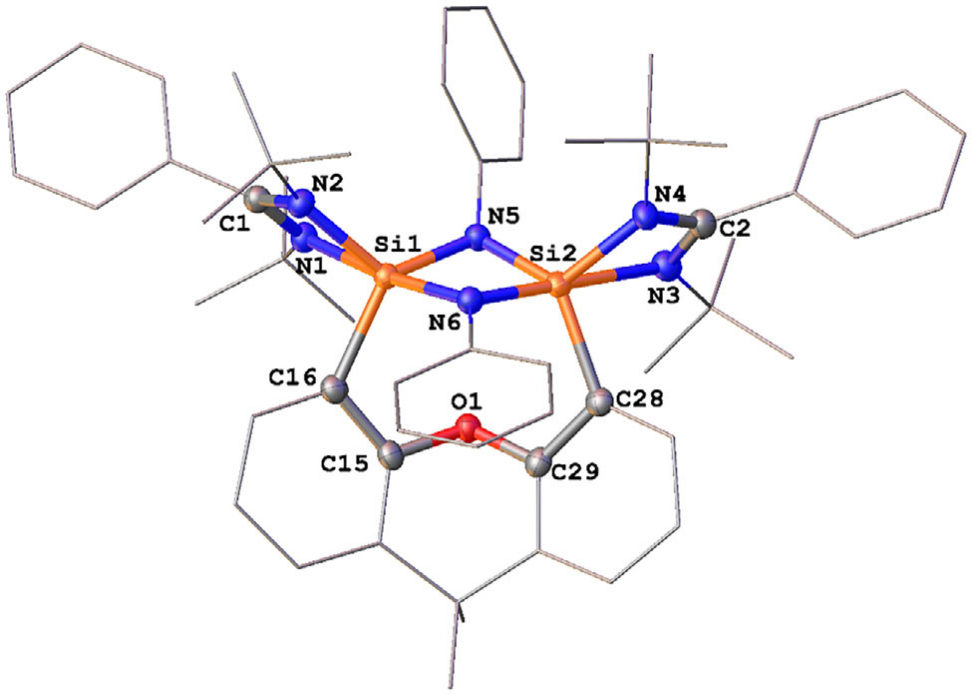
Molecular structure of **8**.^[^ ^44^ ^]^ Thermal ellipsoids are drawn at the 50% probability level. H atoms and solvent molecules are omitted for clarity. Selected bond lengths (Å) and angles (°): Si1─N1 2.028(1), Si1─N2 1.840(1), Si1─N5 1.771(1), Si1─N6 1.823(1), Si1─C16 1.919(1), Si2─N3 2.260(1), Si2─N4 1.799(1), Si2─N5 1.750(1), Si2─N6 1.795(1), Si2─C28 1.915(1), N6─Si1─N1 172.38(4), N2─Si1─C16 105.72(5), C16─Si1─N5 127.96(5), N2─Si1─N5 122.35(5), N3─Si2─N6 177.73(4), N4─Si2─C28 108.30(5), N5─Si2─C28 129.23(5), N4─Si2─N5 117.05(5).

At room temperature, the yellow compound **6** remains stable in the solid state under an inert atmosphere. Interestingly, in *n*‐pentane solution, it slowly converts to compound **8**. After several days, the conversion is complete, yielding colorless crystals of **8** in 85% isolated yield (Scheme 5). Compound **8** crystallized in the monoclinic space group *P*2₁/*n*. A scXRD analysis revealed a strongly folded xanthene backbone with a Si(μ‐NPh)₂Si core, coordinated by two amidinates supported by the xanthene backbone (Figure 6). Both Si atoms are thus five‐coordinate, adopting a distorted trigonal bipyramidal geometry, with N1─Si1─N6 (172.38(4)°) and N3─Si2─N6 (177.73(4)°) as the respective axial angles. Aside from the exceptionally long dative N3→Si2 [2.260(1) Å] and N1→Si1 [2.028(1) Å] bonds, all other Si─N distances, ranging from 1.750(1) to 1.840(1) Å, fall within the typical range for Si─N single bonds. The N5─N6 distance of 2.333 Å indicates complete cleavage of the N═N double bond of *cis*‐azobenzene. Due to the strongly bent xanthene backbone, the two NPh groups in the molecule are magnetically inequivalent. In the ¹H NMR spectrum of **8**, two broad signals resonate at δ = 1.05 and 1.36 ppm for the four *tert*‐butyl groups, along with two singlets at δ = 1.48 and 1.66 ppm for the two methyl groups on the xanthene are observed. The ^2^⁹Si{¹H} NMR spectrum shows a singlet at δ = −81.59 ppm for both Si atoms, consistent with five‐coordinate silicon centers.

### Density Functional Theory Calculations

To gain insight into the electronic structures of the N═N bond cleavage products and the reaction mechanism, density functional theory (DFT) calculations were performed at the SMD‐B3LYP‐D3(BJ)/def2‐TZVP//B3LYP‐D3(BJ)/def2‐SVP level of theory (see Supporting Information for details). The optimized structures obtained from the calculations show good agreement with the experimental XRD structures (Tables S13 and S14).

**Figure 7:**
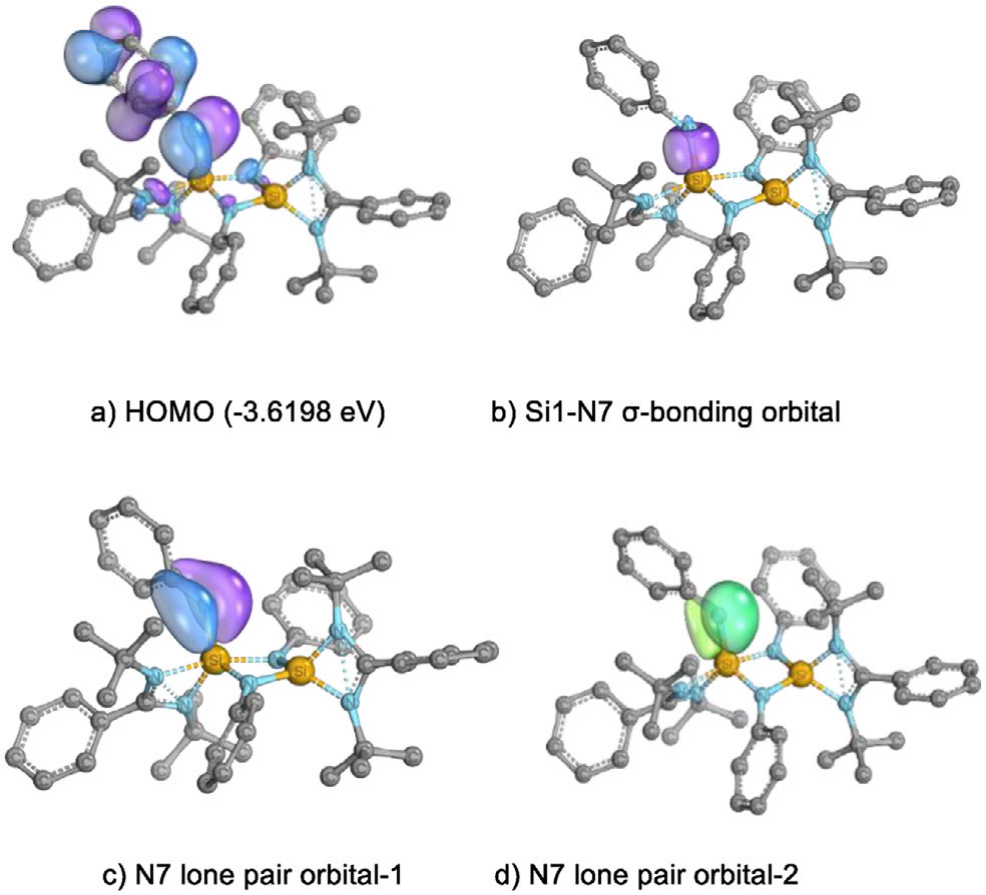
Depiction of the HOMO and selected IBOs of **4**.

In compound **4**, the Wiberg Bond Indices (WBIs) of the Si1─N7 (1.10), Si2─N5 (0.92), and Si2─N6 (1.03) bonds are significantly higher than those of the other Si─N bonds (0.43–0.64), indicating partial Si═N character (Table S13). Consistently, intrinsic bond orbital (IBO)^[^ ^43^ ^]^ calculations reveal that, in addition to the σ‐bond of Si1─N7, the lone pair of electrons on N7 delocalizes toward the Si1 atom, contributing to the highest occupied molecular orbital ([HOMO], Figure 7). Similarly, the delocalization of lone pairs on N5 and N6 toward Si2 contributes to the partial double‐bond character of the Si2─N5 and Si2─N6 bonds (Figure S34). In contrast, the WBIs of the Si─N bonds in the four‐membered ring of compound **8** range from 0.69 to 0.80 and are relatively uniform (Table S14). With only minor π‐back donation to the distal silicon atoms (Figure S35), these Si─N bonds should be considered as single bonds.

**Figure 8:**
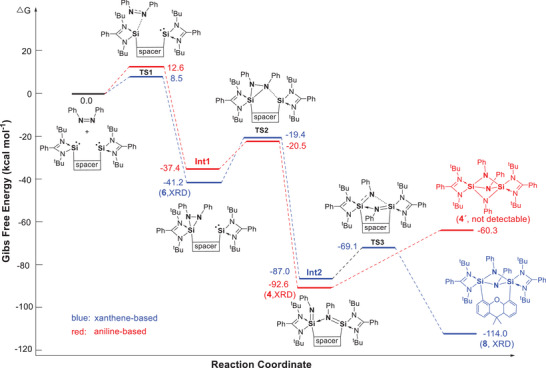
Proposed mechanism for reactions of **1** and **2** with *cis*‐azobenzene, respectively, leading to N═N cleavage product **4** (via **Int1**) and **8** (via **6** and **Int2**). The proposed product **4´** from aniline‐based **1**, corresponding to **8** from xanthene‐based **2**, is not detectable.

The DFT‐derived N═N bond cleavage mechanisms are depicted in Figure 8. For the N═N bond cleavage mediated by xanthene‐based bis‐silylene **2**, the calculations suggest a pathway that begins with a [1+2]‐cycloaddition product as the first intermediate (**Int1**, Δ*G* = −41.2 kcal mol^−1^), followed by the N═N bond cleavage product as the second intermediate (**Int2**, Δ*G* = −82.0 kcal mol^−1^). The latter involves the transition states **TS1** and **TS2** with reasonable energy barriers of 8.5 and 21.8 kcal mol^−1^, respectively. Notably, **Int1** has been isolated as compound **6**. In contrast, **Int2** is not experimentally detectable, likely due to the relatively small energy barrier to **TS3** (17.9 kcal mol^−1^) for its subsequent transformation into compound **8**, which is highly exergonic (Δ*G* = −27 kcal mol^−1^).

A comparable mechanism is proposed for the reaction with aniline‐based bis‐silylene **1**, where the corresponding species **4** is also isolable. Similar to the first step of *cis*‐azobenzene activation by bis‐silylene **2**, this reaction proceeds via an intermediate (**Int1**) with an activation energy of 12.6 kcal mol^−1^. However, **Int1** is less stable than **6**, likely due to the closer proximity of the silylene moieties compared to the xanthene‐based system in **6**. With a low barrier of 16.9 kcal mol^−1^, it undergoes clean N═N bond cleavage to yield compound **4** as the final activation product.

It is worth noting that further isomerization of **4** to form **4′**—analogous to the conversion of **6** into **8**—is not observed experimentally. DFT calculations suggest that the hypothetical compound **4′** is less stable than **4**, and its conversion is endothermic with a Gibbs free energy change (Δ*G*) of +32.3 kcal mol^−1^, making the transformation thermodynamically unfavorable.

The reaction of bis‐silylenes **1** and **2** with *trans*‐azobenzene to give **3** and **5**, respectively, proceeds via cycloaddition of the N═N bond to the two chelating Si(II) centers of the bis‐silylenes, accompanied by *ortho*‐CH activation of one phenyl ring of the azobenzene. The detailed mechanism of the cycloaddition, C─H activation, as well as the formation of **4** from **1** and *trans*‐azobenzene, appears more complex and remains not fully understood. Thermodynamically, the formation of **3** and **5** is exergonic, with Δ*G* of −43.4 and −46.8 kcal mol^−^¹, respectively (Table S15).

Although both **3** and **5** exhibit similar end‐on coordination of the N─N bond between the two silicon centers, their structures differ: **3** contains a four‐membered SiNC₂ ring, whereas **5** features a five‐membered SiN₂C₂ ring. To gain deeper insight into the regioselectivity of these activation products, hypothetical isomers—**3´** with a five‐membered SiN₂C₂ ring and **5** with a four‐membered SiNC₂ ring — were optimized. However, both **3´** (Δ*G* = −28.1 kcal mol⁻¹) and **5´** (Δ*G* = −33.2 kcal mol⁻¹) are thermodynamically less favorable, by approximately 15 kcal mol⁻¹, compared to the experimentally observed isomers **3** and **5**. The distinct reaction outcomes are likely attributable to the different Si···Si distances in bis‐silylenes **1** (2.9 Å) and **2** (4.3 Å).

## Conclusion

In summary, we have demonstrated the quite different outcome of cooperative N═N bond activation and cleavage of *trans*‐ vs. *cis*‐azobenzene employing bis‐silylenes with distinct Si···Si intramolecular distances. While both bis‐silylenes, aniline‐based **1** and xanthene‐based **2**, efficiently engage in *ortho*‐C─H activation of *trans*‐azobenzene, only their cooperative action engenders N═N bond cleavage of *cis*‐azobenzene. With bis‐silylene **1**, the reaction with *cis*‐azobenzene at room temperature rapidly yields the N═N cleavage product **4**, featuring a bis‐silaimine. In contrast, the activation of *cis*‐azobenzene with bis‐silylene **2** is more controllable; at low temperature, it allows for the isolation of compound **6**, which contains a stable three‐membered SiN₂ ring (azadisilirane). The subsequent reaction of **6** with an additional equivalent of *cis*‐azobenzene affords compound **7**, incorporating two SiN₂ rings. Notably, **6** undergoes slow N═N bond cleavage at room temperature, yielding the final activation product **8**. DFT calculations provide detailed mechanistic insights, supporting a stepwise Si^II^···Si^II^ cooperative activation pathway with accessible energy barriers. The ability to isolate and characterize key intermediates offers valuable experimental validation of the proposed mechanism, further underscoring the role of bis‐silylenes in controlled bond activation. Overall, this study advances our understanding of N═N bond activation by low‐valent silicon species and highlights the potential of bis‐silylenes as cooperative reagents for inert bonds. These findings open new avenues for exploring bis‐silylenes in selective bond activation and N═N functionalization.

## Supporting Information

The authors have cited additional references within the Supporting Information.^[^ ^39^, ^43^, ^45^, ^46^, ^47^, ^48^, ^49^, ^50^, ^51^, ^52^, ^53^, ^54^, ^55^, ^56^, ^57^ ^]^

## Conflict of Interests

The authors declare no conflict of interest.

## Supporting information



Supporting Information

## Data Availability

The data that support the findings of this study are available in the Supporting Information of this article.
