# Supplementary material for: Facile N═N Bond Cleavage of Cis‐Azobenzene with Bis‐silylenes
Source: Angew Chem Int Ed Engl. 2025 May 10;64(27):e202507560. doi: 10.1002/anie.202507560 (PMC12207375; doi:10.1002/anie.202507560)
Supplement: Supplementary file 1 — Supporting Information [file ANIE-64-e202507560-s001.pdf]

Supporting Information

Facile N=N Bond Cleavage of cis-*Azobenzene with Bis-silylenes*

Yun Xiong, Shenglai Yao, and Matthias Driess*

Table of Content

A. Experimental Section……………………………………….………………...…..……...2

A1 General Considerations……………………………………….……….…….……….……2

A2 Single-Crystal X-ray Structure Determination………………………...…..….……….…..2

A3 Synthesis and Characterization………………………………….……………..……….….3

A4 Details of the Single Crystal X-ray Diffraction Analyses……………………….……..…….…34

B. Computational Details …………….…………….……………….….….….………....……..47

C. References…………………………………………………….……….….….…..…...……..98

A. Experimental Section

A1. General Considerations

All experiments were carried out under dry oxygen-free nitrogen using standard Schlenk techniques or an MBraun glove box fitted with a gas purification and recirculation unit. Solvents were dried by standard methods and freshly distilled prior to use. The starting material aniline-based bis(silylene) PhN(LSi:)$_2$ (**1**) (L = PhC(tBuN)$_2$) and xanthene-based bis(silylene) XT(LSi:)$_2$ (**2**) (XT = 9,9-dimethyl-xanthene-4,5-diyl) were prepared according to our previous work.[1,2] Trans-azobenzene was purchased from Alfa Aesar. The NMR spectra were recorded with Bruker spectrometers Avance II 400, Avance III 400, and Avance III 500 referenced to residual solvent signals as internal standards. Abbreviations: *s* = singlet; *d* = doublet; *t* = triplet; *sept* = septet; *m* = multiplet; *br* = broad. High-resolution ESI-MS were measured on a Thermo Scientific LTQ orbitrap XL. IR spectra were measured with a Nicolet iS5 FT-IR-Spectrometer from the company of Thermo Scientific. Melting points were measured on a Stuart SMP30 melting point apparatus.

A2. Single-Crystal X-ray Structure Determination

The crystals were mounted on a glass capillary in per-fluorinated oil and measured in a cold N_2 flow. The data of compounds **3**, **4**, **5**, **6**, **7**, and **8** were collected on an Oxford Diffraction Supernova, Single source at offset, Atlas at 150 K (Cu- Kα-radiation, λ = 1.5418 Å). The structures were solved with the SHELXT[3] and refined with Olex2[4,5] software package. The positions of the H atoms were calculated and considered isotropically according to a riding model. In the molecular structure of compound **3**, one of the phenyl groups is disordered over two orientations with an occupancy ratio of 0.55:0.45. In the molecular structure of compound **4**, two independent molecules are present in the asymmetric unit. One of the tert-butyl groups is disordered over two orientations with an occupancy ratio of 0.54:0.46, while one phenyl group disordered over two orientations with an occupancy ratio of 0.57:0.43. Additionally, one and a half benzene molecules can be found in the asymmetric unit, but they are severely disordered and has been removed with the solvent-mask procedure in Olex2.[4,5] In the crystal structure of **6**, two pentane solvent molecules are found in the asymmetric unit with severe disorder which have been removed with the solvent-mask procedure. Similarly, in the crystal structure of **7**, there are 1.2 diethyl ether solvent molecules in the asymmetric unit with severe disorder. They have been removed with the solvent-mask procedure. In the crystal structure of **8**, there is one severe disordered diethyl ether solvent molecule in the asymmetric unit, which has been removed with the solvent-mask procedure, too. CCDC 2440233 (compound **3**), 2440234 (compound **4**), 2440231 (compound **5**), 2440232 (compound **6**), 2440235 (compound **7**) and 2440236 (compound **8**) contain the supplementary crystallographic data for this paper. These data can be obtained free of charge by contacting The Cambridge Crystallographic Data Centre, 12, Union Road, Cambridge CB2 1EZ, UK; fax: +44 1223 336033.

A3. Synthesis and Characterization

***Cis*-azobenzene**: *Cis*-azobenzene was prepared with modified literature procedure. [6] *Trans*-azobenzene (1.62 g, 8.89 mmol) was dissolved in *n*-hexane (70 mL) and then irradiated at a distance of approximately 5 cm using a LUMATEC Superlite 400 light source. The irradiation was performed within the wavelength range of 320-400 nm for 2 hours. The resulted mixture of *trans*- and *cis*-azobenzene in *n*-hexane was separated via column chromatography on alumina. Initially, the *trans*-azobenzene was eluted from the column with *n*-hexane, afterwards, *cis*-azobenzene was collected by washing the column with a mixture of *n*-pentane and 10% methanol. The collected *cis*-azobenzene solution was worked-up in the dark at room temperature. Methanol was removed by washing with water, and the separated organic layer was dried over $MgSO_4$ and then filtered. The n-pentane solution containing cis-azobenzene was concentrated under vacuum and stored at -20 °C. After 2 hours, red crystals of cis-azobenzene were formed in the yield of 27% (0.43 g, 2.36 mmol). **^{1}H NMR** (400 MHz, C_6D_6, 298K) (Figure *S1a*, top): δ = 6.67 – 6.73 (*m*, 3 H, Ph-*H*), 6.80 - 6.84 ppm (*m*, 2 H, Ph-*H*). **^{13}C{^{1}H} NMR** (125 MHz, C_6D_6, 298K): δ = 120.34, 126.65, 128.47, 154.05 ppm.

In comparison, the ^{1}H NMR spectrum of the *trans*-azobenzene from Alfa Aesar was also measured (Figure *S1*, bottom). **^{1}H NMR** (400 MHz, C_6D_6, 298K): δ = 7.04 – 7.09 (*m*, 1 H, Ph-*H*), 7.13 – 7.17 (m, 2 H, Ph-*H*), 7.99 – 8.01 ppm (*m*, 2 H, Ph-*H*).

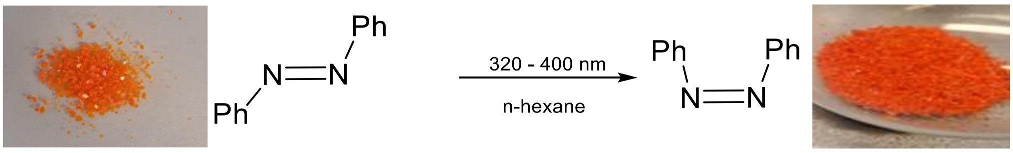

Scheme S1. Preparation of *cis*-azobenzene.

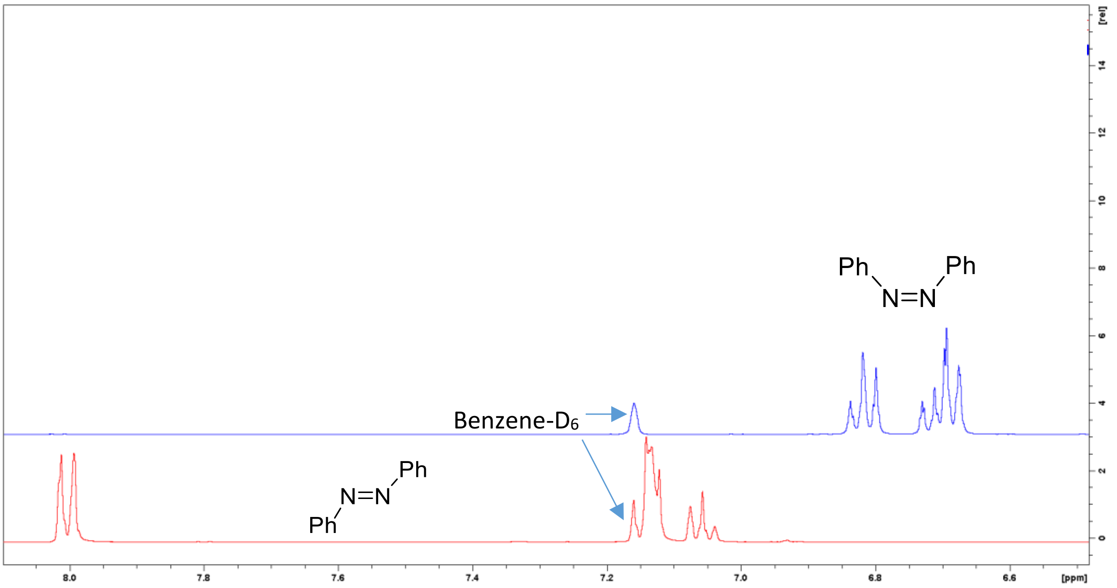

Figure S1. ^{1}H NMR spectra comparison: synthesized *cis*-azobenzene (top) and *trans*-azobenzene from Alfa Aesar (bottom) in C_6D_6.

Compound 3: To a Schlenk flask charged with yellow bis(silylene) PhN(LSi:)$_2$ (**1**) (0.84 g, 1.37 mmol) and orange *trans*-azobenzene (0.25 g, 1.37 mmol) was added 30 mL THF at room temperature with stirring. After one week, the reaction was complete. Volatiles were removed under vacuum, and the residue was extracted with n-hexane (30 mL). The colorless filtrate was concentrated to 10 mL and cooled at –20 °C for one day to afford compound **3** as colorless crystals (0.33 g, 0.42 mmol, 31%). After extraction of **3** with n-hexane, from the rest residue compound **4** can be obtained as an orange-red solid (0.27 g, 0.34 mmol, 25% yield). Characterization of **3**: **M.P.** 243 °C (decomp.). **^{1}H NMR** (400 MHz, C_6D_6, 298K): δ = 0.79 (*s*, 9 H, C(C*H*$_3$)$_3$), 1.11 (*s*, 9 H, C(C*H*$_3$)$_3$), 1.39 (*s*, 9 H, C(C*H*$_3$)$_3$), 1.65 (*s*, 9 H, C(C*H*$_3$)$_3$), 5.36 (*s*, Si-*H* with Si satellite: $^1J_{SiH}$ = 258 Hz), 6.79 – 7.08 (*m*, 17 H, arom.-*H*), 7.29 (*t*, $^3J_{(HH)}$ = 7.95 Hz, 2 H, arom.-*H*), 7.54 (*t*, $^3J_{(HH)}$ = 7.95 Hz, 2 H, arom.-*H*), 7.60 (*d*, $^3J_{(HH)}$ = 6.46 Hz, 1 H, arom.-*H*), 7.89 ppm (*d*, $^3J_{(HH)}$ = 7.88 Hz, 2 H, arom.-*H*); **^{13}C{^{1}H} NMR** (125 MHz, C_6D_6, 298K): δ = 30.12 (NC(*C*H$_3$)$_3$), 31.23 (NC(*C*H$_3$)$_3$), 31.44 (NC(*C*H$_3$)$_3$), 31.89 (NC(*C*H$_3$)$_3$), 52.87 (N*C*(CH$_3$)$_3$), 54.54 (N*C*(CH$_3$)$_3$), 54.97 (N*C*(CH$_3$)$_3$), 57.70 (N*C*(CH$_3$)$_3$), 113.26, 114.25, 117.68, 120.74, 123.99, 126.78, 127.01, 127.26, 127.55, 127.99, 128.20, 128.21, 128.90, 129.25, 129.47, 130.90, 132.06, 132.68, 132.99, 136.88, 141.12, 145.77, 150.90, 158.43, 165.74 (N*C*N), 170.80 ppm (N*C*N). **^{29}Si{^{1}H} NMR** (99 MHz, C_6D_6, 298 K): δ = -32.40 (*s*, *Si*-H), -94.26 (*s*, *Si*) ppm. **HR-ESI-MS: m/z**: 792.45898 (calc. 792.45997 [M+H]$^+$, m/z); **IR** (cm^{-1}): 3057.20(*w*), 2955.02(*m*), 2240.51(*w*, ν_{Si-H}), 1625.55(*m*), 1591.97(*m*), 1576.57(*m*), 1537.07(*w*), 1485.52(*s*), 1445.67(*w*), 1428.78(*m*), 1405.31(*s*), 1391.34(*w*), 1359.23(*m*), 1302.15(*w*), 1285.48(*w*), 1267.89(*w*), 1243.94(*m*), 1226.25(*s*), 1201.87(*m*), 1188.84(*m*), 1097.28(*m*), 1071.82(*w*), 1054.74(*w*), 1027.23(*m*), 990.51(*m*), 946.18(*vs*), 919.65(*w*), 907.67(*m*), 895.34(*m*), 886.07(*s*), 830.93(*w*), 798.52(*s*), 765.90(*s*), 745.96(*s*), 722.97(*w*), 705.79(*m*), 697.39(*s*), 690.92(*s*), 656.30(*w*), 644.88(*m*), 620.84(*m*), 585.10(*w*), 574.17(*w*).

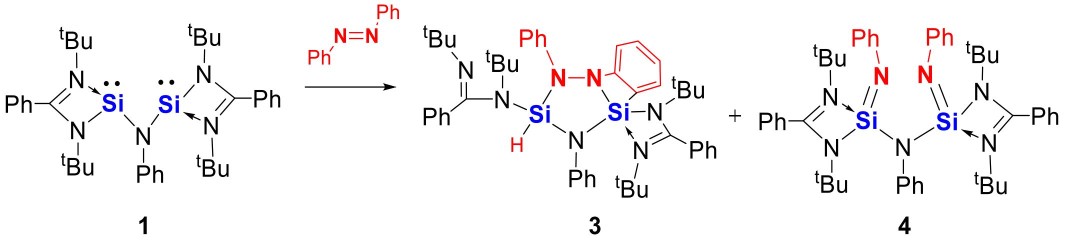

Scheme S2. Synthesis of **3**.

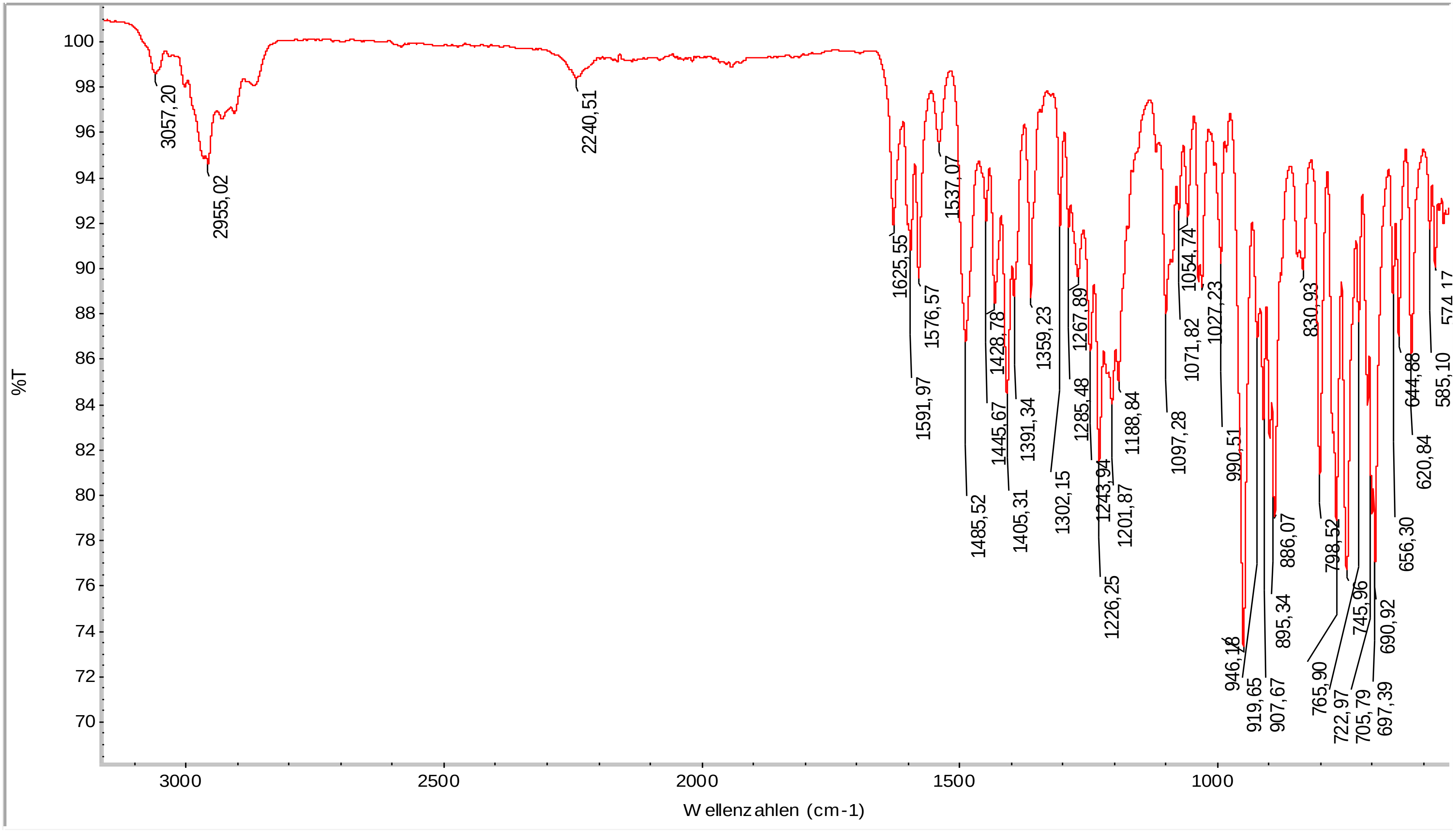

Figure S2. IR spectrum of compound 3.

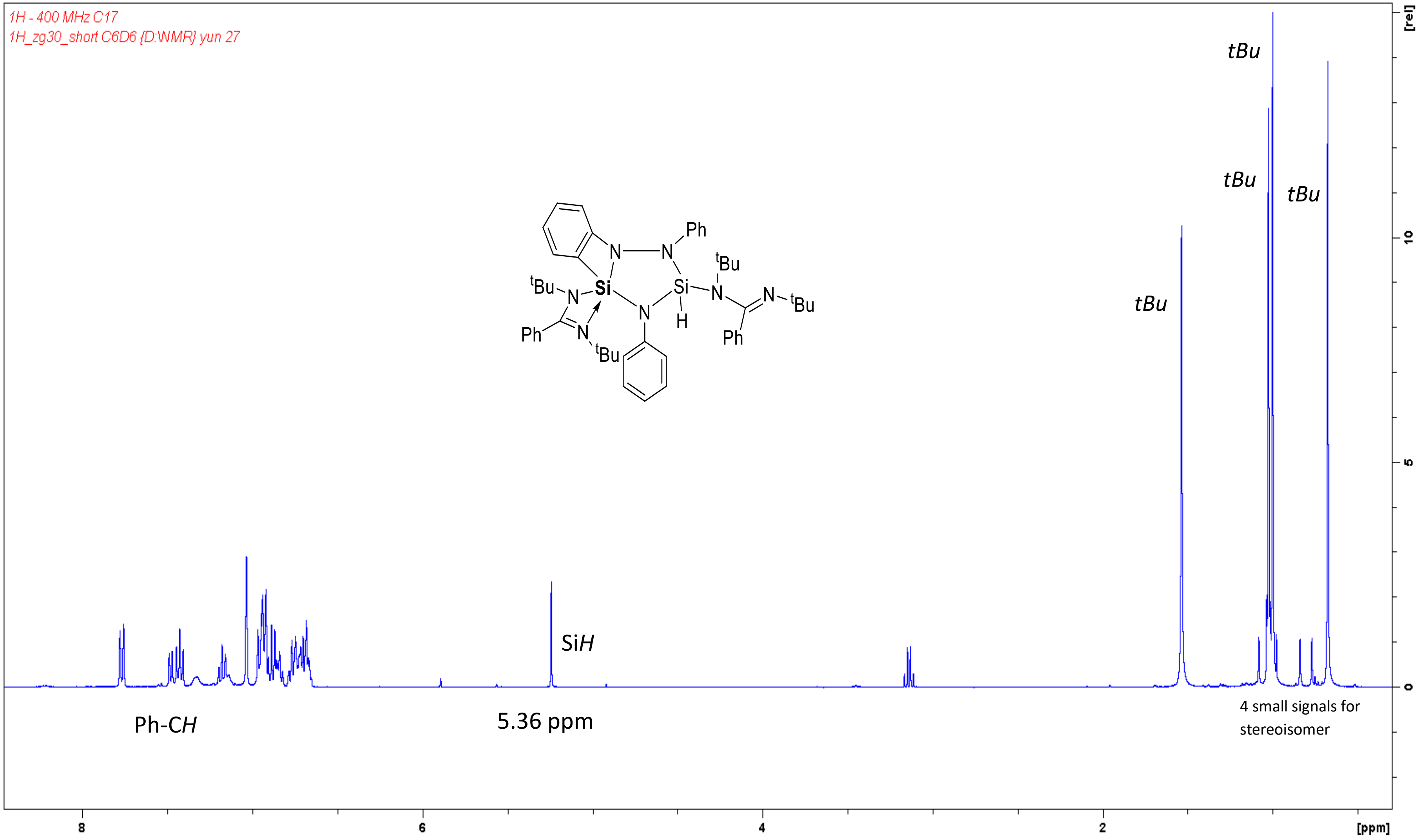

Figure S3. ^{1}H-NMR spectrum of compound 3 (400 MHz, C_6D_6, 298K).

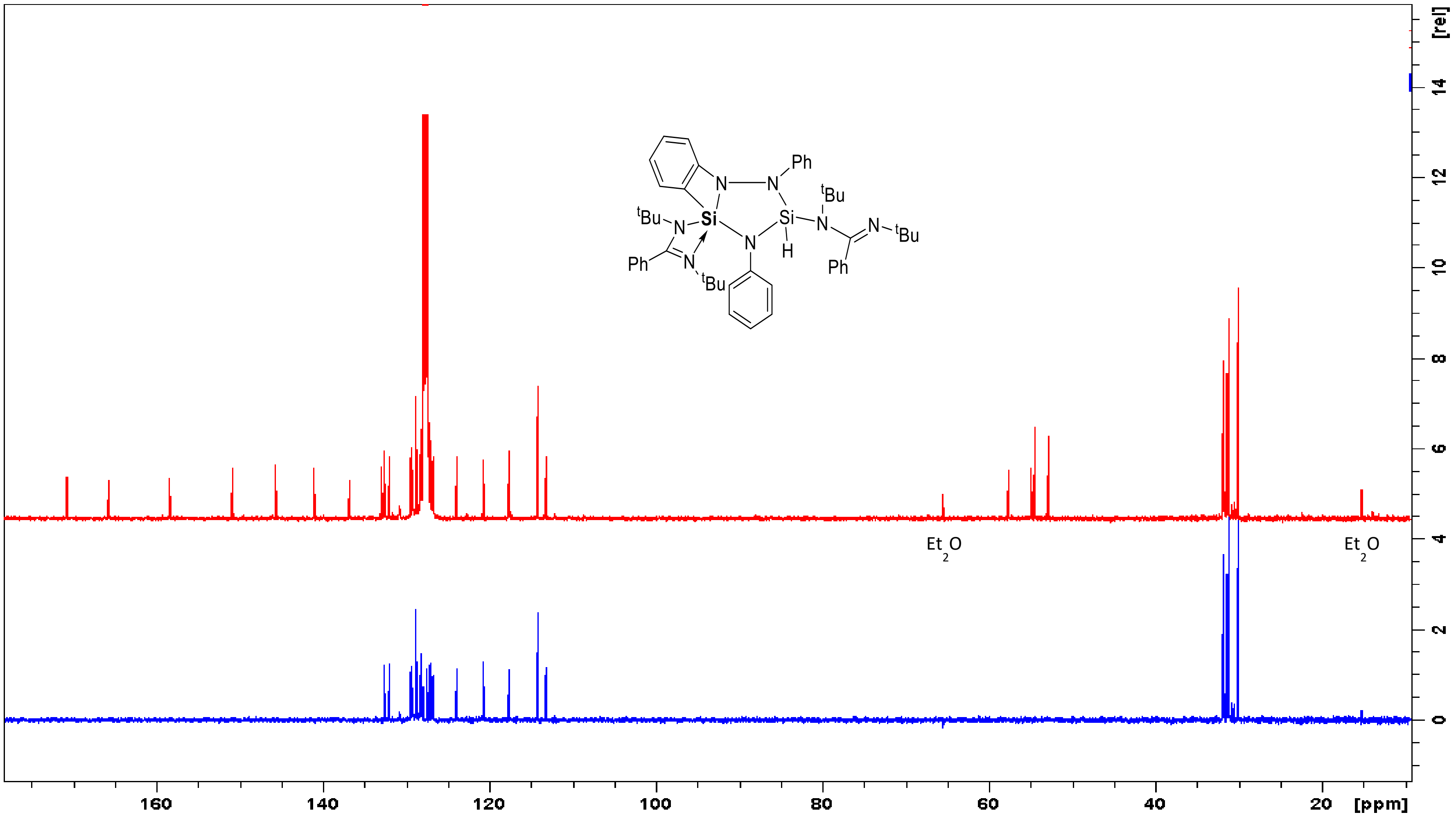

Figure S4. Comparison of $^{13}C\{^{1}H\}$- (top) and Dept-135 (bottom) NMR spectra of compound 3 (100 MHz, C_6D_6, 298 K).

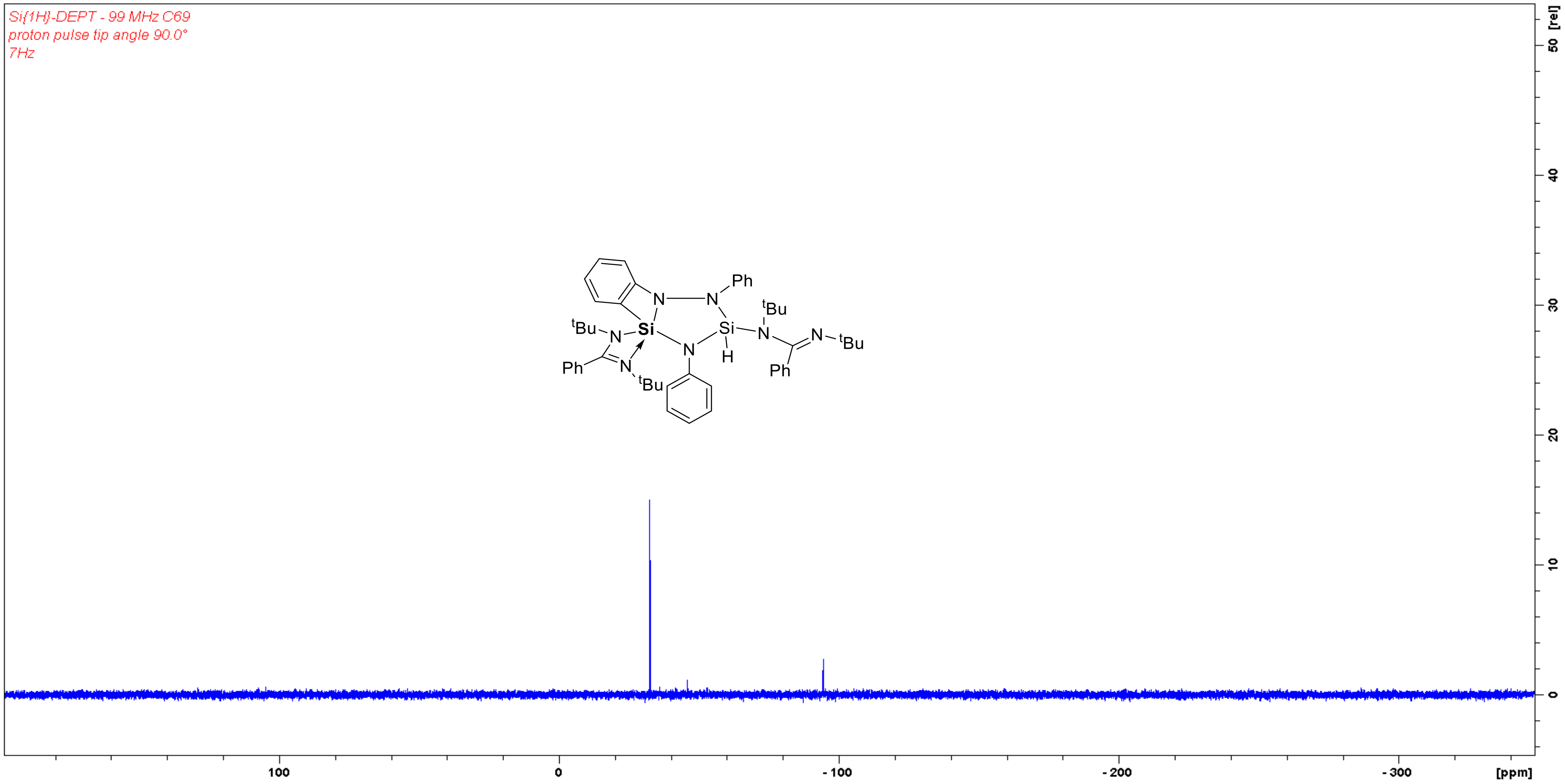

Figure S5. $^{29}Si\{^{1}H\}$-NMR spectrum of compound 3 (79 MHz, THF-D_8, 298 K).

Compound 4: To a Schlenk flask charged with yellow bis(silylene) PhN(LSi)$_2$ **(1)** (0.30 g, 0.49 mmol) and orange colored *cis*-azobenzene (0.090 g, 0.49 mmol) was added 20 mL THF at room temperature with stirring. The color of the reaction mixture turned red immediately. After stirring at room temperature for 2 hours, the resulted red solution was concentrated under reduced pressure and cooled at 4 °C for 1 day to give compound **4** as orange-colored crystals (yield: 0.30 g, 0.38 mmol, 78%). **M.P.** 171 ºC (decomp.). **^{1}H NMR** (400 MHz, THF-d_8, 298K): δ = 1.11 (*s*, 36 H, C(C*H*$_3$)$_3$), 6.56 (*t*, $J_{(H, H)}$ = 6.4 Hz, 3 H, arom.-*H*), 7.04 – 7.12 (*m*, 12 H, arom.-*H*), 7.55-7.63 (*m*, 6 H, arom.-*H*), 7.65-7.67 ppm (*m*, 4 H, arom.-*H*). **^{13}C{^{1}H} NMR** (125 MHz, THF-d_8, 298K): δ = 32.42 (NC(*C*H$_3$)$_3$), 55.94 (N*C*(CH$_3$)$_3$), 117.12, 125.48, 129.45, 129.66, 130.36, 131.94, 133.17, 153.19, 177.13 (N*C*N) ppm. **^{29}Si{^{1}H} NMR** (99 MHz, THF-d_8, 298 K): δ = -82.74 ppm (*s*, *Si*). **HR-ESI-MS: m/z**: 792.46002 (*calc*. 792.45997 [M+H]$^+$, m/z); **IR** (cm^{-1}): 2970.17(*w*), 2867.22(*w*), 1591.59(*w*), 1574.35(*w*), 1552.57(*w*), 1483.07(*s*), 1444.35(*w*), 1365.13(*s*), 1272.44(*s*), 1197.32(*m*), 1161.32(*w*), 1102.12(*w*), 1067.20(*m*), 1041.91(*w*), 1029.83(*w*), 1016.06(*w*), 1007.73(*m*), 992.86(*m*), 959.73(*w*), 910.27(*m*), 882.83(*w*), 845.75 (*s*), 789.30(*m*), 753.88(*s*), 735.63(*m*), 694.24(*vs*), 663.68(*w*), 646.24(*w*), 634.08(*w*), 615.26(*w*), 599.46(*m*), 565,91(*w*).

.

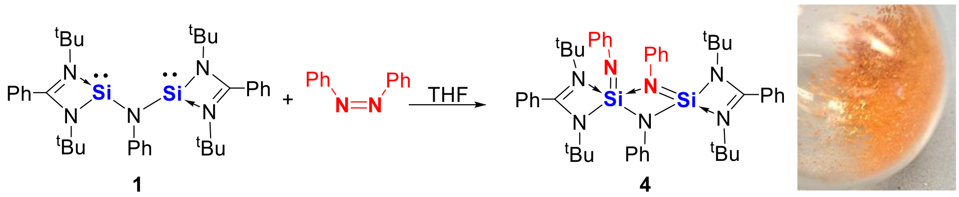

Scheme S3 Synthesis of **4**.

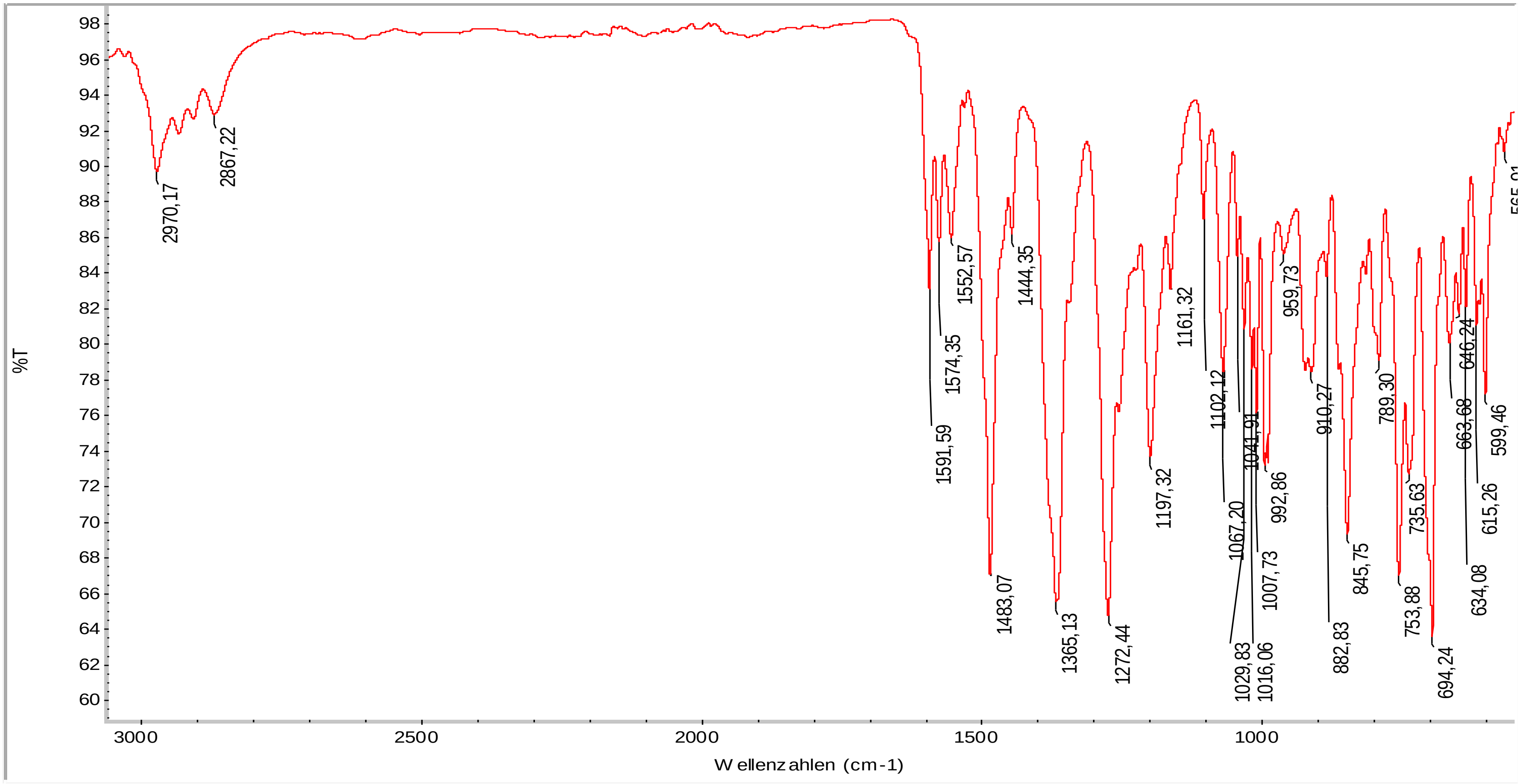

Figure S6. IR spectrum of compound 4.

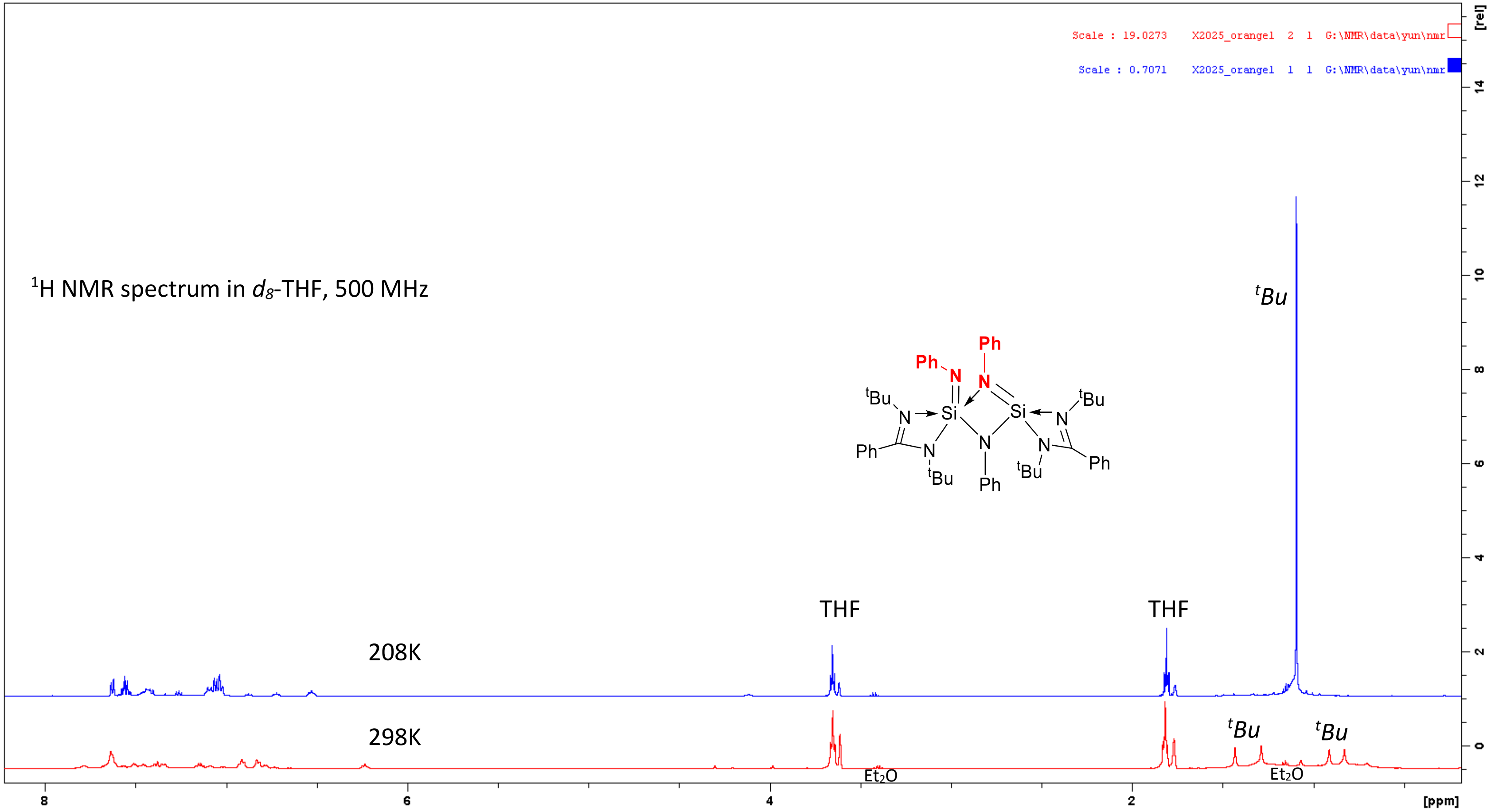

Figure S7. ^{1}H-NMR spectra of compound 4 (500 MHz, THF-d_8) at 298K (bottom) and at 208K (top).

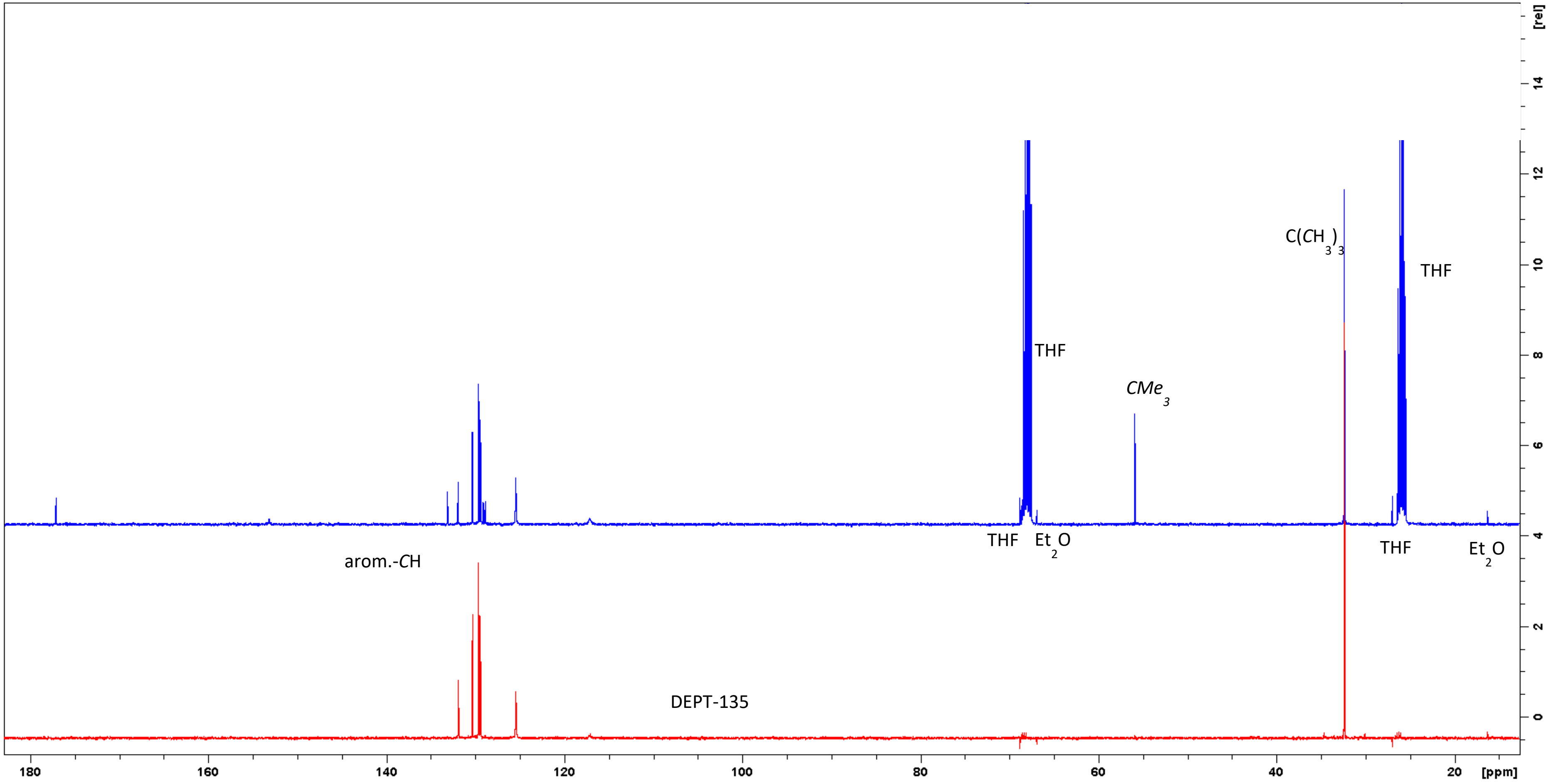

Figure S8. ^{13}C{^{1}H}- (top) and Dept-135 (bottom)-NMR spectra of compound 4 (100 MHz, THF-d_8, 298 K)

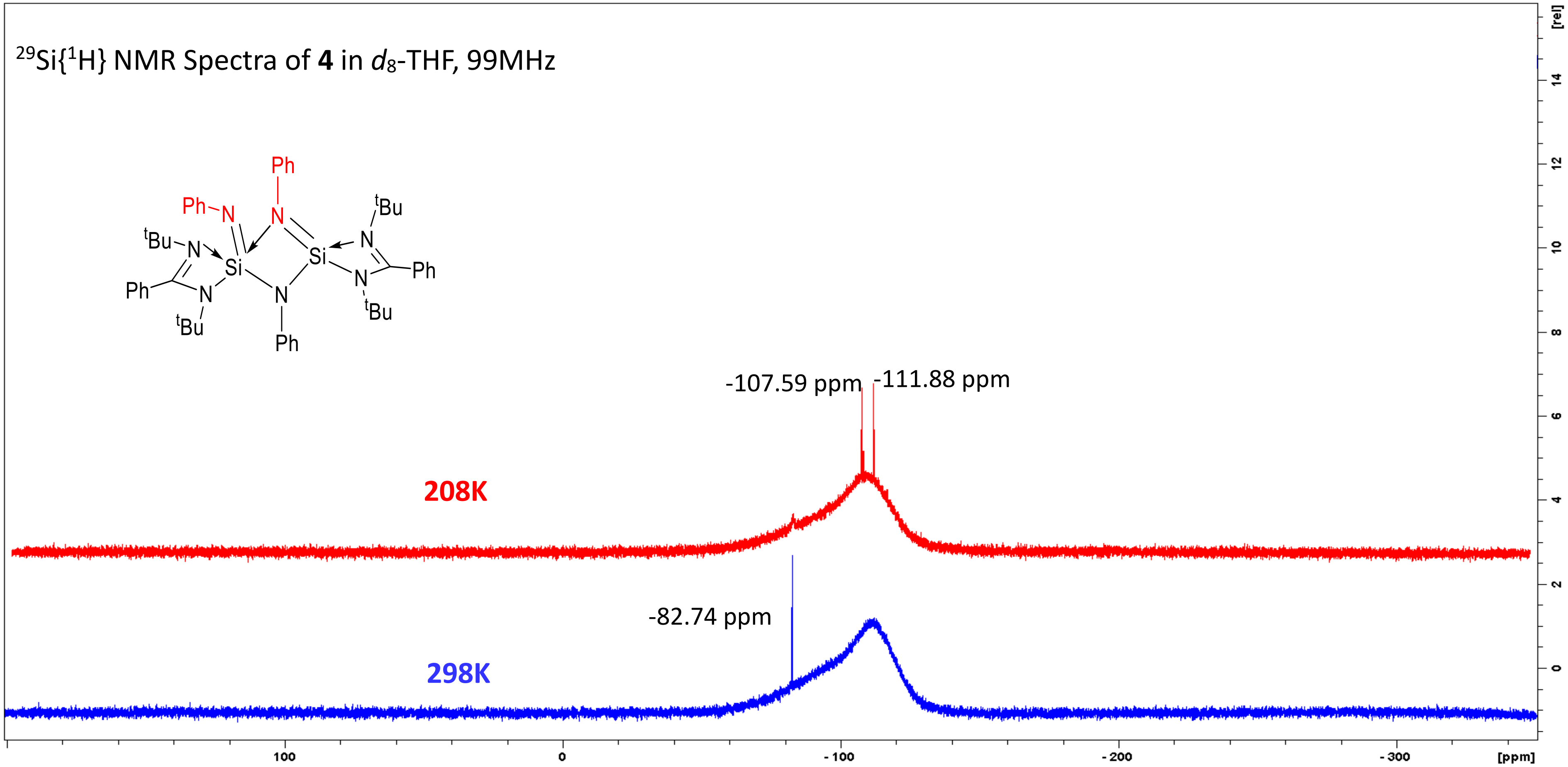

Figure S9a. $^{29}Si\{^1H\}$-NMR spectrum of compound 4 (79 MHz, THF-d_8, top: 208 K, bottom: 298K)

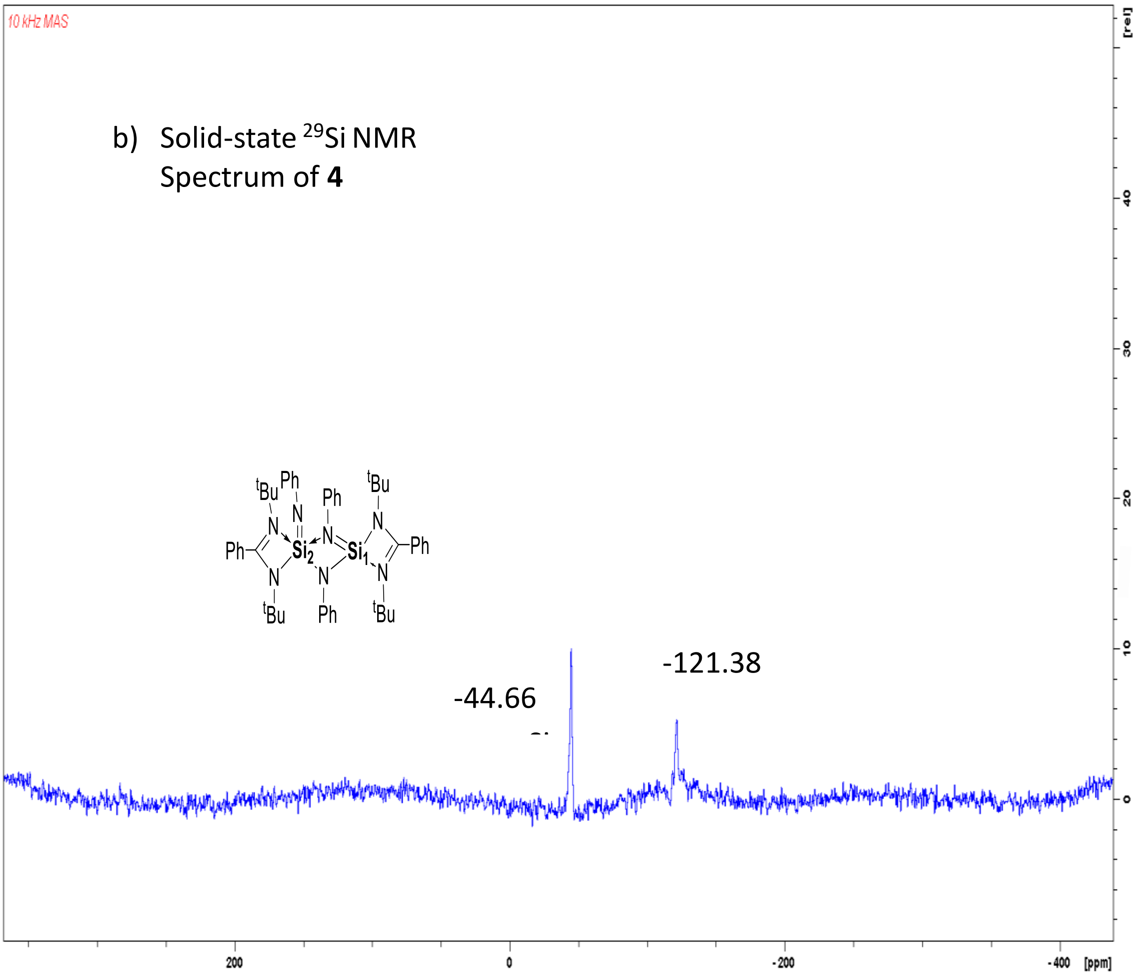

Figure S9b. ^{29}Si MAS-NMR spectrum of compound 4.

Compound 5: To a Schlenk flask charged with the bis(silylene) XT(LSi)$_2$ (**2**) (0.35 g, 0.48 mmol) and trans-azobenzene (0.087 g, 0.48 mmol) was added 15 mL THF at -20 °C with stirring. The reaction solution quickly turned colorless right away. After stirring at room temperature for 2 hours, the resulted solution was concentrated under reduced pressure and cooled at -20 °C for 1 day to give compound **5** as colorless crystals (yield: 0.30 g, 0.33 mmol, 69 %). **M.p**. 155 °C (decomp.). **^{1}H NMR** (400 MHz, C_6D_6, 298K): δ = 1.09 (*s*, 9 H, C(C*H*$_3$)$_3$), 1.14 (*s*, 9 H, C(C*H*$_3$)$_3$), 1.21 (*s*, 9 H, C(C*H*$_3$)$_3$), 1.37 (*s*, 3 H, *Me*), 1.44 (*s*, 3 H, *Me*), 1.48 (*s*, 9 H, C(C*H*$_3$)$_3$), 6.54 (*s*, 1 H, Si*H*, Si satellite: $^1J_{SiH}$ = 242.91 Hz), 6.79 (*t*, $^3J_{(HH)}$ = 7.28 Hz, 1 H, arom.-*H*), 7.09 – 7.29 (*m*, 12 H, arom.-*H*), 7.51 (*br*. 3 H, arom.-*H*), 7.67 (*t*, $^3J_{(HH)}$ = 7.55 Hz, 2 H, arom.-*H*), 7.74 (*d*, $^3J_{(HH)}$ = 6.87 Hz, 1 H, arom.-*H*), 7.87 (*d*, $^3J_{(HH)}$ = 8.93 Hz, 1 H, arom.-*H*), 7.91 (*d*, $^3J_{(HH)}$ = 5.50 Hz, 1 H, arom.-*H*), 8.00 – 8.03 (m, 2 H, arom.-*H*), 8.75 ppm (*d*, $^3J_{(HH)}$ = 6.67 Hz, 2 H, arom.-*H*). **^{13}C{^{1}H} NMR** (125 MHz, C_6D_6, 298K): δ = 31.72, 32.24, 32.64, 33.56 (NC(*C*H$_3$)$_3$), 34.26 (C(*C*H$_3$)$_2$), 34.47 (*C*(CH$_3$)$_2$), 54.01, 54.43, 54.64, 54.87 (N*C*(CH$_3$)$_3$), 114.00, 119.24, 119.66, 119.95, 121.80, 122.84, 122.99, 124.25, 125.93, 126.31, 126.80, 126.99, 127.37, 128.16, 128.83, 128.97, 129.61, 129.79, 129.89, 130.03, 130.69, 131.71, 132.63, 133.69, 134.34, 137.98, 140.31, 152.37, 153.95, 154.60, 161.62 (N*C*N), 163.96 ppm (N*C*N). **^{29}Si{^{1}H} NMR** (99 MHz, C_6D_6, 298 K): δ = -33.32 (*s*, Si), -38.38 ppm (*s*, *Si*H). **HR-ESI-MS: m/z**: 909.50641 (*calc*. 909.50659 [M+H]$^+$, m/z); **IR** (cm^{-1}): 2966.48(*w*), 2191.91(*w*, ν_{Si-H}), 1610.42(*w*), 1584.60(*m*), 1487.66(*m*), 1456.04(*w*), 1434.47(*m*), 1400.36(*s*),

1359.93(*m*), 1237.72(*s*), 1191.69(*s*), 1124.27(*w*), 1103.04(*m*), 1068.07(*w*), 1028.19(*w*), 1016.41(*w*), 945.17(*w*), 927.70(*w*), 893.05(*w*), 772.21(*vs*), 743.77(*vs*), 706.57(*s*), 693.02(*m*), 616.70(*w*), 589.43(*m*), 578.17(*w*), 566.97(*w*).

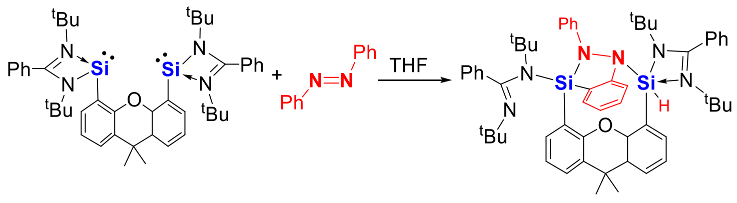

Scheme S4. Synthesis of **5**.

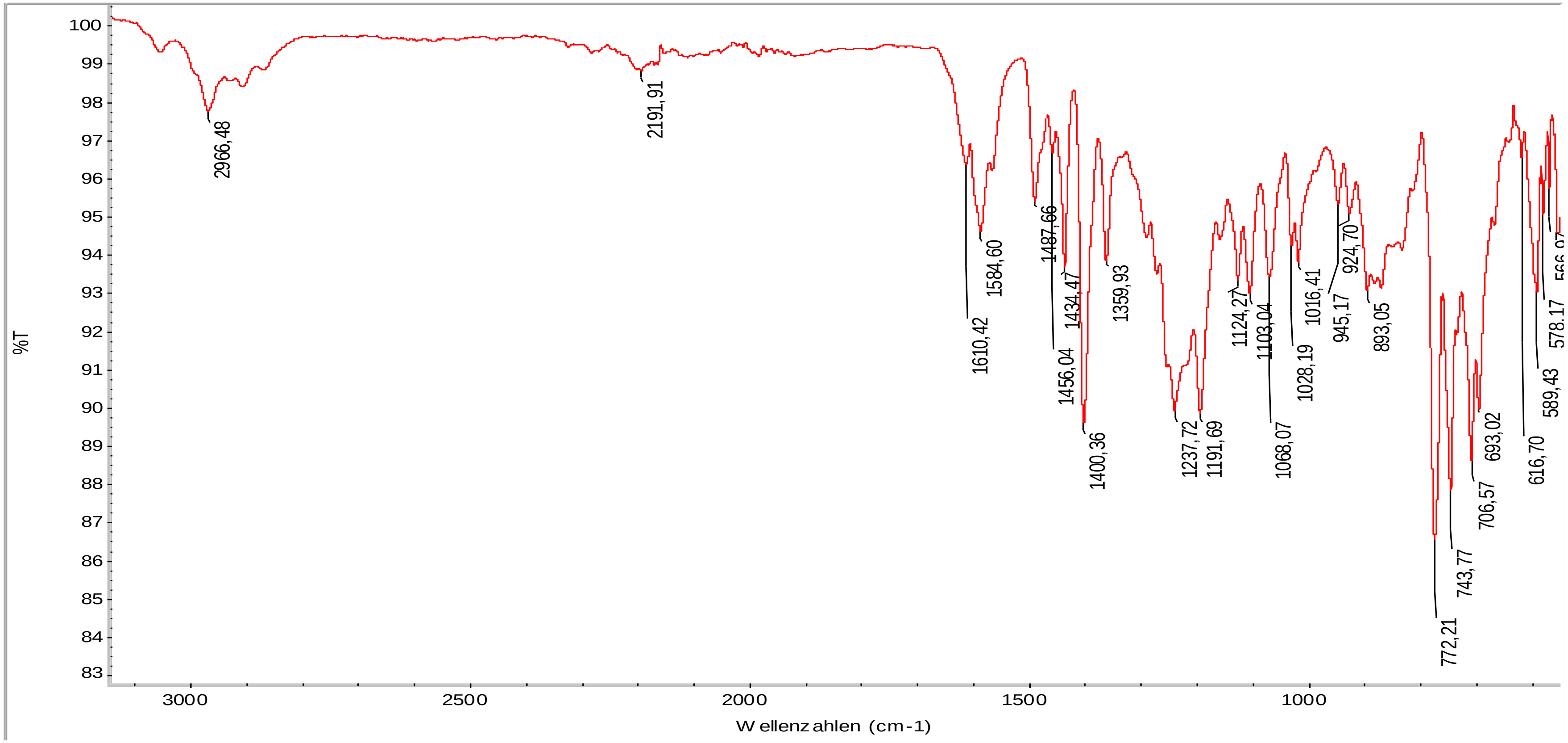

Figure S10. IR spectrum of compound 5.

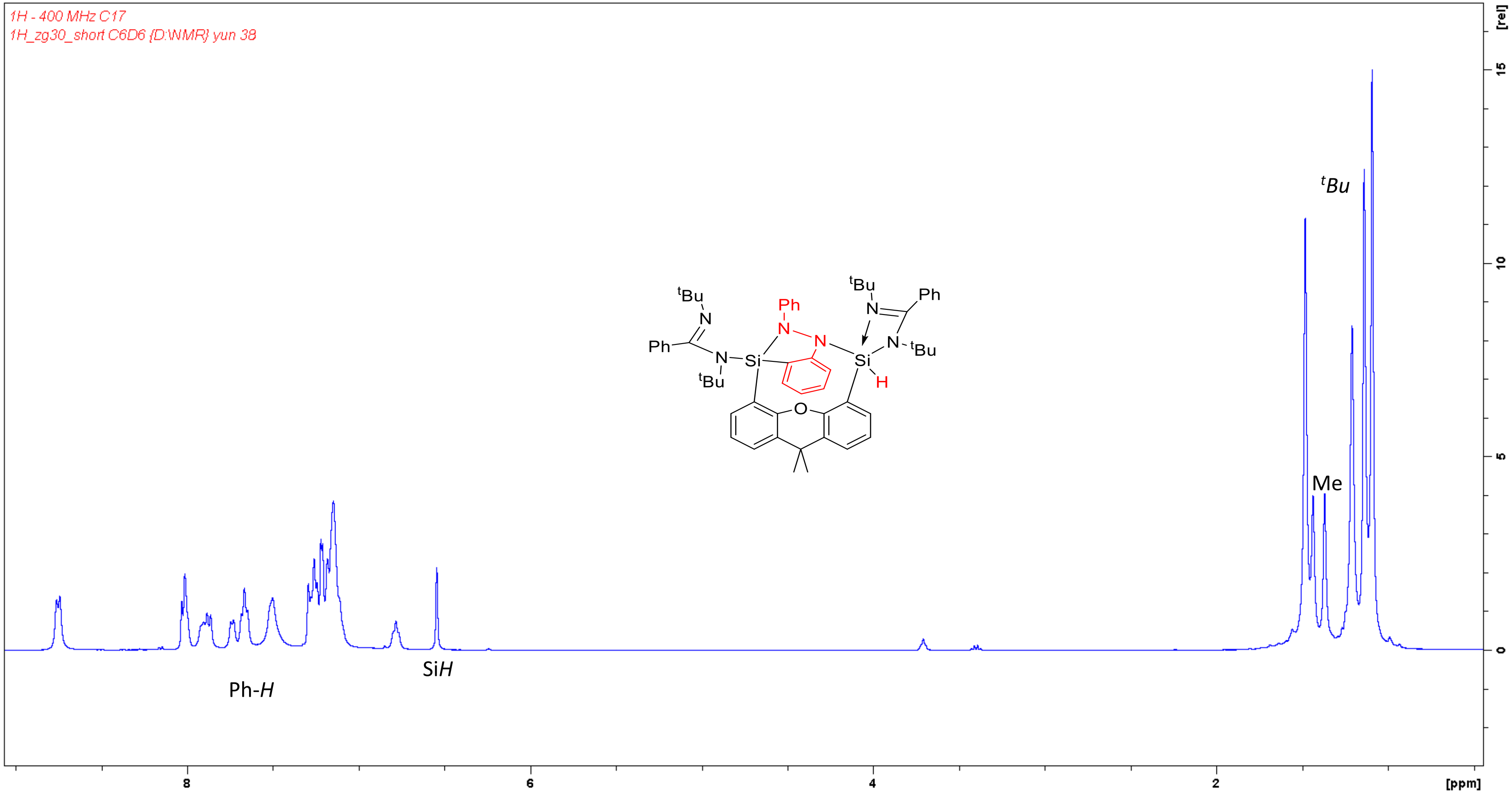

Figure S11. ^{1}H-NMR spectrum of compound 5 (400 MHz, C_6D_6, 298K).

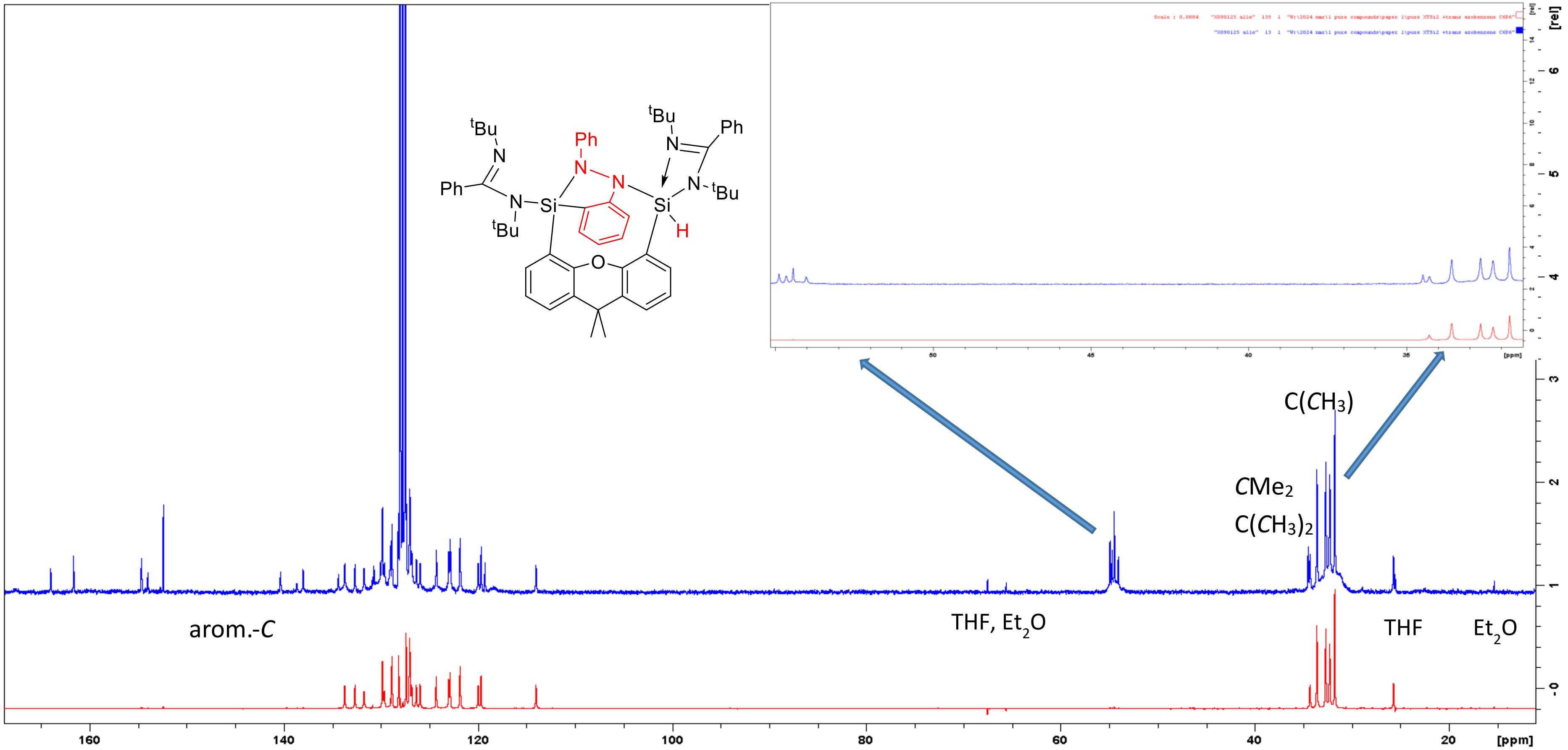

Figure S12. $^{13}C\{^{1}H\}$- (top) and Dept-135 (bottom) NMR spectra of compound 5 (100 MHz, C_6D_6, 298 K).

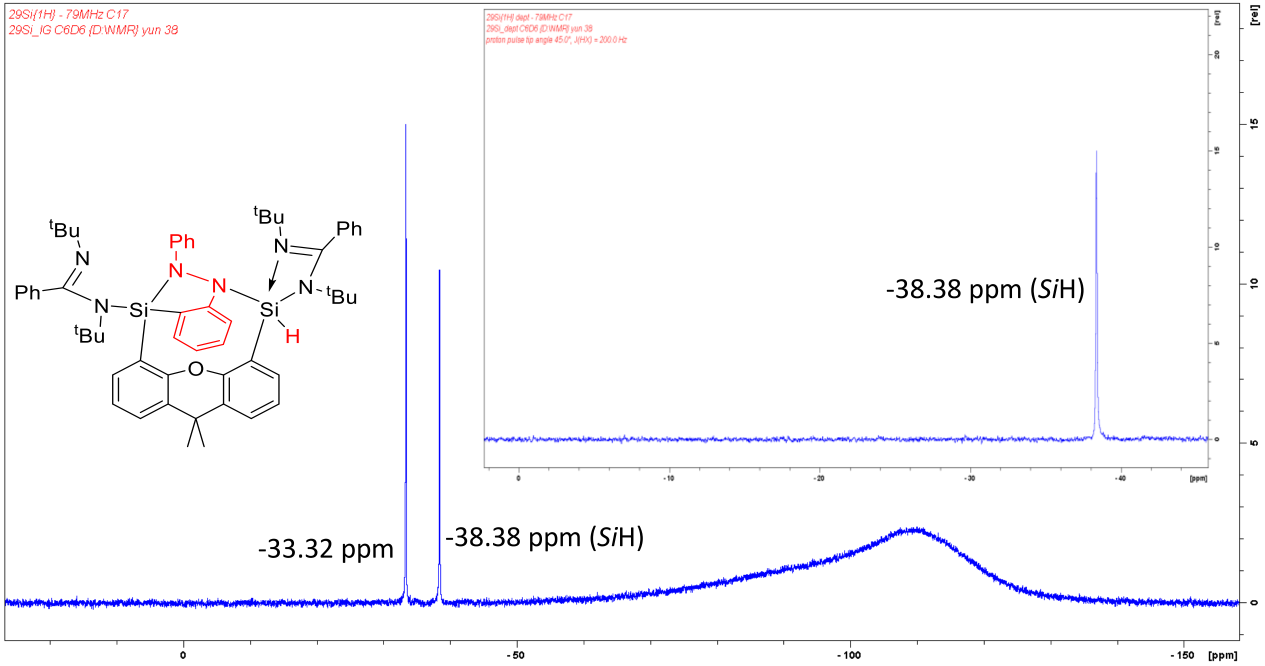

Figure S13. ^{29}Si{^{1}H}-NMR spectrum of **5** (bottom) and ^{29}Si{^{1}H}- DEPT NMR spectra of compound 5 (top) (79 MHz, C_6D_6, 298 K).

Compound 6: A solution of cis-azobenzene (0.13 g, 0.71 mmol) in THF was slowly added via a syringe to a solution of the bis(silylene) XT(LSi)$_2$ (**2**) (0.52, 0.71 mmol) in THF (25 ml) at -30 °C with stirring. The reaction mixture was stirred at that temperature for 20 min. The solvent THF was exchanged with *n*-pentane. The concentrated solution was stored at -20 °C. After one day yellowish crystals of **6** were formed in the yield of 0.44 g (0.48 mmol, 68%). **M.p.** 177 °C (decomp.). **^{1}H NMR** (400 MHz, C_6D_6, 298K): δ = 0.98 (*s*, 9 H, NC(C*H*$_3$)$_3$), 1.17 (*s*, 9 H, NC(C*H*$_3$)$_3$), 1.27 (*s*, 9 H, NC(C*H*$_3$)$_3$), 1.44 (*s*, 3 H, Me), 1.46 (*s*, 9 H, NC(C*H*$_3$)$_3$), 1.53 (*s*, 3 H, Me), 6.56 - 6.59 (*m*, 1 H, arom.-*H*), 6.67 – 6.71 (*m*, 1 H, arom.-*H*), 6.81-7.04 (*m*, 13 H, arom.-*H*), 7.16 – 7.26 (*m*, 4 H, arom.-*H*), 7.38 (*d*, $^3J_{(H,H)}$ = 7.37 Hz, 1 H, arom.-*H*), 7.42 (*t*, $^3J_{(H,H)}$ = 7.8 Hz, 2 H, arom.-*H*), 7.60 (*d*, $^3J_{(H,H)}$ = 7.17 Hz, 1 H, arom.-*H*), 7.82 (*d*, $^3J_{(H,H)}$ = 7.91 Hz, 2 H, arom.-*H*), 8.04 (*d*, $^3J_{(H,H)}$ = 7.50 Hz, 1 H, arom.-*H*). **^{13}C{^{1}H} NMR** (100 MHz, C_6D_6, 298K): δ = 31.37 (NC(*C*H$_3$)$_3$), 31.56 (NC(*C*H$_3$)$_3$), 31.68 (NC(*C*H$_3$)$_3$), 31.87 (NC(*C*H$_3$)$_3$), 33.21 (C(*C*H$_3$)$_2$), 34.31(*C*Me$_2$), 52.60 (N*C*Me$_3$), 52.82 (N*C*Me$_3$), 54.20 (N*C*Me$_3$), 54.79 (N*C*Me$_3$), 115.96, 116.57, 117.03, 117.43, 121.65, 121.89, 123.16, 126.50, 127.08, 127.36, 127.41, 127.44, 127.58, 127.73, 127.77, 127.99, 128.51, 128.66, 128.80, 128.86, 129.53, 129.75, 130.20, 130.43, 130.65, 132.38, 133.61, 135.18, 153.54, 154.15, 155.12, 156.62, 157.92 (N*C*N), 177.91 (N*C*N). **^{29}Si{^{1}H} NMR** (79 MHz, C_6D_6, 298 K): δ = 14.83 (*s*, *Si* atom in silylene moiety), -115.84 ppm (*s*, *Si*N$_4$). **HR-ESI-MS: m/z**: 909.50745 (calc. 909.50659 [M+H]$^+$); **IR** (cm^{-1}): 2970.77(*w*), 1589.37(*w*), 1532.67(*w*), 1482.09(*m*), 1443.97(*w*), 1388.02(*vs*), 1361.25(*m*), 1269.83(*w*), 1225.89(*s*), 1206.55(*s*), 1163.39(*w*), 1117.52(*w*), 1088.49(*m*), 1072.00(*m*), 1022.88(*m*), 995.93(*m*), 976.39(*w*), 929.13(*w*), 879.97(*m*), 798.14(*m*), 773.61(*s*), 748.18(*vs*), 707.60(*s*), 691.63(*m*), 661.14(*w*), 613.67(*s*), 572.74(*w*).

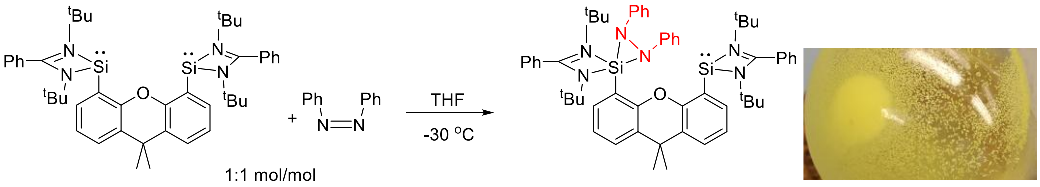

2 **6**

Scheme S5 Synthesis of **6**.

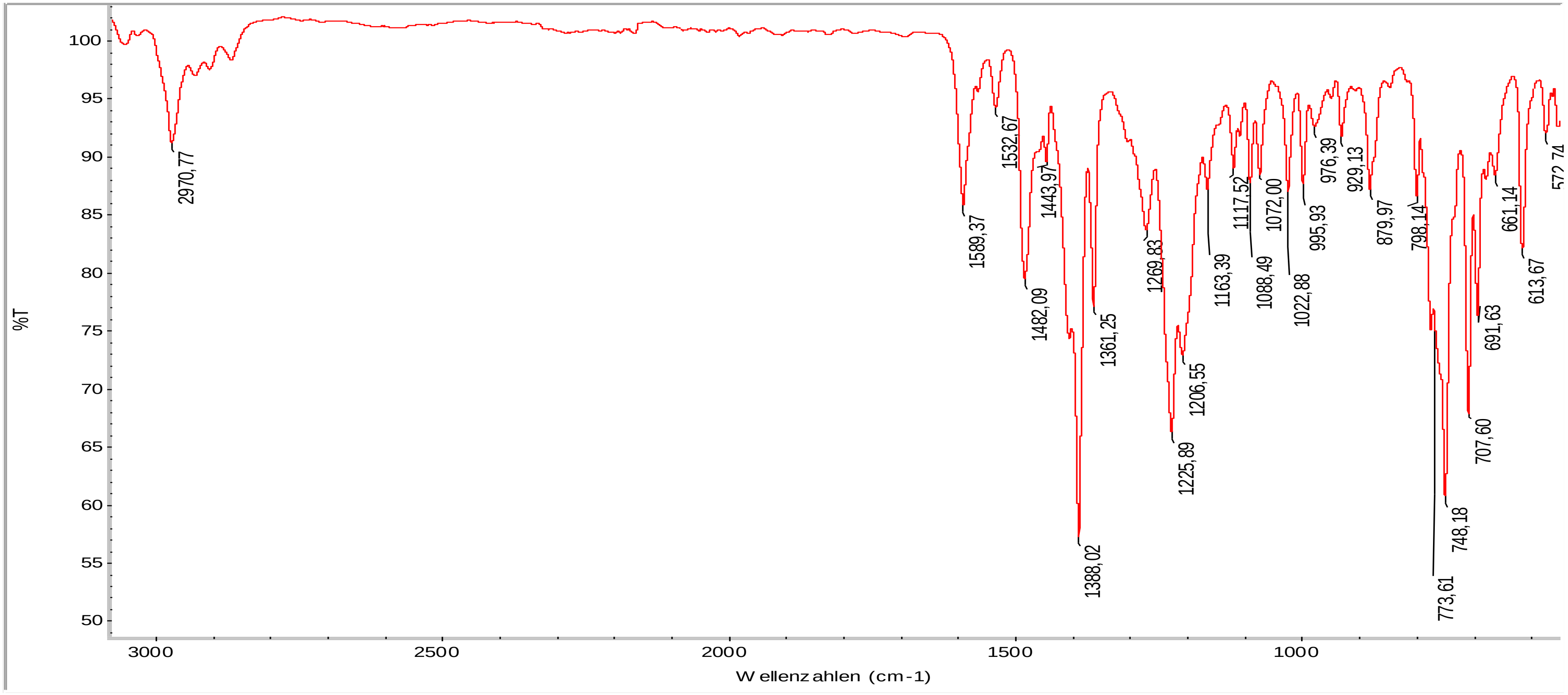

Figure S14. IR spectrum of compound **6**.

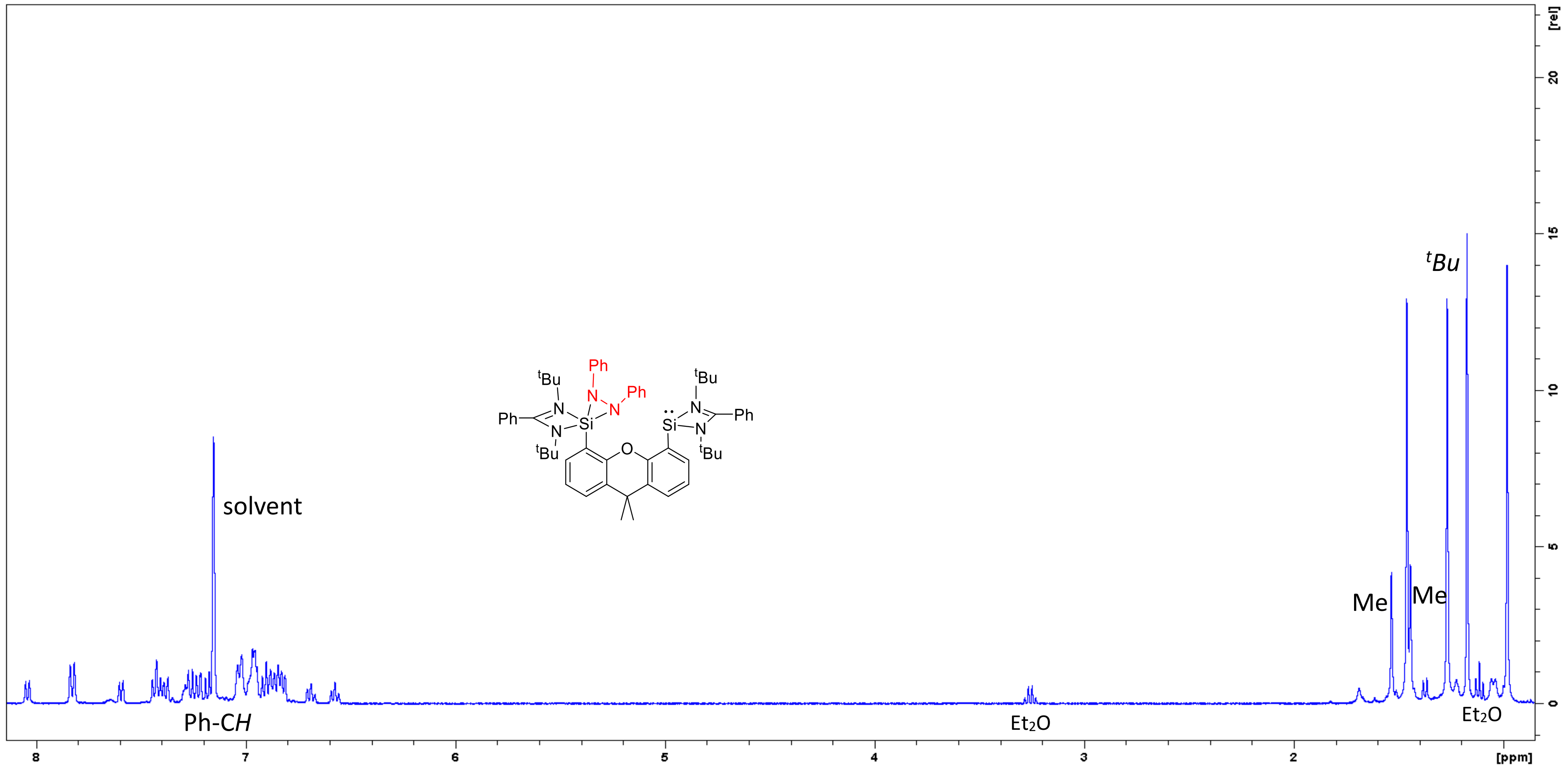

Figure S15. ^{1}H-NMR spectrum of compound 6 (400 MHz, C_6D_6, 298K).

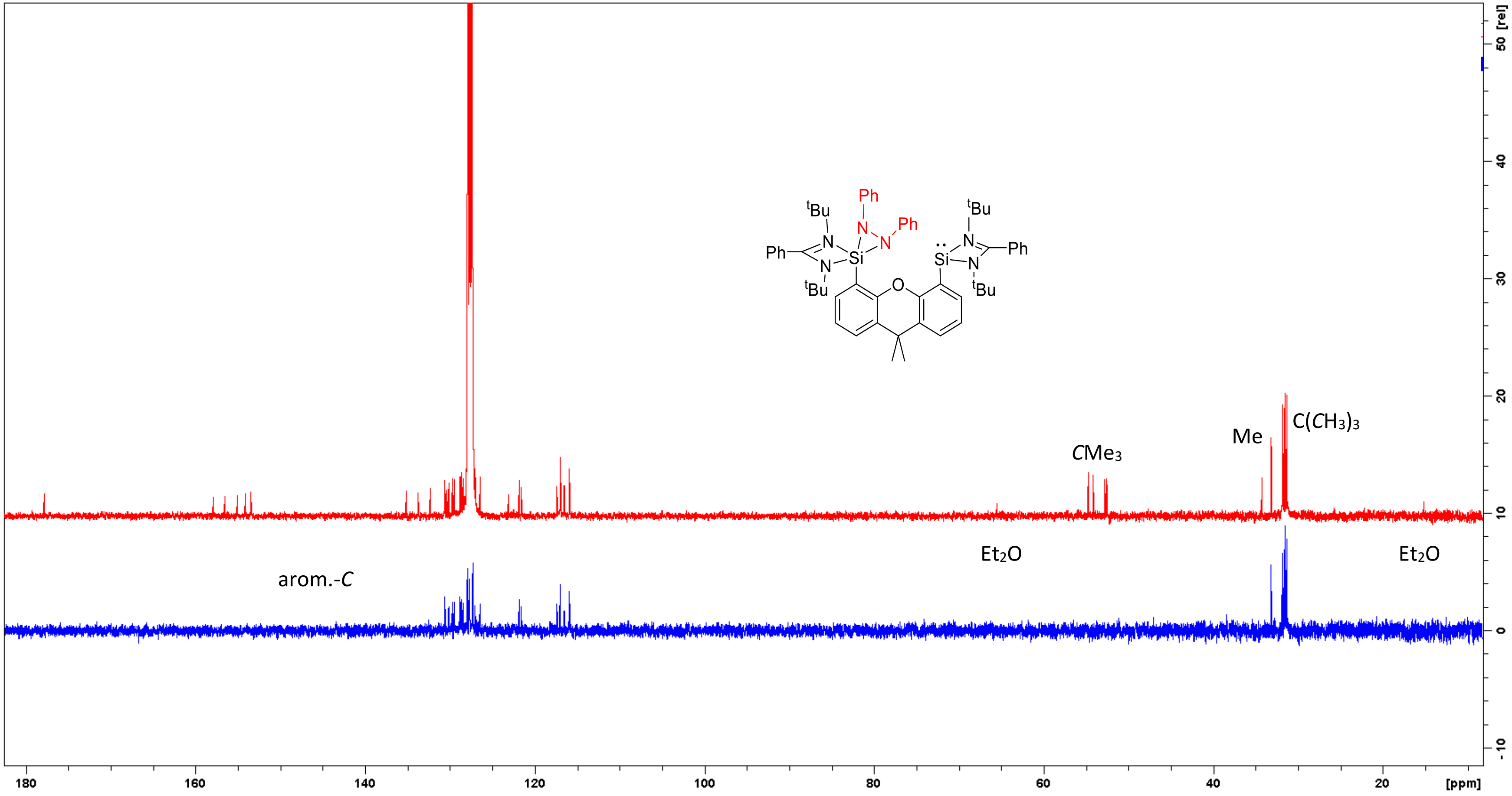

Figure S16. Comparison of the $^{13}C\{^{1}H\}$- (top) and Dept-135 (bottom) NMR spectra of compound **6** (100 MHz, C_6D_6, 298 K).

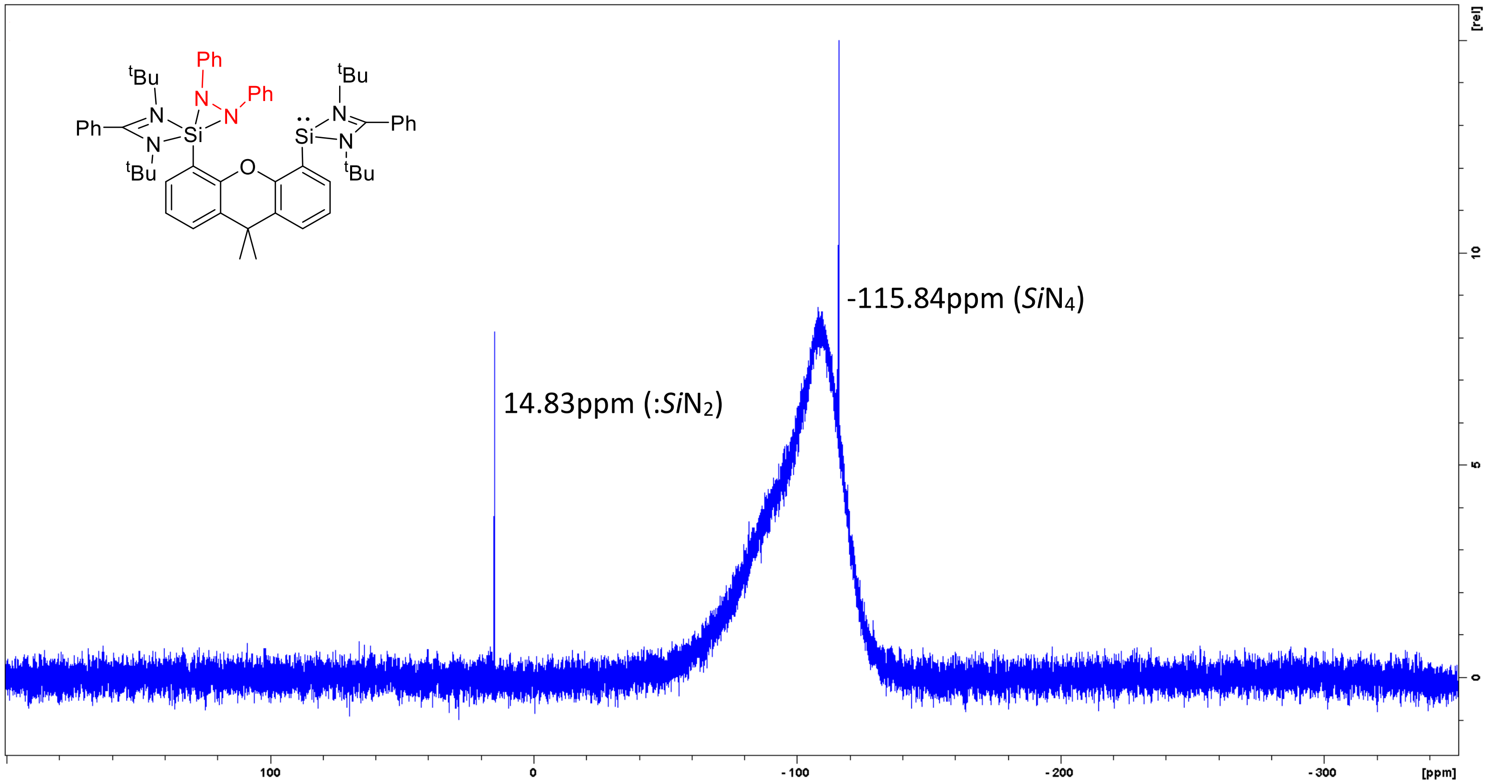

Figure S17. $^{29}Si\{^{1}H\}$-NMR spectrum of compound 6 (79 MHz, C_6D_6, 298 K).

Compound 7: Method I: A red solution of cis-azobenzene (0.24 g, 1.32 mmol) in THF (15 mL) was added via a syringe to a yellow solution of the bis(silylene) XT(LSi)$_2$ (**2**) (0.48 g, 0.66 mmol) in THF (25 ml) at room temperature with stirring. The color of the reaction mixture vanished after the complete reaction. The solvent THF was exchanged with Et_2O. From the concentrated Et_2O solution at -20 °C colorless crystals of **7** were formed with a yield of 0.59 g (0.54 mmol, 82 %). Method II: To an NMR tube charged with **5** (0.020 g, 0.022 mmol) and *cis*-azobenzene (0.0040 g, 0.022 mmol) was added 0.5 ml THF-d_8 at room temperature. The proton NMR showed that compound **5** could completely converted to compound **7**. **M.P**. 244 °C (decomp.). **^{1}H NMR** (400 MHz, THF-d_8, 298K): δ = 1.02 (*s*, 18 H, NC(C*H*$_3$)$_3$), 1.44 (*s*, 6 H, C(C*H*$_3$)$_2$), 1.46 (*s*, 18 H, NC(C*H*$_3$)$_3$), 6.12 (*t*, $^3J_{(HH)}$ = 6.47 Hz, 2 H, arom.-*H*), 6.31 (*d*, $^3J_{(HH)}$ = 7.55 Hz, 4 H, arom.-*H*), 6.54 (t, $^3J_{(HH)}$ = 7.46 Hz, 4 H, arom.-*H*), 6.76 (*t*, $^3J_{(HH)}$ = 6.77 Hz, 2 H, arom.-*H*), 6.89 (*d*, $^3J_{(HH)}$ = 7.73 Hz, 2 H, arom.-*H*), 6.97 (*t*, $^3J_{(HH)}$ = 7.41 Hz, 2 H, arom.-*H*), 7.10 – 7.17 (*m*, 10 H, arom.-*H*), 7.42 – 7.46 (*m*, 2 H, arom.-*H*), 7.54 – 7.60 (*m*, 4 H, arom.-*H*), 7.68 (*d*, $^3J_{(HH)}$ = 6.93 Hz, 2 H, arom.-*H*), 7.73 ppm (*d*, $^3J_{(HH)}$ = 6.50 Hz, 2 H, arom.-*H*). **^{13}C{^{1}H} NMR** (100 MHz, THF-d_8, 298K): δ = 30.87 (NC(*C*H$_3$)$_3$), 31.77 (NC(*C*H$_3$)$_3$), 34.27 (*C*Me$_2$), 34.30 (C(*C*H$_3$)$_2$), 54.08 (N*C*Me$_3$), 54.16 (N*C*Me$_3$), 113.91, 114.75, 116.73, 117.81, 118.90, 120.67, 127.30, 127.42, 127.58, 127.89, 128.02, 128.64, 129.57, 130.04, 131.94, 132.20, 153.11, 153.28, 154.70 (N*C*N), 176.83 ppm (N*C*N). **^{29}Si{^{1}H} NMR** (79 MHz, THF-d_8, 298 K): δ = -115.28 ppm (*s*, *Si*N$_4$). **HR-ESI-MS: m/z**: 1091.59387 (calc. 1091.59099 [M+H]$^+$); **IR** (cm^{-1}): 2972.99(*w*), 2866.67(*w*), 1590.33(*m*), 1531.62(*w*), 1485.07(*s*), 1446.54(*w*), 1402.60(*s*), 1386.52(*vs*), 1364.24(*w*), 1304.89(*w*), 1269.99(*m*), 1217.39(*vs*), 1164.99(*m*), 1110.01(*m*), 1089.66(*m*), 1074.80(*w*), 1025.26(*m*), 994.65(*m*), 965.88(*m*), 932.22(*w*), 876.61(*m*), 867.79(*m*), 800.21(*m*), 787.42(*w*), 772.92(*s*), 757.54(*s*), 743.18(*vs*), 734.58(*s*), 714.30(*s*), 691.32(*s*), 673.73(*s*), 616.34(*s*), 574.74(*m*).

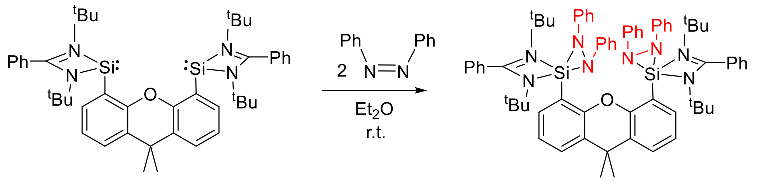

2 **7**

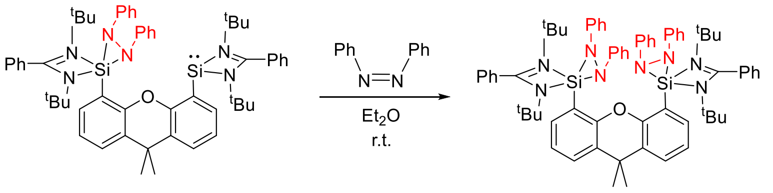

6 **7**

Scheme S6. Synthesis of **7**.

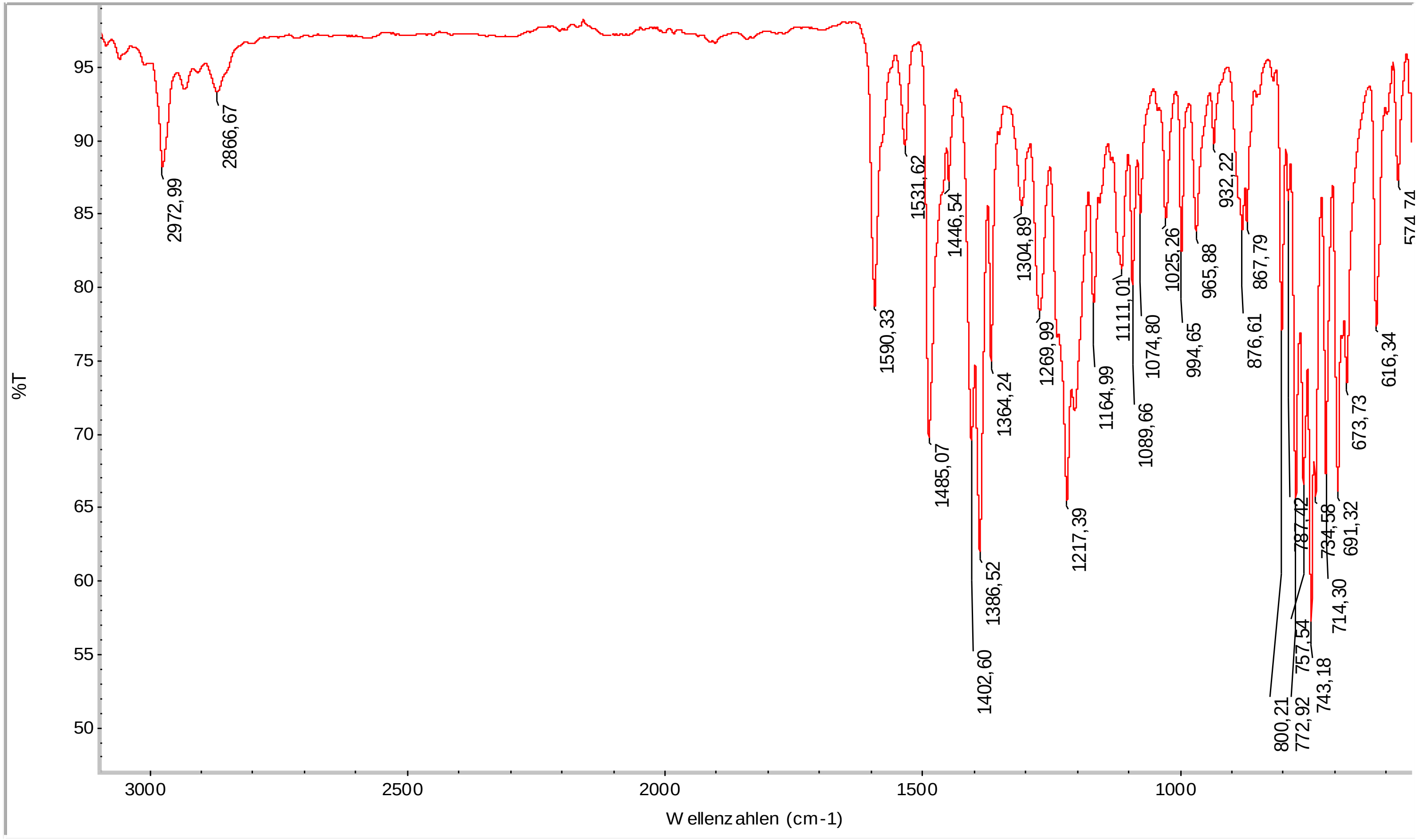

Figure S18. IR spectrum of compound **7**.

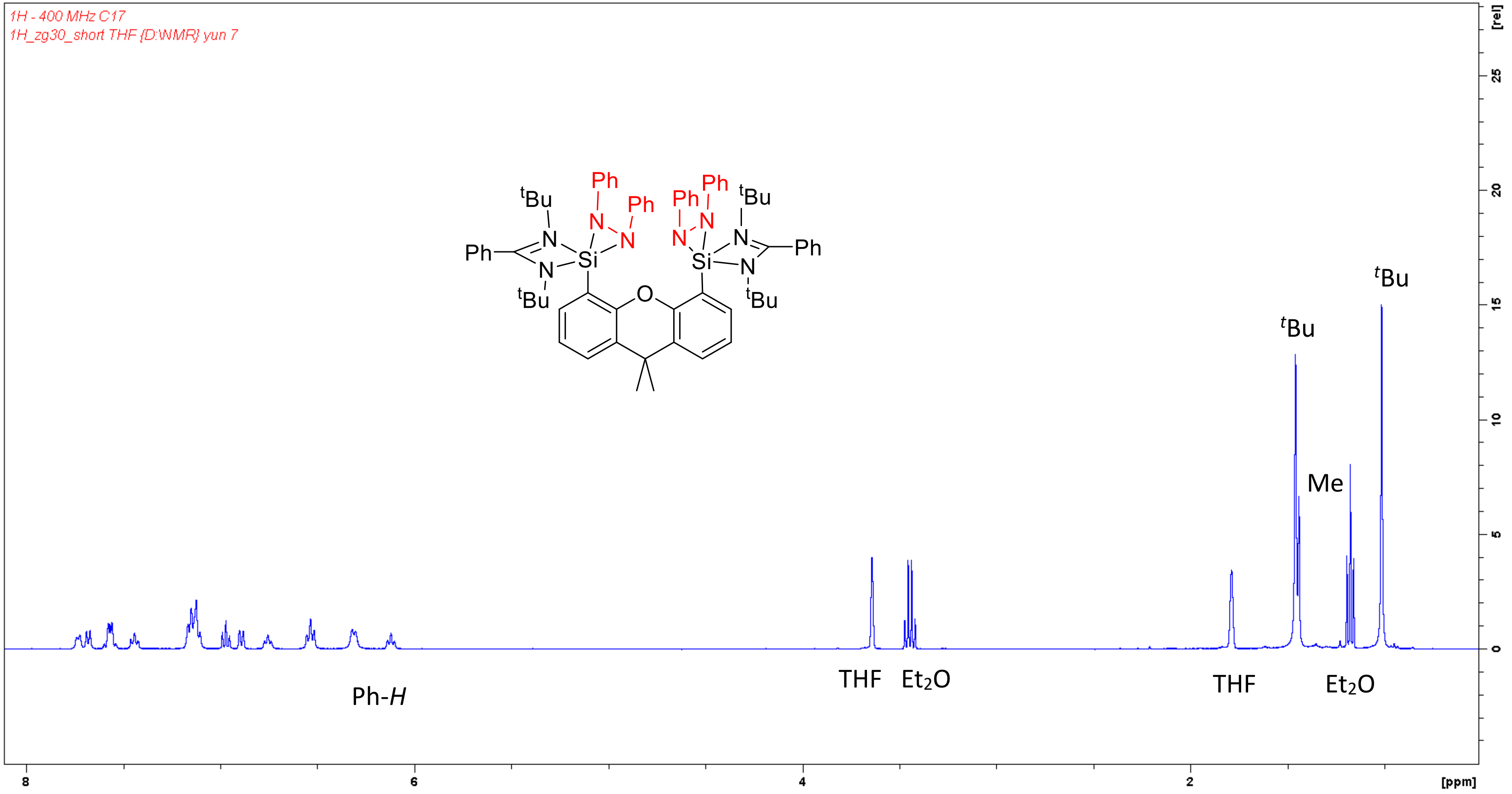

Figure S19. ^{1}H-NMR spectrum of compound **7** (400 MHz, THF-d_8, 298K).

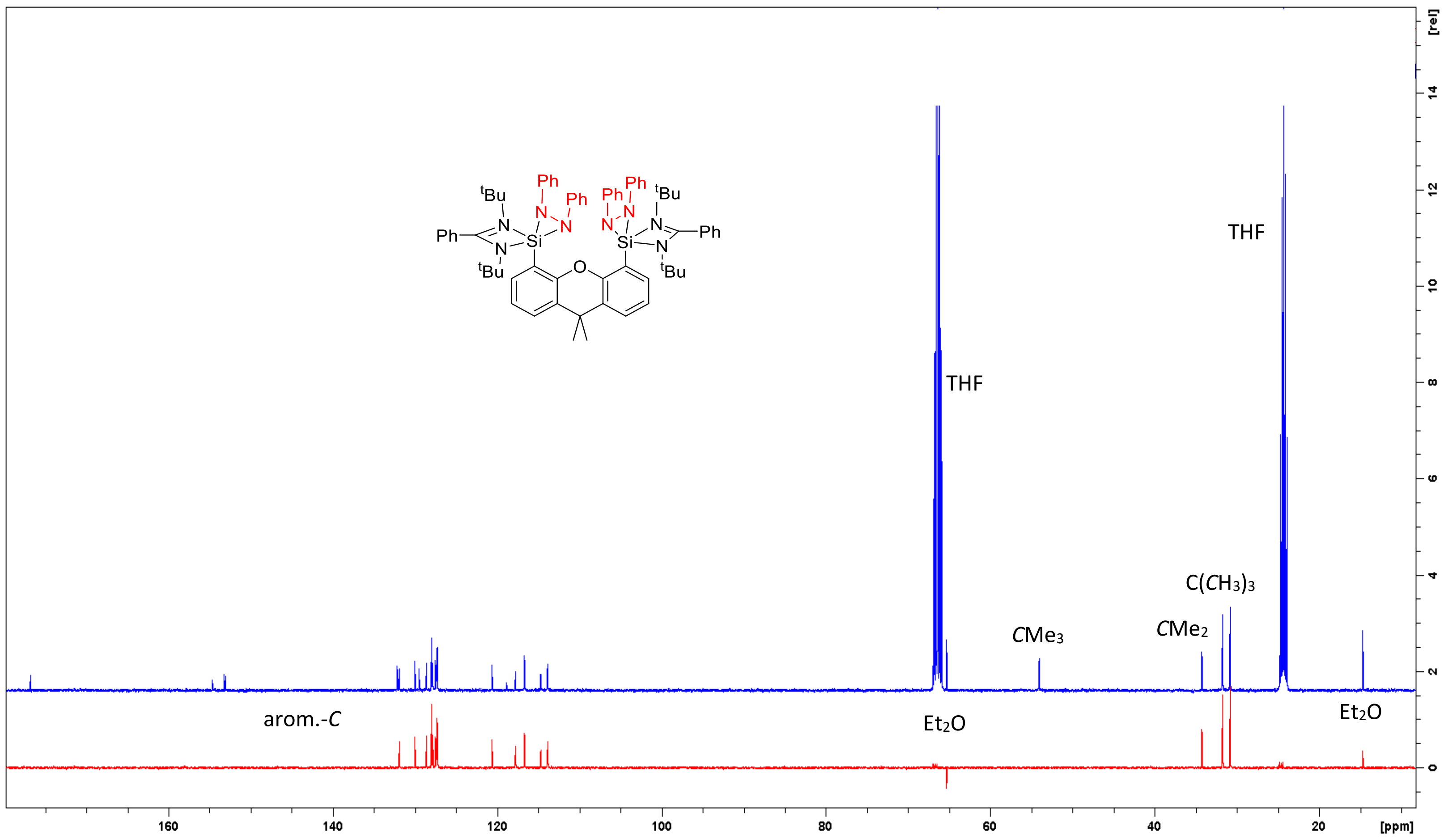

Figure S20. $^{13}C\{^{1}H\}$- (up) and Dept-135 (bottom) NMR spectra of compound **7** (100 MHz, THF-d_8, 298 K).

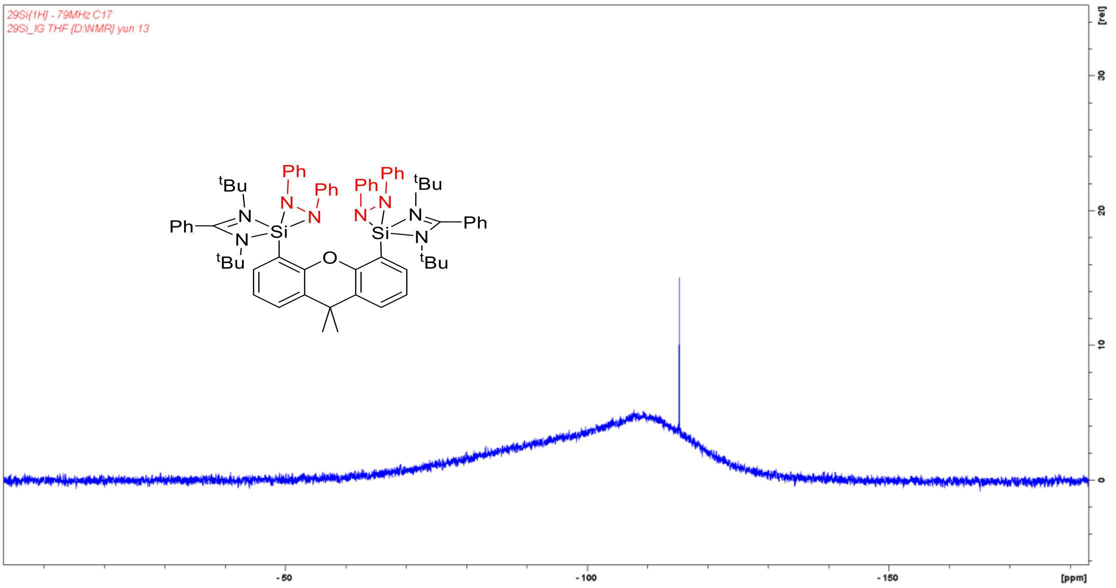

Figure S21. ^{29}Si{^{1}H}-NMR spectrum of compound **7** (79 MHz, THF-d_8, 298 K).

Compound 8: The yellow powder of **6** (0.30 g, 0.33 mmol) was dissolved in *n*-pentane (8 mL) at room temperature and stored at that temperature. After several days, the yellow color disappeared, and colorless crystals of **8** were formed. These crystals were separated from the solution by filtration and dried under reduced pressure (yield: 0.26 g, 0.28 mmol, 85 %). **M.P.** 332 °C (decomp.). **^{1}H NMR** (400 MHz, C_6D_6, 298K): δ = 1.05 (*s*, 18 H, NC(C*H*$_3$)$_3$), 1.36 (*s*, 18 H, NC(C*H*$_3$)$_3$), 1.48 (*s*, 3 H, C(C*H*$_3$)$_2$), 1.66 (*s*, 3 H, C(C*H*$_3$)$_2$), 6.32 (*d*, $^{3}J_{(HH)}$ = 7.5 Hz, 2 H, arom.-*H*), 6.40 (*t*, $^{3}J_{(HH)}$ = 6.8 Hz, 1 H, arom.-*H*), 6.82 (*t*, $^{3}J_{(HH)}$ = 7.6 Hz, 2 H, arom.-*H*), 6.97 (*br*. 7 H, arom.-*H*), 7.10 (*d*, $^{3}J_{(HH)}$ = 8.0 Hz, 3 H, arom.-*H*), 7.16 – 7.23 (*m*, 5 H, arom.-*H*), 7.45 (*t*, $^{3}J_{(HH)}$ = 7.5 Hz, 2 H, arom.-*H*), 7.84 (*d*, $^{3}J_{(HH)}$ = 7.37 Hz, 2 H, arom.-*H*), 8.07 ppm (*d*, $^{3}J_{(HH)}$ = 8.2 Hz, 2 H, arom.-*H*). **^{13}C{^{1}H} NMR** (100 MHz, C_6D_6, 298K): δ = 22.04 (*s*, C(*C*H$_3$)$_2$), 29.43 (*s*, C(*C*H$_3$)$_2$), 30.90 (*br*, NC(*C*H$_3$)$_3$), 32.98 (*br*, NC(*C*H$_3$)$_3$), 37.96 (s, *C*Me$_2$), 54.26 (*br*, N*C*Me$_3$), 115.97, 121.13, 121.67, 122.19, 122.56, 126.60, 126.91, 127.29, 127.99, 128.97, 129.74, 129.97, 130.75, 136.12, 136.94, 148.74, 148.79, 160.10 (N*C*N), 167.61 ppm (s, N*C*N). **^{29}Si{^{1}H} NMR** (79 MHz, C_6D_6, 298 K): δ = -81.59 ppm (s, Si). **HR-ESI-MS: m/z**: 909.50647 (calc. 909.50659 [M+H]$^{+}$); **IR** (cm^{-1}): 2971.98(*w*), 2931.47(*w*), 1590.87(*m*), 1565.87(*s*), 1484.40(*s*), 1444.83(*w*), 1428.87(*w*), 1394.51(*s*), 1360.09(*s*), 1318.58(*w*), 1258.19(*s*), 1222.52(*s*), 1194.61(*s*), 1142.72(*w*), 1117.73(*w*), 1073.44(*m*), 1028.19(*w*), 1019.03(*w*), 1004.62(*w*), 995.52(*m*), 961.29(*w*), 908.47(*vs*), 880.98(*w*), 839.55(*s*), 768.25(*s*), 754.39(*m*), 731.37(*m*), 706.74(*m*), 697.08(*vs*), 679.64(*w*), 654.61(*m*), 615.01(*m*), 592.57(*w*), 576.14(*w*), 557.07(*w*).

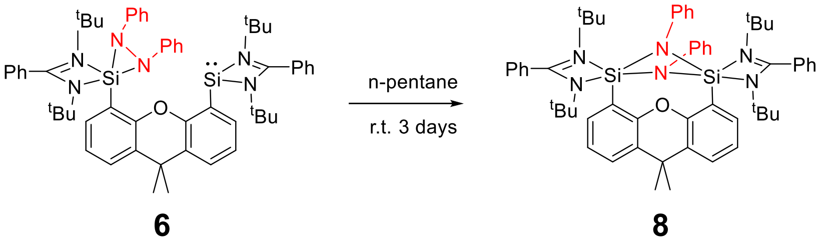

Scheme S7. Synthesis of **8**.

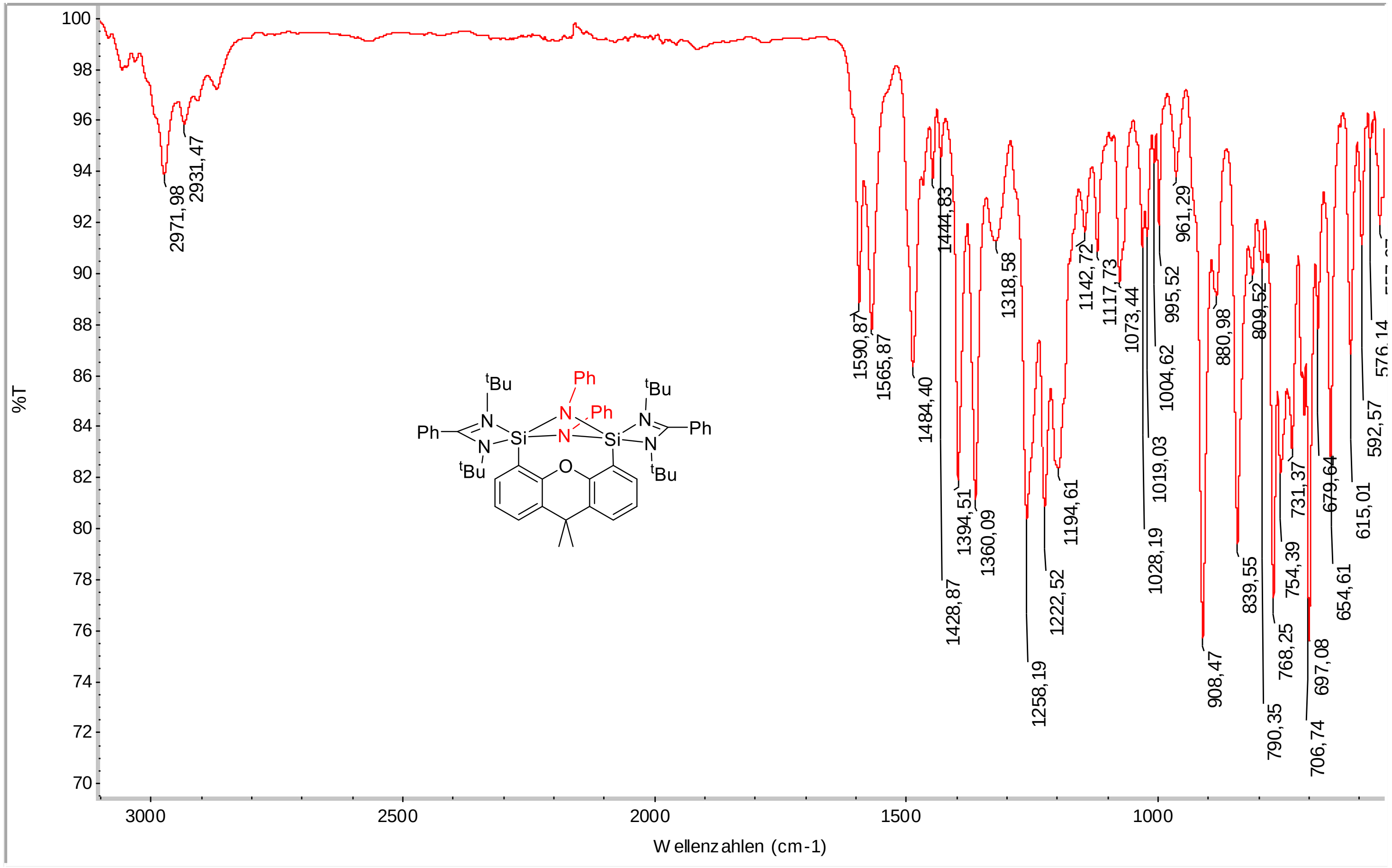

Figure S22. IR spectrum of compound 8.

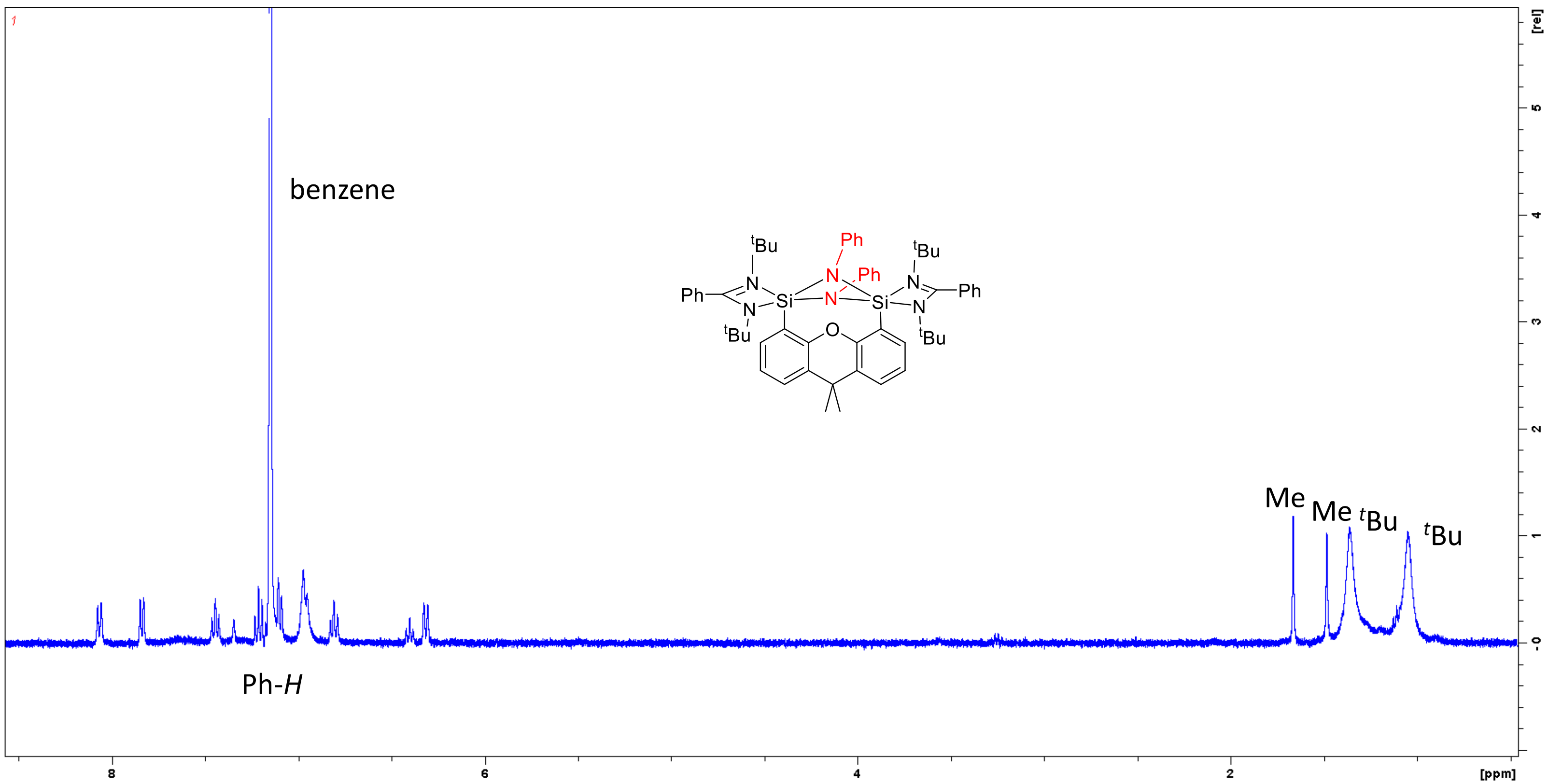

Figure S23. ^{1}H-NMR spectrum of compound 8 (400 MHz, C_6D_6, 298K).

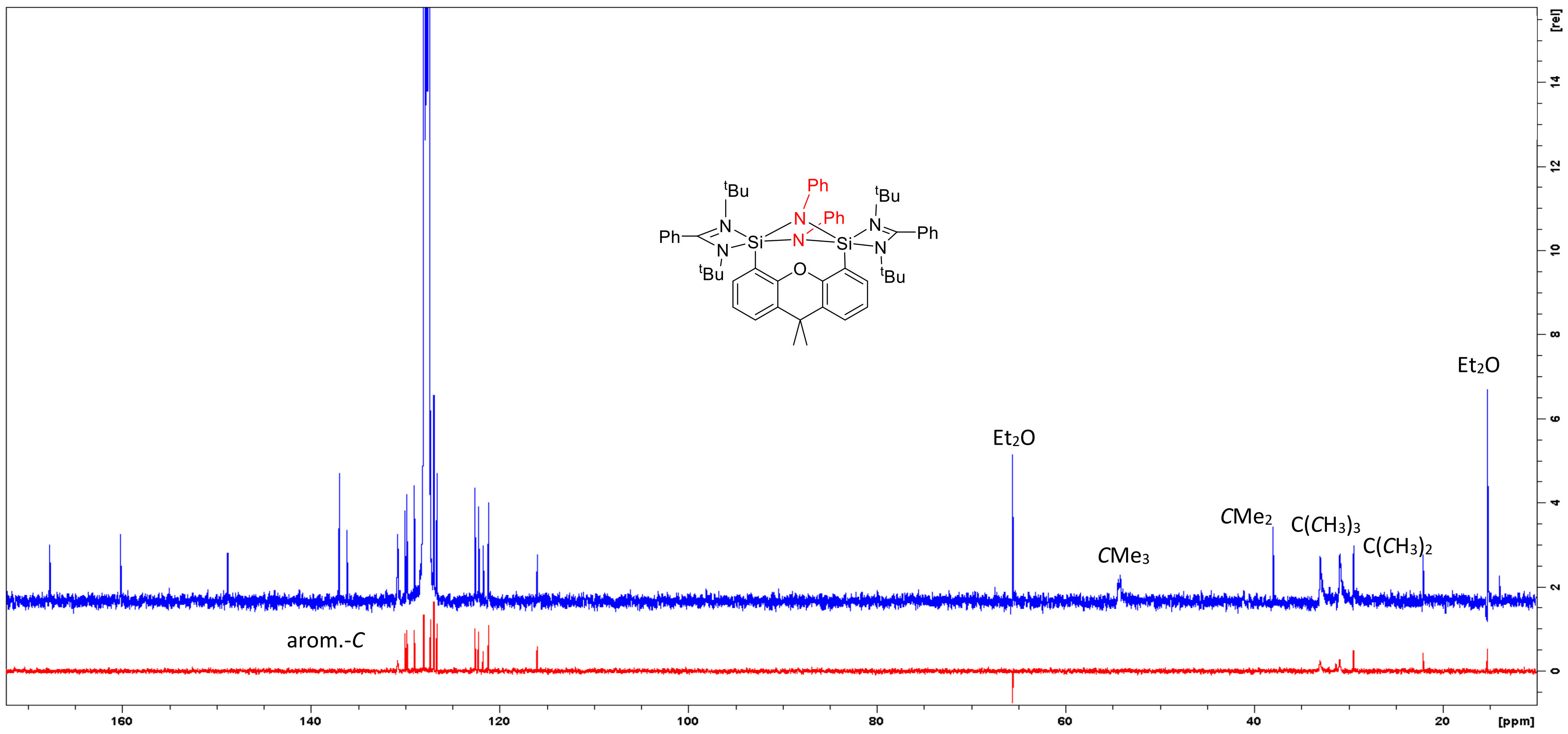

Figure S24. $^{13}C\{^{1}H\}$-NMR spectrum of compound 8 (100 MHz, C_6D_6, 298 K).

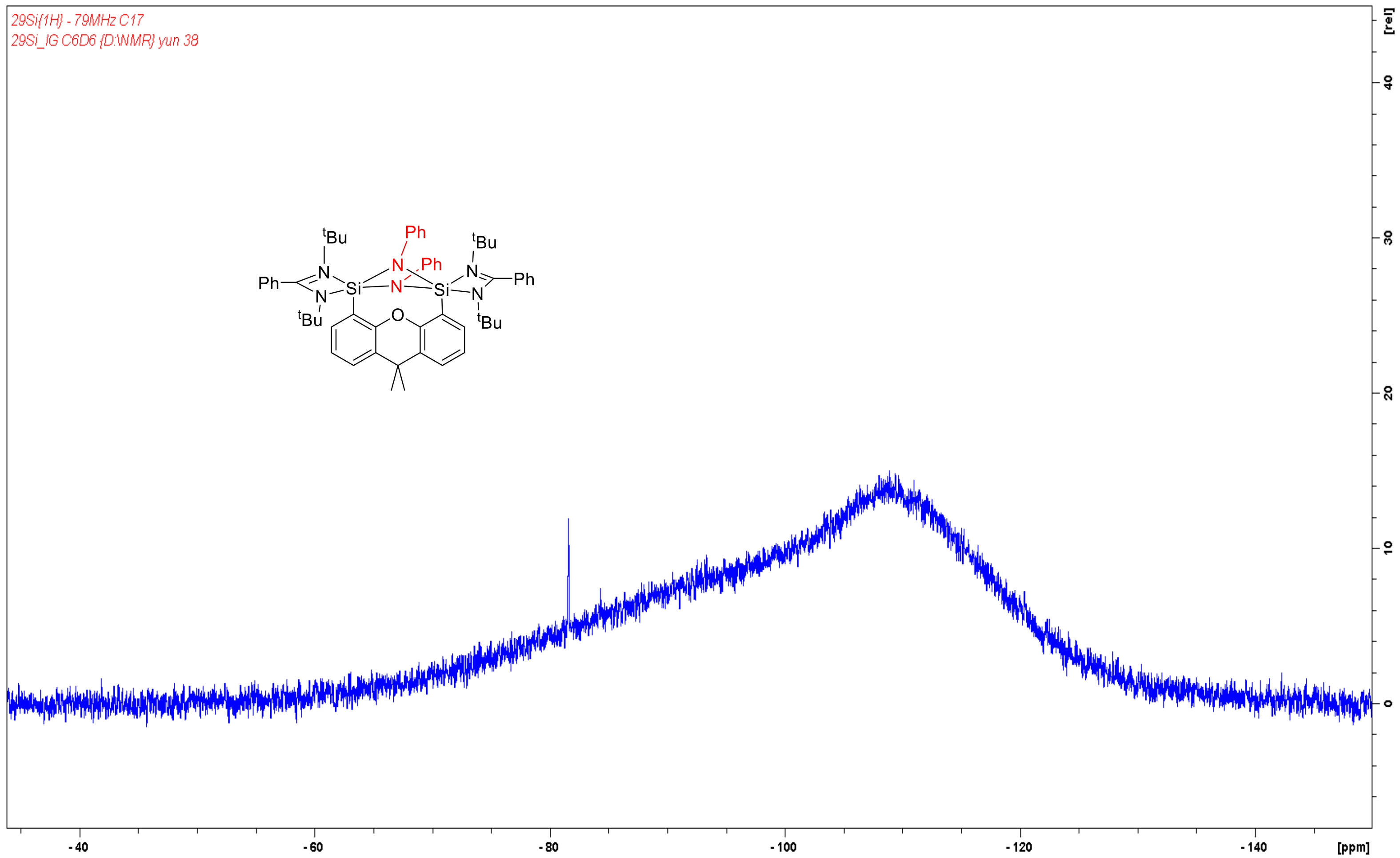

Figure S25. $^{29}Si\{^{1}H\}$-NMR spectrum of compound 8 (79 MHz, C_6D_6, 298 K).

A4. Crystallographic data

Table S1. Crystal data and structure refinement for 3.

Empirical formula	C48 H61 N7 Si2	
Formula weight	792.21	
Temperature	150.15 K	
Wavelength	1.54184 Å	
Crystal system	Monoclinic	
Space group	$P\ 2_1/n$	
Unit cell dimensions	a = 11.02920(10) Å	α= 90°.
	b = 33.4152(4) Å	β= 107.1840(10)°.
	c = 12.8445(2) Å	γ = 90°.
Volume	4522.44(10) $Å^3$	
Z	4	
Density (calculated)	1.164 Mg/m^3	
Absorption coefficient	1.018 mm^{-1}	
F(000)	1704	
Crystal size	0.45 x 0.34 x 0.23 mm^3	
Theta range for data collection	2.645 to 72.643°.	
Index ranges	-8<=h<=13, -40<=k<=38, -15<=l<=13	
Reflections collected	18301	
Independent reflections	8768 [R(int) = 0.0240]	
Completeness to theta = 67.684°	99.9 %	
Absorption correction	Semi-empirical from equivalents	
Max. and min. transmission	1.00000 and 0.37302	
Refinement method	Full-matrix least-squares on F^2	
Data / restraints / parameters	8768 / 72 / 557	
Goodness-of-fit on F^2	1.019	
Final R indices [I>2sigma(I)]	R1 = 0.0399, wR2 = 0.1039	
R indices (all data)	R1 = 0.0445, wR2 = 0.1084	
Extinction coefficient	n/a	
Largest diff. peak and hole	0.380 and -0.331 e.$Å^{-3}$	

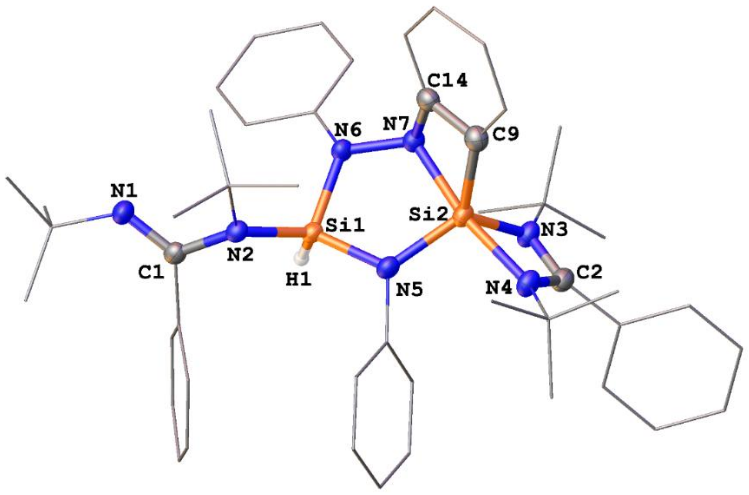

Figure S26. Molecular structure of compound **3**. Thermal ellipsoids are drawn at 50% probability level. H atoms, except the H1, are omitted for clarity.

Table S2. Selected interatomic distances [Å] and angles [°] for compound **3**.

Bond distances		Bond angels	
C1-N1	1.268(2)	N2-Si1-H1	107.4
C1-N2	1.431(2)	N5-Si1-H1	107.4
Si1-N2	1.746(1)	N5-Si1-N2	120.12(6)
Si1-N5	1.729(1)	N5-Si1-N6	99.89(5)
Si1-N6	1.741(1)	N6-Si1-H1	107.4
C2-N3	1.349(2)	N6-Si1-N2	113.91(5)
C2-N4	1.320(2)	N4-C2-N3	107.0(1)
Si2-N3	1.828(1)	C1-N2-Si1	115.48(9)
Si2-N4	1.931(1)	N3-Si2-N4	69.57(5)
Si2-N5	1.759(1)	N3-Si2-N7	102.99(5)
Si2-N7	1.873(1)	N3-Si2-C9	118.90(6)
Si2-C9	1.884(2)	N5-Si2-N3	117.30(6)
C9-C14	1.400(2)	N5-Si2-N4	98.37(5)
		N5-Si2-N7	91.57(5)
		N5-Si2-C9	123.68(6)
		N7-Si2-N4	169.55(5)
		N7-Si2-C9	73.02(6)
		C9-Si2-N4	103.69(6)

Table S3. Crystal data and structure refinement for 4.

Empirical formula	C63 H76 N7 Si2	
Formula weight	987.48	
Temperature	150.15 K	
Wavelength	1.54184 Å	
Crystal system	Monoclinic	
Space group	*P* 2/*n*	
Unit cell dimensions	a = 20.4703(2) Å	α= 90°.
	b = 12.91440(10) Å	β= 102.0700(10)°.
	c = 44.3028(5) Å	γ = 90°.
Volume	11453.0(2) Å^3	
Z	8	
Density (calculated)	1.145 Mg/m^3	
Absorption coefficient	0.899 mm^{-1}	
F(000)	4248	
Crystal size	0.39 x 0.13 x 0.12 mm^3	
Theta range for data collection	2.618 to 72.768°.	
Index ranges	-25<=h<=24, -15<=k<=14, -47<=l<=54	
Reflections collected	85152	
Independent reflections	22419 [R(int) = 0.0418]	
Completeness to theta = 67.684°	99.9 %	
Absorption correction	Semi-empirical from equivalents	
Max. and min. transmission	1.00000 and 0.83523	
Refinement method	Full-matrix least-squares on F^2	
Data / restraints / parameters	22419 / 117 / 1113	
Goodness-of-fit on F^2	1.035	
Final R indices [I>2sigma(I)]	R1 = 0.0484, wR2 = 0.1331	
R indices (all data)	R1 = 0.0587, wR2 = 0.1409	
Extinction coefficient	n/a	
Largest diff. peak and hole	0.348 and -0.318 e.Å^{-3}	

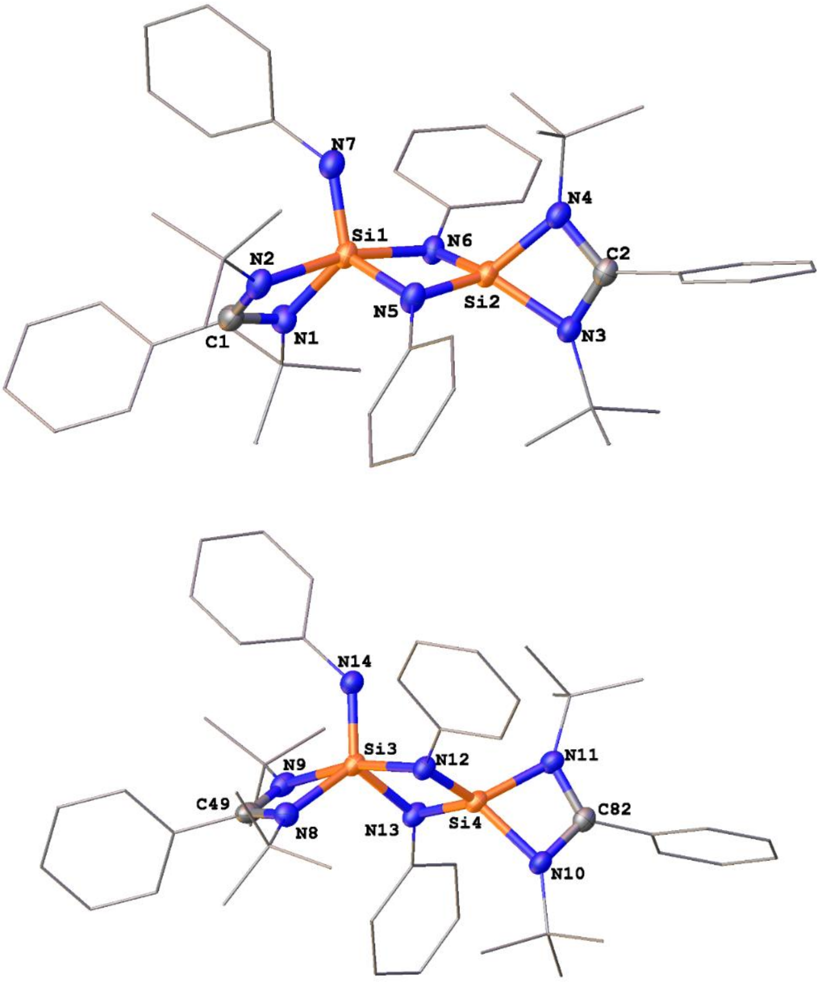

Figure S27. Molecular structure of compound 4. Mol.1 (top), Mol.2 (bottom). Thermal ellipsoids are drawn at 50% probability level. H atoms are omitted for clarity.

Table S4. Selected interatomic distances [Å] and angles [°] for compound 4.

Bond distances and angles (Mol. 1)		Bond distances and angles (Mol.2)	
C1-N1	1.366(2)	N8-C49	1.368(2)
C1-N2	1.306(2)	N9-C49	1.300(2)
N1-Si1	1.851(1)	Si3-N8	1.859(1)
Si1-N2	2.007(1)	Si3-N9	1.983(1)
Si1-N5	1.818(1)	Si3-N13	1.824(1)
Si1-N6	1.912(1)	Si3-N12	1.912(1)
Si1-N7	1.657(1)	Si3-N14	1.656(1)
C2-N3	1.349(2)	N10-C82	1.353(2)
C2-N4	1.337(2)	N11-C82	1.332(2)
Si2-N3	1.793(1)	Si4-N10	1.794(1)
Si2-N4	1.798(1)	Si4-N11	1.799(1)
Si2-N5	1.680(1)	Si4-N13	1.674(1)
Si2-N6	1.665(1)	Si4-N12	1.663(1)
N2-C1-N1	107.7(1)	N9-C49-N8	107.7(1)
C1-N1-Si1	94.1(1)	C49-N9-Si3	90.2(1)
N1-Si1-N2	67.87(6)	N8-Si3-N9	68.15(6)
N5-Si1-N1	115.48(6)	N13-Si3-N8	113.28(6)
N5-Si1-N6	79.40(6)	N13-Si3-N12	79.25(6)
N7-Si1-N1	123.06(7)	N14-Si3-N8	123.56(5)
N7-Si1-N2	96.82(6)	N14-Si3-N9	97.65(6)
N7-Si1-N5	120.17(7)	N14-Si3-N13	121.96(7)
N7-Si1-N6	96.03(6)	N14-Si3-N12	96.18(6)
N6-Si1-N2	167.15(6)	N12-Si3-N9	166.13(6)
N4-C2-N3	105.6(1)	N11-C82-N10	105.8(1)
N3-Si2-N4	73.13(6)	N10-Si4-N11	73.22(6)
N5-Si2-N3	119.13(6)	N13-Si4-N10	118.67(6)
N6-Si2-N4	122.66(6)	N12-Si4-N11	123.17(6)
N6-Si2-N5	90.92(6)	N12-Si4-N13	91.15(6)
C2-N3-Si2	90.33(9)	C82-N10-Si4	89.98(9)
Si2-N5-Si1	95.14(7)	Si4-N13-Si3	95.31(6)
Si2-N6-Si1	92.23(6)	Si4-N12-Si3	92.44(6)

Table S5. Crystal data and structure refinement for 5.

Empirical formula	C57 H68 N6 O Si2	
Formula weight	909.35	
Temperature	150.15 K	
Wavelength	1.54184 Å	
Crystal system	Triclinic	
Space group	*P*-1	
Unit cell dimensions	a = 10.7904(3) Å	α= 82.062(3)°.
	b = 13.2555(5) Å	β= 89.764(3)°.
	c = 18.1086(7) Å	γ = 89.240(3)°.
Volume	2565.06(16) $Å^3$	
Z	2	
Density (calculated)	1.177 Mg/m^3	
Absorption coefficient	0.971 mm^{-1}	
F(000)	976	
Crystal size	0.33 x 0.16 x 0.13 mm^3	
Theta range for data collection	2.464 to 72.736°.	
Index ranges	-13<=h<=11, -16<=k<=16, -22<=l<=21	
Reflections collected	18686	
Independent reflections	9875 [R(int) = 0.0508]	
Completeness to theta = 67.684°	99.8 %	
Absorption correction	Semi-empirical from equivalents	
Max. and min. transmission	1.00000 and 0.71107	
Refinement method	Full-matrix least-squares on F^2	
Data / restraints / parameters	9875 / 0 / 613	
Goodness-of-fit on F^2	1.044	
Final R indices [I>2sigma(I)]	R1 = 0.0566, wR2 = 0.1474	
R indices (all data)	R1 = 0.0653, wR2 = 0.1603	
Extinction coefficient	n/a	
Largest diff. peak and hole	0.656 and -0.546 e.$Å^{-3}$	

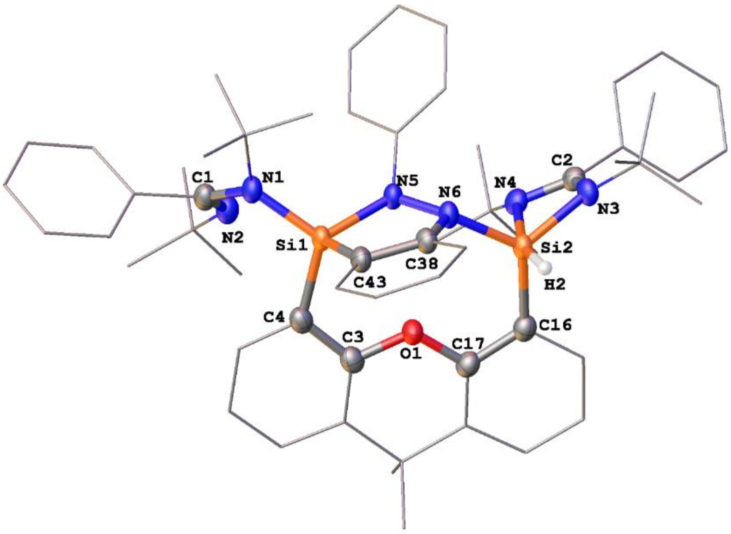

Figure S28. Molecular structure of compound **5**. Thermal ellipsoids are drawn at 50% probability level. H atoms are omitted for clarity.

Table S6. Selected interatomic distances [Å] and angles [°] for compound **5**.

Bond distances		Bond angels	
C1-N1	1.416(2)	N2-C1-N1	115.8(2)
C1-N2	1.277(3)	C1-N1-Si1	113.8(1)
N1-Si1	1.749(2)	N1-Si1-C4	112.9(1)
Si1-C4	1.891(2)	N1-Si1-N5	115.46(7)
Si1-N5	1.785(1)	N1-Si1-C43	112.87(8)
Si1-C43	1.846(2)	N5-Si1-C4	119.05(8)
C2-N3	1.376(2)	N5-Si1-C43	90.44(7)
C2-N4	1.300(2)	C43-Si1-C4	103.69(8)
Si2-N3	1.787(1)	N4-C2-N3	109.5(1)
Si2-N4	2.207(2)	N3-Si2-H2	98.3(8)
Si2-N6	1.785(2)	N3-Si2-N4	65.37(7)
Si2-C16	1.888(2)	N3-Si2-Cmo16	115.68(8)
Si2-H2	1.34(2)	N4-Si2-H2	162.8(8)
		N6-Si2-H2	94.0(9)
		N6-Si2-N3	119.52(7)
		N6-Si2-N4	98.72(6)
		N6-Si2-C16	121.53(8)
		C16-Si2-H2	95.9(9)
		C16-Si2-N4	87.23(7)

Table S7. Crystal data and structure refinement for **6**.

Empirical formula	C67 H92 N6 O Si2	
Formula weight	1053.64	
Temperature	150.15 K	
Wavelength	1.54184 Å	
Crystal system	Monoclinic	
Space group	$P\ 2_1/n$	
Unit cell dimensions	a = 15.7248(3) Å	α= 90°.
	b = 25.3350(5) Å	β= 106.226(2)°.
	c = 16.4234(3) Å	γ = 90°.
Volume	6282.3(2) $Å^3$	
Z	4	
Density (calculated)	1.114 Mg/m^3	
Absorption coefficient	0.851 mm^{-1}	
F(000)	2288	
Crystal size	0.39 x 0.21 x 0.18 mm^3	
Theta range for data collection	3.301 to 72.323°.	
Index ranges	-18<=h<=19, -31<=k<=30, -20<=l<=19	
Reflections collected	40992	
Independent reflections	12223 [R(int) = 0.0394]	
Completeness to theta = 67.684°	99.9 %	
Absorption correction	Semi-empirical from equivalents	
Max. and min. transmission	1.00000 and 0.39232	
Refinement method	Full-matrix least-squares on F^2	
Data / restraints / parameters	12223 / 0 / 609	
Goodness-of-fit on F^2	1.040	
Final R indices [I>2sigma(I)]	R1 = 0.0575, wR2 = 0.1417	
R indices (all data)	R1 = 0.0830, wR2 = 0.1630	
Extinction coefficient	n/a	
Largest diff. peak and hole	0.285 and -0.318 e.$Å^{-3}$	

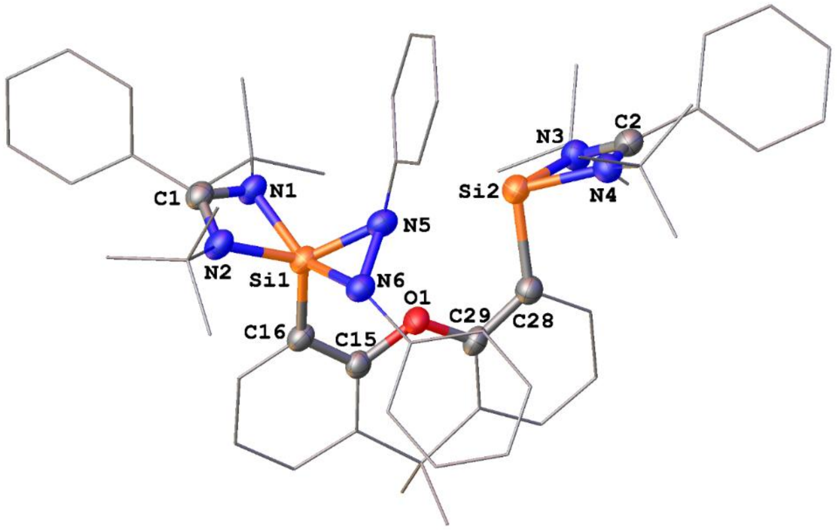

Figure S29. Molecular structure of compound 6. Thermal ellipsoids are drawn at 50% probability level. H atoms are omitted for clarity.

Table S8. Selected interatomic distances [Å] and angles [°] for compound 6.

Bond distances		Bond angels	
Si1-N1	1.908(2)	N6-Si1-N1	152.4(1)
Si1-N2	1.816(2)	N2-Si1-N1	70.35(9)
Si1-N5	1.727(2)	N2-Si1-C16	112.0(1)
Si1-N6	1.810(2)	N5-Si1-N1	108.4(1)
Si1-C16	1.870(2)	N5-Si1-N2	123.8(1)
Si2-N3	1.901(2)	N5-Si1-N6	51.03(9)
Si2-N4	1.889(2)	N5-Si1-C16	122.3(1)
Si2-C28	1.963(3)	N6-Si1-N1	152.4(1)
N5-N6	1.525(3)	N6-Si1-N2	104.0(1)
N1-C1	1.321(3)	N6-Si1-C16	104.5(1)
N2-C1	1.350(3)	C16-Si1-N1	102.6(1)
N3-C2	1.335(3)	N3-Si2-C28	95.8(1)
N4-C2	1.336(3)	N4-Si2-N3	68.53(9)
		N4-Si2-C28	99.4(1)

Table S9. Crystal data and structure refinement for **7**.

Empirical formula	C73.80 H90 N8 O2.20 Si2	
Formula weight	1180.51	
Temperature	150.15 K	
Wavelength	1.54184 Å	
Crystal system	Monoclinic	
Space group	*C* 2/*c*	
Unit cell dimensions	a = 25.9424(5) Å	α= 90°.
	b = 11.6048(3) Å	β= 91.872(2)°.
	c = 22.2947(4) Å	γ = 90°.
Volume	6708.4(2) $Å^3$	
Z	4	
Density (calculated)	1.169 Mg/m^3	
Absorption coefficient	0.876 mm^{-1}	
F(000)	2538	
Crystal size	0.13 x 0.09 x 0.04 mm^3	
Theta range for data collection	3.409 to 72.830°.	
Index ranges	-31<=h<=32, -14<=k<=13, -19<=l<=27	
Reflections collected	24643	
Independent reflections	6593 [R(int) = 0.0459]	
Completeness to theta = 67.684°	100.0 %	
Absorption correction	Semi-empirical from equivalents	
Max. and min. transmission	1.00000 and 0.28687	
Refinement method	Full-matrix least-squares on F^2	
Data / restraints / parameters	6593 / 0 / 369	
Goodness-of-fit on F^2	1.040	
Final R indices [I>2sigma(I)]	R1 = 0.0435, wR2 = 0.1147	
R indices (all data)	R1 = 0.0570, wR2 = 0.1252	
Extinction coefficient	n/a	
Largest diff. peak and hole	0.320 and -0.340 e.$Å^{-3}$	

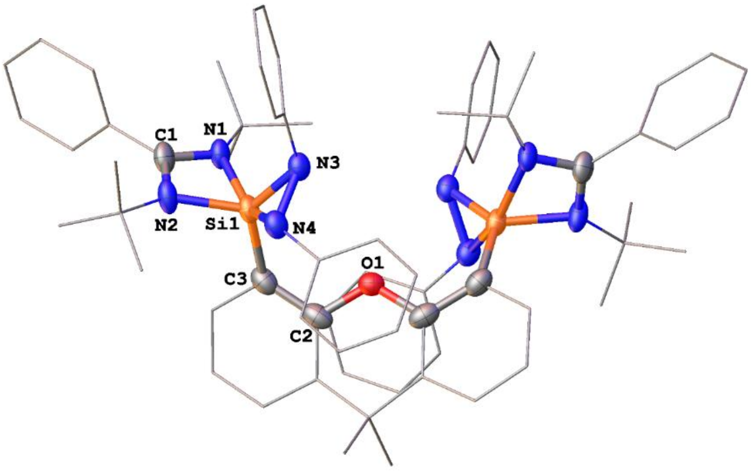

Figure S30. Molecular structure of compound 7. Thermal ellipsoids are drawn at 50% probability level. H atoms are omitted for clarity.

Table S10. Selected interatomic distances [Å] and angles [°] for compound **7**.

Bond distances		Bond angels	
Si1-N1	1.899(1)	N4-Si1-N1	150.92(6)
Si1-N2	1.815(1)	N3-Si1-N2	117.95(7)
Si1-N3	1.746(1)	N3-Si1-C3	128.90(7)
Si1-N4	1.784(1)	N2-Si1-N3	111.95(7)
Si1-C3	1.880(2)	N1-C1-N2	106.5(1)
C1-N1	1.324(2)	N2-Si1-N1	70.42(6)
C1-N2	1.349(2)	C3-Si1-N1	103.49(6)
		N3-Si1-N1	105.14(6)
		N3-Si1-N4	51.05(6)
		N4-Si1-N2	103.88(6)

Table S11. Crystal data and structure refinement for 8.

Empirical formula	C61 H78 N6 O2 Si2	
Formula weight	983.47	
Temperature	150.15 K	
Wavelength	1.54184 Å	
Crystal system	Monoclinic	
Space group	$P2_1/c$	
Unit cell dimensions	a = 12.05810(10) Å	α= 90°.
	b = 18.20650(10) Å	β= 93.8400(10)°.
	c = 25.3022(2) Å	γ = 90°.
Volume	5542.27(7) Å^3	
Z	4	
Density (calculated)	1.179 Mg/m^3	
Absorption coefficient	0.947 mm^{-1}	
F(000)	2120	
Crystal size	0.47 x 0.28 x 0.19 mm^3	
Theta range for data collection	2.993 to 72.612°.	
Index ranges	-14<=h<=14, -22<=k<=22, -31<=l<=26	
Reflections collected	41563	
Independent reflections	10871 [R(int) = 0.0271]	
Completeness to theta = 67.684°	99.9 %	
Absorption correction	Semi-empirical from equivalents	
Max. and min. transmission	1.00000 and 0.47063	
Refinement method	Full-matrix least-squares on F^2	
Data / restraints / parameters	10871 / 36 / 609	
Goodness-of-fit on F^2	1.028	
Final R indices [I>2sigma(I)]	R1 = 0.0350, wR2 = 0.0932	
R indices (all data)	R1 = 0.0394, wR2 = 0.0972	
Extinction coefficient	n/a	
Largest diff. peak and hole	0.256 and -0.365 e.Å^{-3}	

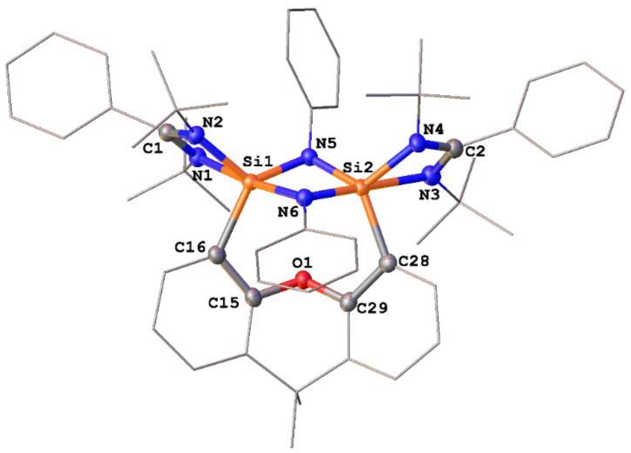

Figure S31. Molecular structure of compound 8. Thermal ellipsoids are drawn at 50% probability level. H atoms are omitted for clarity.

Table S12. Selected interatomic distances [Å] and angles [°] for compound 8.

Bond distances		Bond angels	
Si1-N1	2.028(1)	N6-Si1-N1	172.38(4)
Si1-N2	1.840(1)	N5-Si1-N2	122.35(5)
Si1-N5	1.771(1)	N5-Si1-C16	127.96(5)
Si1-N6	1.823(1)	N2-Si1-C16	105.72(5)
Si1-C16	1.919(1)	N2-Si1-N1	67.78(4)
Si2-N3	2.260(1)	N5-Si1-N1	91.99(4)
Si2-N4	1.799(1)	N5-Si1-N6	80.92(4)
Si2-N5	1.750(1)	N6-Si1-N2	113.70(5)
Si2-N6	1.795(1)	N6-Si1-C16	98.20(5)
Si2-C28	1.915(1)	C16-Si1-N1	88.37(5)
N1-C1	1.298(2)	N6-Si2-N3	177.73(4)
N2-C1	1.372(1)	N4-Si2-C28	108.30(5)
		N5-Si2-N4	117.05(5)
		N5-Si2-C28	129.23(5)
		N4-Si2-N3	64.79(4)
		N5-Si2-N3	95.88(4)
		N5-Si2-N6	82.29(4)
		N6-Si2-N4	117.23(5)
		N6-Si2-C28	96.88(5)
		C28-Si2-N3	83.23(4)

B. Computational Section

Computational Details

DFT calculations were performed using Gaussian 16 (Revision C.01)[7] with the B3LYP density functional.[8–10] The def2-SVP basis set was employed for all atoms. Geometry optimizations included Grimme's D3 dispersion correction with the original D3 damping function.[11] Frequency calculations at the same level of theory confirmed the nature of the stationary points (zero imaginary frequencies for local minima and one for transition states) and provided thermal corrections for Gibbs free energy. Transition states were further validated through intrinsic reaction coordinate (IRC) calculations to connect them to the corresponding minima. Single-point energy calculations were carried out at the B3LYP-D3(BJ)/def2-TZVP level in solution (THF), using the gas-phase optimized geometries. The SMD solvation model was applied with Bondi radii defining the molecular cavity.[12] Gibbs free energies in solution were obtained by adding the thermal corrections from frequency calculations to the single-point energies. Intrinsic bond orbital (IBO)[13,14] analysis was performed using ORCA[15] at the B3LYP/def2-TZVP level and visualized with IBOview. [16,17]

Table S13. Key structural parameters (Å) of experimental and DFT-optimized structures as well as the Wiberg bond indices (WBIs) of **4** at the B3LYP-D3(BJ)/def2-SVP level of theory.

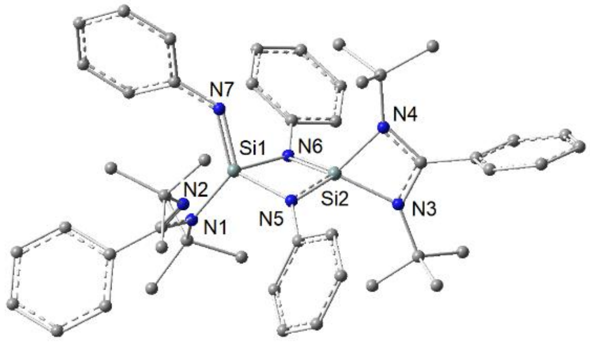

	Experimental (Å)	Calculated (Å)	WBIs
Si1-N7	1.657(1)	1.65890	**1.10**
Si1-N1	1.851(1)	1.88043	0.59
Si1-N2	2.007(1)	2.00584	0.43
Si1-N5	1.818(1)	1.84396	0.64
Si1-N6	1.912(1)	1.97623	0.50
Si2-N5	1.680(1)	1.69094	0.92
Si2-N6	1.665(1)	1.66811	**1.03**
Si2-N3	1.793(1)	1.82345	0.64
Si2-N4	1.798(1)	1.83255	0.59

Table S14. Key structural parameters (Å) of experimental and DFT-optimized structures as well as the Wiberg bond indices (WBIs) of **8** at the B3LYP-D3(BJ)/def2-SVP level of theory.

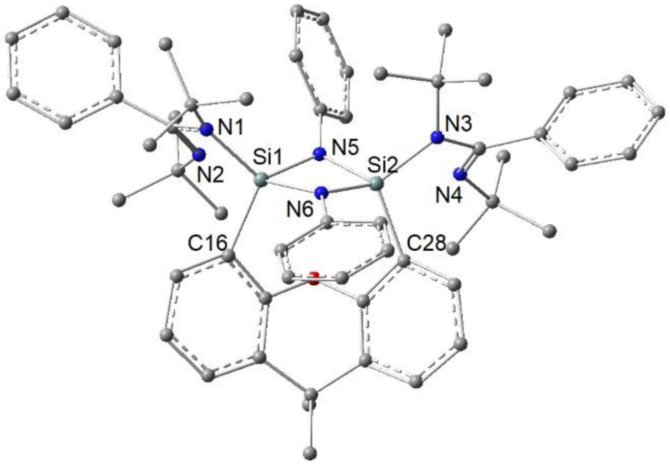

	Experimental (Å)	Calculated (Å)	WBIs
Si1-N1	1.840(1)	1.87726	0.67
Si1-N2	2.028(1)	2.08631	0.38
Si1-N5	1.771(1)	1.79894	0.78
Si1-N6	1.823(1)	1.85455	0.69
Si1-C16	1.919(1)	1.93324	0.76
Si2-N5	1.750(1)	1.77217	0.80
Si2-N6	1.795(1)	1.78819	0.78
Si2-N3	1.799(1)	1.81076	0.77
Si2-N4	2.260(1)	2.64231	0.11
Si2-C28	1.915(1)	1.92145	0.79

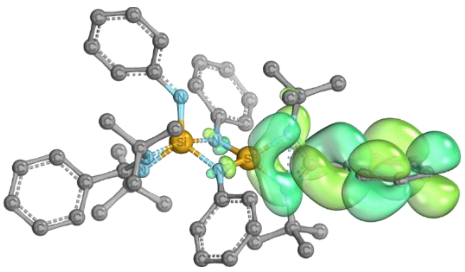

LUMO (-1.7285 eV)

Figure S32. The LUMO of compound **4**.

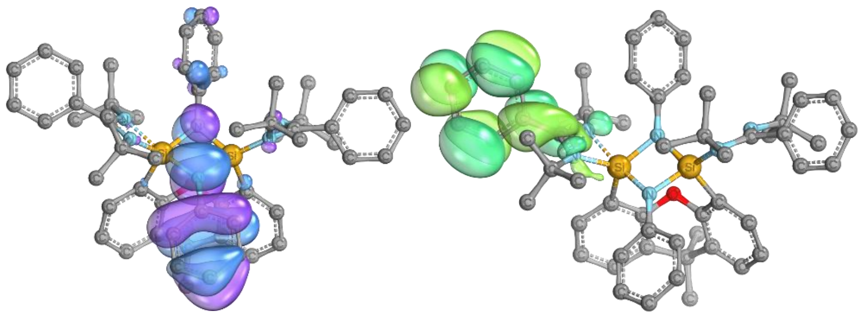

HOMO (-4.9511eV)   LUMO (-0.9966 eV)

***Figure S33*.** The HOMO (left) and LUMO (right) of compound **8**.

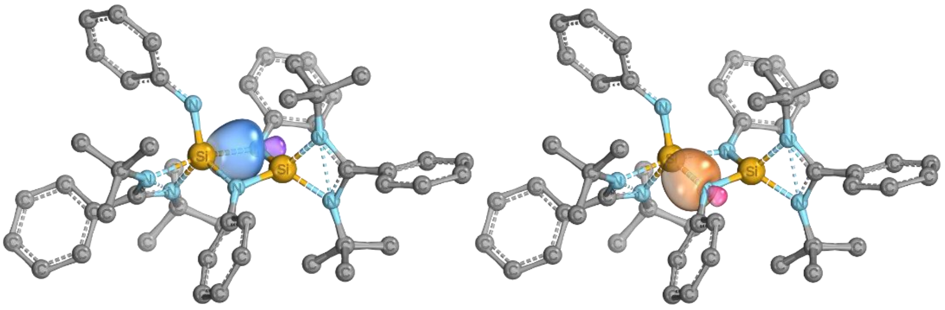

a) Si1-N6 σ-bonding orbital   b) Si1-N5 σ-bonding orbital

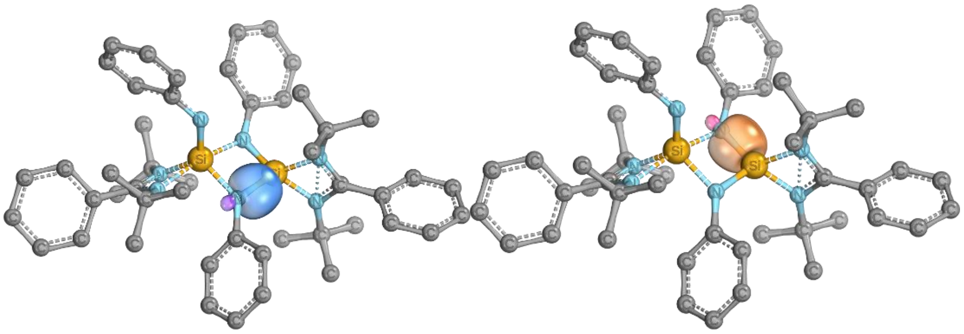

c) Si2-N5 σ-bonding orbital   d) Si2-N6 σ-bonding orbital

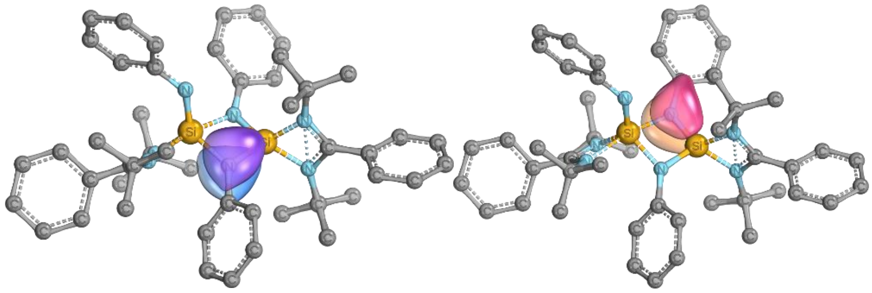

e) N5 long pair orbital   f) N6 lone pair orbital

***Figure S34*.** Depiction of selected IBOs a) - f) of compound **4**.

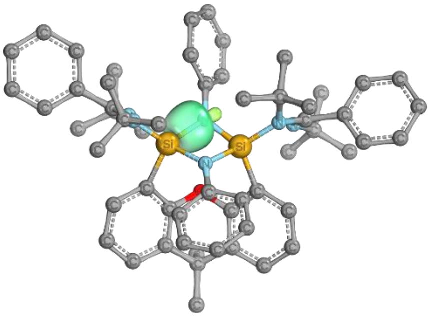

a) Si1-N5 σ-bonding orbital

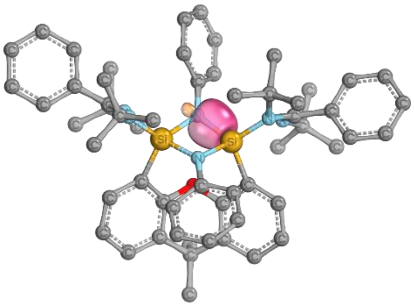

b) Si2-N5 σ-bonding orbital

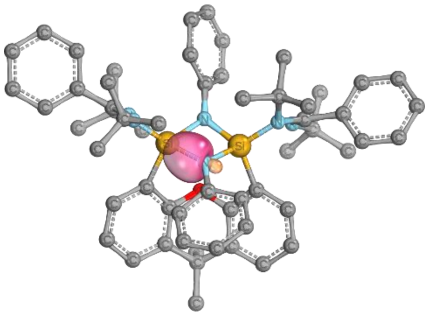

C) Si1-N5 σ-bonding orbital

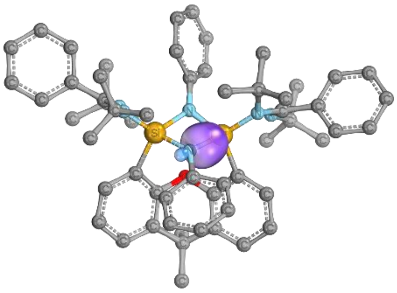

d) Si2-N5 σ-bonding orbital

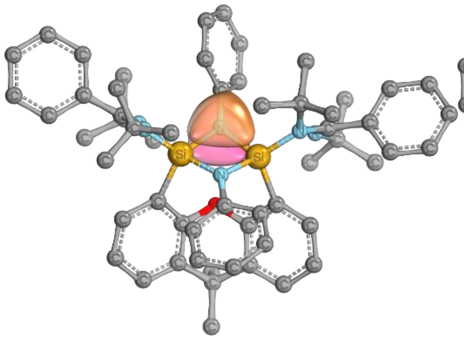

e) N5 lone pair orbital

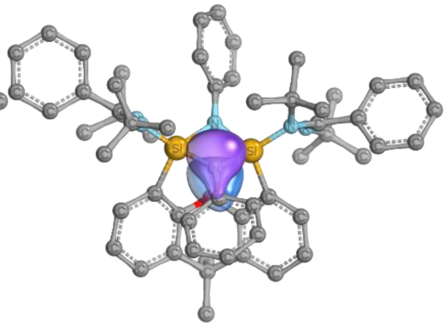

f) N6 lone pair orbital

Figure S35. Depiction of selected IBOs a) - f) of compound **8**.

Table S15. Gibbs free energy changes of the reaction of **1** and **2** with *trans*-azobenzene to **3** and **5**, respectively.

**Compounds**	**Gibbs free energy changes with respect to reactants** (Kcal mol^{-1})
**3**	-43.4
**3´** (hypothetic isomer of **3** with a five-membered SiN_2C_2 ring)	-28.1
**5**	-46.8
**5´** (hypothetic isomer of **5** with a four-membered $SiNC_2$ ring)	-33.2

Optimized x,y,z-coordinates

***cis*-N_2Ph_2**

C	2.52471000	0.69194200	-0.77611300
C	1.41949900	0.78340700	0.08415200
C	1.22612900	-0.18764800	1.08216300
C	2.11799200	-1.25494400	1.18939500
C	3.19204300	-1.37402900	0.29953400
C	3.39075200	-0.39842100	-0.68422300
C	-2.11796900	-1.25498800	-1.18935500
C	-3.19204200	-1.37404300	-0.29951500
C	-3.39077300	-0.39840100	0.68420500
C	-2.52473100	0.69196300	0.77607700
C	-1.41949800	0.78339700	-0.08416500
C	-1.22610600	-0.18769100	-1.08213800
N	0.62154300	1.96475200	-0.00706500
N	-0.62154800	1.96474800	0.00704600
H	2.68222300	1.48183200	-1.51390400
H	0.38485600	-0.09720500	1.77094200
H	1.97034100	-2.00344400	1.97208600
H	3.88131300	-2.21777800	0.38262900
H	4.23617600	-0.47826000	-1.37227200
H	-1.97030100	-2.00351500	-1.97201800
H	-3.88131000	-2.21779400	-0.38259900
H	-4.23621400	-0.47821600	1.37223500
H	-2.68226100	1.48188100	1.51383500
H	-0.38481800	-0.09727500	-1.77090100

***trans*-N_2Ph_2**

C	-2.63634100	-1.28980100	0.00000000
C	-1.76788300	-0.18642400	0.00000000
C	-2.29489100	1.12042200	0.00000000
C	-3.67444400	1.30629500	0.00000000
C	-4.54015200	0.20180200	-0.00000100
C	-4.01904000	-1.09582900	-0.00000100
C	3.67444400	-1.30629500	-0.00000100
C	4.54015200	-0.20180200	-0.00000100
C	4.01904000	1.09582900	0.00000000
C	2.63634100	1.28980100	0.00000000
C	1.76788300	0.18642400	0.00000100
C	2.29489100	-1.12042200	0.00000000
N	-0.38705200	-0.49179000	0.00000100
N	0.38705200	0.49179000	0.00000100
H	-2.19758400	-2.28984600	0.00000000
H	-1.60197700	1.96254900	0.00000100
H	-4.08591400	2.31921200	0.00000000
H	-5.62211800	0.35708500	-0.00000100

H	-4.69136300	-1.95734300	-0.00000100
H	4.08591400	-2.31921200	-0.00000100
H	5.62211800	-0.35708500	-0.00000200
H	4.69136300	1.95734300	-0.00000100
H	2.19758400	2.28984600	0.00000000
H	1.60197700	-1.96254900	0.00000100

1

Si	1.45265000	-0.36032400	-1.78908900
Si	-1.45259000	0.36057000	-1.78908900
N	0.00001200	0.00005800	-0.77534300
N	2.56838200	-1.23383800	-0.47290100
N	2.82677700	0.82278800	-1.05910800
N	-2.82675800	-0.82264000	-1.05933600
N	-2.56836800	1.23389300	-0.47280700
C	0.00000200	-0.00003500	0.63730300
C	3.38741000	-0.18438300	-0.38254300
C	-3.38741900	0.18443900	-0.38265700
C	-4.69934600	0.13600900	0.32903400
C	4.69929700	-0.13605100	0.32922900
C	2.65501300	-2.58322200	0.11048800
C	-3.36752900	-2.11567100	-1.50666800
C	-2.65497800	2.58318400	0.11080100
C	-0.79571800	-0.90500400	1.37404300
H	-1.41734400	-1.61107300	0.82691200
C	3.36755700	2.11589100	-1.50622300
C	-5.89617600	0.46361500	-0.32291300
H	-5.87097800	0.79432800	-1.36340700
C	-4.73183800	-0.27518100	1.67017100
H	-3.79546500	-0.52834000	2.17243000
C	3.89891300	-3.33432800	-0.40300200
H	3.91207500	-3.34588000	-1.50421300
H	3.88787100	-4.37659500	-0.04590400
H	4.82828000	-2.86881400	-0.04733000
C	0.79572800	0.90482800	1.37416500
H	1.41736700	1.61096100	0.82713100
C	4.73171900	0.27499200	1.67041200
H	3.79532300	0.52812100	2.17264200
C	-0.79921800	-0.90014800	2.77046800
H	-1.42664100	-1.61814800	3.30721100
C	1.39469000	-3.33820800	-0.34478000
H	0.49164300	-2.83848400	0.02982300
H	1.40742900	-4.36778900	0.04370400
H	1.33650000	-3.38424200	-1.44344900
C	5.89615700	-0.46361700	-0.32268400
H	5.87101100	-0.79422200	-1.36321400
C	-0.00001100	-0.00021600	3.48404800

H	-0.00001500	-0.00028600	4.57659700
C	0.79921200	0.89979800	2.77059000
H	1.42663300	1.61772900	3.30743000
C	2.66531600	-2.51580700	1.65006700
H	3.59130000	-2.06039500	2.02638500
H	2.59789000	-3.53197100	2.07055500
H	1.81129200	-1.92867900	2.01707400
C	-5.94942500	-0.36024300	2.34871900
H	-5.96709700	-0.68120100	3.39318700
C	2.16610200	2.92349100	-2.03037600
H	1.65114200	2.38485700	-2.84121200
H	2.49939100	3.89637400	-2.42280300
H	1.43536500	3.10379400	-1.22909100
C	4.03015900	2.90361700	-0.36009900
H	3.35270500	2.98597700	0.50387400
H	4.26886400	3.92206600	-0.70465900
H	4.96462900	2.43836700	-0.02080600
C	5.94926900	0.35995700	2.34903800
H	5.96688900	0.68080700	3.39354000
C	7.11385800	-0.37558800	0.35791200
H	8.04301400	-0.63212900	-0.15700900
C	-1.39465700	3.33822600	-0.34437000
H	-0.49160900	2.83842500	0.03012700
H	-1.40737000	4.36774100	0.04429000
H	-1.33649100	3.38444600	-1.44303300
C	-2.66524400	2.51552100	1.65037200
H	-3.59122000	2.06005200	2.02663800
H	-2.59780700	3.53161800	2.07102000
H	-1.81121300	1.92833100	2.01726500
C	-3.89887600	3.33439400	-0.40254200
H	-3.91208300	3.34609200	-1.50375100
H	-3.88778700	4.37661400	-0.04530800
H	-4.82824300	2.86886300	-0.04689400
C	4.37841400	1.90684100	-2.65169600
H	5.26165500	1.35401200	-2.29938100
H	4.72176000	2.87538400	-3.05011400
H	3.91327600	1.33688700	-3.47046800
C	-7.11391400	0.37548500	0.35760300
H	-8.04304600	0.63205700	-0.15734600
C	-7.14271400	-0.03780500	1.69330400
H	-8.09539600	-0.10687800	2.22436500
C	7.14258900	0.03756200	1.69365800
H	8.09524200	0.10655700	2.22478100
C	-2.16606100	-2.92319300	-2.03090800
H	-1.65108100	-2.38444100	-2.84165400
H	-2.49933800	-3.89602100	-2.42348400
H	-1.43534400	-3.10361000	-1.22963000
C	-4.37835300	-1.90644000	-2.65213700
H	-5.26159600	-1.35364900	-2.29976300

H	-4.72170200	-2.87492000	-3.05070500
H	-3.91318500	-1.33637300	-3.47081300
C	-4.03016500	-2.90357200	-0.36068300
H	-3.35272800	-2.98608000	0.50328900
H	-4.26887800	-3.92196200	-0.70540900
H	-4.96463500	-2.43836200	-0.02133500

TS1(aniline-based)

Si	-1.78486700	0.60019000	0.93738900
Si	1.01769900	-0.05289600	0.38841600
N	-0.65963900	-0.59865100	0.18131600
N	-3.36575300	-0.47539600	1.11810200
N	-3.08998000	0.96395700	-0.45913600
N	2.02403800	-1.50885700	1.18966300
N	2.01004900	-1.00594900	-0.91124400
C	-1.04685900	-1.80865900	-0.44089000
C	-3.99551600	0.19001200	0.14509300
C	2.65025900	-1.71839000	0.02535600
C	3.91192800	-2.49141700	-0.15988400
C	-5.45150100	0.12981300	-0.17472400
C	-3.83650600	-1.50749100	2.05630300
C	2.44801500	-1.73487300	2.58133400
C	2.10730100	-1.07563200	-2.38997800
C	-0.57878700	-3.05294100	0.03245800
H	0.10240700	-3.06308000	0.88273200
C	-3.21518600	2.06896700	-1.42468900
C	5.14164300	-1.81631400	-0.16012600
H	5.15899000	-0.72764200	-0.06746900
C	3.88527000	-3.88559400	-0.30242300
H	2.92530000	-4.40636900	-0.29824500
C	-4.98349100	-0.98472000	2.94247100
H	-4.68149700	-0.05650900	3.45265400
H	-5.24153800	-1.73273300	3.70915300
H	-5.88940700	-0.78049300	2.35545400
C	-1.92290000	-1.81915500	-1.54719000
H	-2.29259400	-0.86821900	-1.92671700
C	-5.91976600	-0.80296200	-1.11069300
H	-5.21327300	-1.49376300	-1.57646200
C	-0.96799200	-4.24946300	-0.57364200
H	-0.58830600	-5.19768600	-0.18202900
C	-2.63577200	-1.86393600	2.94950800
H	-1.80746900	-2.25455200	2.34269600
H	-2.92232800	-2.63388300	3.68177600
H	-2.27650800	-0.98051900	3.50021700
C	-6.35059500	1.02494900	0.42209900
H	-5.98466400	1.74834900	1.15419600
C	-1.84074000	-4.24433600	-1.66681500

H	-2.14612400	-5.18118500	-2.13891400
C	-2.31408700	-3.01804200	-2.14627200
H	-2.99180000	-2.99197700	-3.00476900
C	-4.27944200	-2.77051700	1.29255500
H	-5.17743500	-2.57795900	0.68963800
H	-4.52064500	-3.57605200	2.00450100
H	-3.47816600	-3.12058200	0.62593800
C	5.07806600	-4.59855400	-0.44352500
H	5.05091800	-5.68497600	-0.55789500
C	-1.85321500	2.17642600	-2.13454500
H	-1.02217900	2.26083300	-1.41990400
H	-1.83276200	3.06130700	-2.78978300
H	-1.66364400	1.28802100	-2.75491100
C	-4.30197000	1.81897800	-2.48578000
H	-4.17926800	0.82795700	-2.94895700
H	-4.21179200	2.57904700	-3.27739200
H	-5.31793900	1.88260500	-2.07529300
C	-7.27513300	-0.83825700	-1.44714900
H	-7.63400000	-1.56643000	-2.17872500
C	-7.70553700	0.98851100	0.08334100
H	-8.40152500	1.68912100	0.55139500
C	1.01752800	-0.15905900	-2.96889700
H	0.02327200	-0.47380200	-2.62892600
H	1.03653500	-0.22021100	-4.06795400
H	1.18977800	0.88419500	-2.67816500
C	1.84014300	-2.51875600	-2.85917900
H	2.62707000	-3.20766700	-2.52204000
H	1.81810000	-2.55321100	-3.95980300
H	0.87138000	-2.87675600	-2.48043000
C	3.48104600	-0.58566900	-2.88405200
H	3.67879300	0.42390600	-2.49703500
H	3.48306400	-0.53933200	-3.98468900
H	4.29239200	-1.25970800	-2.57773900
C	-3.50985900	3.38096500	-0.66943400
H	-4.46554400	3.30925700	-0.12672200
H	-3.57844100	4.22663800	-1.37302200
H	-2.71102600	3.59625600	0.05647900
C	6.33370600	-2.53285700	-0.29288700
H	7.28855400	-2.00143900	-0.29098600
C	6.30370500	-3.92414300	-0.43511900
H	7.23654800	-4.48331900	-0.54238800
C	-8.16952200	0.05799300	-0.85270300
H	-9.22926400	0.03120700	-1.11844600
C	1.19096700	-1.52255600	3.44495400
H	0.75523900	-0.52592000	3.27098000
H	1.44056500	-1.60681100	4.51377800
H	0.42196600	-2.27237900	3.20848100
C	3.52494300	-0.71138400	2.99118400
H	4.41161300	-0.79173700	2.34504800

H	3.84410300	-0.87889700	4.03250800
H	3.12713900	0.31201500	2.91625600
C	2.97048700	-3.16468700	2.80966500
H	2.26455900	-3.90681100	2.40592000
H	3.07888900	-3.34652200	3.89039000
H	3.94989900	-3.32960200	2.34283700
C	0.43344500	4.41201900	0.00537600
C	1.09847100	3.25602000	0.45320000
C	1.32758200	3.08693100	1.82425900
C	0.87501900	4.04794600	2.73360700
C	0.20055400	5.18693300	2.28941300
C	-0.00863400	5.36997600	0.91776300
C	5.45411600	3.06242300	0.68320600
C	6.09993600	3.99494800	-0.13578100
C	5.49189800	4.40213600	-1.33019900
C	4.25171300	3.88510400	-1.70491100
C	3.58338300	2.97863700	-0.86439800
C	4.20116300	2.55858300	0.32943300
N	1.36126800	2.28615300	-0.58055100
N	2.39034000	2.37272000	-1.31424900
H	0.24821500	4.53299300	-1.06380400
H	1.88931900	2.22055400	2.16450700
H	1.06057500	3.90210300	3.80092500
H	-0.15353600	5.93280800	3.00531500
H	-0.52965100	6.25948700	0.55390400
H	5.92819300	2.72501700	1.60917900
H	7.07561600	4.39590600	0.14942400
H	5.99454200	5.12354300	-1.98005000
H	3.77802800	4.17599300	-2.64516600
H	3.70320000	1.81221700	0.94981500

Int1(aniline-based)

Si	-1.78897800	0.71030600	0.87304000
Si	1.16713600	0.37556500	0.09542300
N	-0.42997300	-0.31173000	0.16124500
N	-3.16349100	-0.61580900	1.08474200
N	-3.04003900	0.72332100	-0.59329900
N	2.42518700	-0.45896800	1.14738500
N	2.15782900	-0.95791600	-0.93665600
C	-0.67109900	-1.66605300	-0.19823400
C	-3.84589700	-0.13229400	0.03755200
C	2.91374000	-1.18854800	0.12463300
C	4.13272900	-2.03675900	0.18852800
C	-5.25664400	-0.44242200	-0.33391300
C	-3.58478800	-1.49143600	2.19219000
C	2.76692400	-0.38093300	2.58582000
C	2.29071900	-1.42479000	-2.33585800

C	-0.10428000	-2.72186000	0.54485500
H	0.55331000	-2.47773700	1.38006200
C	-3.22759200	1.63810500	-1.73066100
C	5.38880700	-1.41402900	0.14279900
H	5.44869900	-0.32835800	0.03553600
C	4.04282700	-3.42823100	0.31788300
H	3.06102800	-3.90339300	0.36750900
C	-4.80147600	-0.91279700	2.94011000
H	-4.59260100	0.11634100	3.27207400
H	-5.02744500	-1.52399600	3.82849500
H	-5.69877000	-0.89961500	2.30620200
C	-1.51651100	-2.00605300	-1.27261400
H	-1.96177200	-1.20296900	-1.85715100
C	-5.53277200	-1.58400500	-1.09994600
H	-4.71258600	-2.24573400	-1.38703300
C	-0.37333000	-4.05589000	0.23228300
H	0.08020200	-4.85125800	0.83046900
C	-2.39401700	-1.56910700	3.16411600
H	-1.51250300	-1.99079900	2.66146200
H	-2.64409700	-2.21419200	4.01991500
H	-2.13204600	-0.57112400	3.54885900
C	-6.30264600	0.41665500	0.03210400
H	-6.08762600	1.30199100	0.63428300
C	-1.22265000	-4.37717400	-0.83204500
H	-1.43695500	-5.42023000	-1.07594500
C	-1.79043100	-3.34065800	-1.58073000
H	-2.44908000	-3.57244000	-2.42267800
C	-3.89556400	-2.90737400	1.67226600
H	-4.78743900	-2.91138500	1.03094400
H	-4.08875600	-3.58593100	2.51850100
H	-3.04709900	-3.30051900	1.09433300
C	5.20744100	-4.19516700	0.40296600
H	5.13569500	-5.28019700	0.51018600
C	-1.82094400	1.92648800	-2.28808900
H	-1.16003600	2.35387800	-1.52002500
H	-1.88118100	2.64941600	-3.11599700
H	-1.34670100	1.00794000	-2.66322600
C	-4.08998000	1.01889700	-2.84457300
H	-3.70502000	0.02980200	-3.13827300
H	-4.06160100	1.67199200	-3.73042400
H	-5.14029500	0.90337200	-2.54699600
C	-6.84267900	-1.86103600	-1.49855000
H	-7.05093800	-2.74996500	-2.09909300
C	-7.61239300	0.13555600	-0.36467500
H	-8.42369100	0.80753300	-0.07404900
C	1.23343200	-0.65881500	-3.15068200
H	0.22362200	-0.93041800	-2.81764100
H	1.32218200	-0.91376100	-4.21726600
H	1.34310300	0.42631800	-3.03669200

C	1.99107600	-2.93186500	-2.44782200
H	2.75597900	-3.53227200	-1.93767000
H	1.99091400	-3.22772000	-3.50894600
H	1.00957400	-3.17418400	-2.02235300
C	3.69683800	-1.13837400	-2.89738700
H	3.98366000	-0.09096700	-2.73476800
H	3.70999900	-1.33885400	-3.98021100
H	4.45589400	-1.78212800	-2.43260300
C	-3.85840800	2.95633500	-1.23908900
H	-4.86439800	2.77934400	-0.82904400
H	-3.95010600	3.67392300	-2.07028800
H	-3.23363900	3.41218200	-0.45583300
C	6.54981700	-2.18600900	0.22054700
H	7.52749200	-1.70004100	0.17813300
C	6.46078600	-3.57626800	0.35312100
H	7.37041700	-4.17845100	0.41777400
C	-7.88402900	-1.00184400	-1.13269100
H	-8.90841000	-1.21873400	-1.44564600
C	1.44190600	-0.18729100	3.34838800
H	0.89593400	0.68921500	2.97797400
H	1.64313100	-0.03828100	4.42012400
H	0.78921900	-1.06615500	3.23969900
C	3.68318600	0.83164500	2.82260500
H	4.61494300	0.73299500	2.24497800
H	3.94223000	0.91671500	3.88998100
H	3.17404900	1.75007300	2.50311400
C	3.43789800	-1.66080100	3.11268200
H	2.85180500	-2.55572500	2.85356800
H	3.49734700	-1.59971100	4.21001400
H	4.45789900	-1.79431100	2.73170300
C	0.23057100	4.23712300	0.03090200
C	0.45385000	3.23133100	0.99429400
C	-0.01425300	3.45275600	2.30941400
C	-0.70719600	4.61569100	2.63177800
C	-0.94746500	5.60218500	1.66477100
C	-0.46713000	5.40051800	0.36823400
C	4.90951600	3.63509600	-0.46921100
C	5.38341600	3.50181400	-1.77856600
C	4.57557400	2.86028700	-2.72432800
C	3.32945200	2.34565000	-2.36433500
C	2.86847000	2.42898500	-1.03444400
C	3.66865200	3.11370800	-0.09729800
N	1.16163300	2.06981000	0.70399900
N	1.61255000	1.88534100	-0.70741800
H	0.60472500	4.08989800	-0.98201600
H	0.16348600	2.69252500	3.07111500
H	-1.06641500	4.75562000	3.65553700
H	-1.48910500	6.51516700	1.92371400
H	-0.63175600	6.16229900	-0.39974900

H	5.51159100	4.15969500	0.27843500
H	6.35487100	3.91260500	-2.06400000
H	4.91051600	2.77202800	-3.76182200
H	2.69098100	1.88647500	-3.11859900
H	3.29676300	3.23602900	0.91828200

Ts2 (aniline-based)

Si	-1.59547700	0.59207300	1.12111100
Si	1.03156900	0.19192900	-0.00675300
N	-0.48071400	-0.60211700	0.25136800
N	-3.16522400	-0.60500100	1.13721800
N	-2.89673300	1.03433700	-0.25647400
N	2.45904200	-0.43427600	1.01610300
N	2.05434900	-1.11837200	-1.00420400
C	-0.79387700	-1.91842300	-0.12422600
C	-3.76214300	0.10964000	0.18657800
C	2.87892000	-1.25226200	0.01828500
C	4.09394300	-2.10963800	0.06249800
C	-5.17289800	-0.07688800	-0.27313700
C	-3.71749400	-1.52808600	2.14422900
C	2.78878500	-0.49789400	2.47064000
C	2.14613300	-1.58853400	-2.40993000
C	-0.21154500	-3.02130400	0.53407900
H	0.48395200	-2.82602800	1.35187200
C	-2.92418700	1.95375500	-1.41268300
C	5.36319400	-1.51524400	0.02390500
H	5.44755200	-0.42865400	-0.04364300
C	3.98059700	-3.50528400	0.13489100
H	2.99055600	-3.96153100	0.18670000
C	-4.84170600	-0.86764800	2.96733500
H	-4.49507300	0.08973300	3.38718000
H	-5.13858000	-1.52373800	3.80150400
H	-5.73557600	-0.68008200	2.35730900
C	-1.69926900	-2.18944300	-1.17097500
H	-2.14767600	-1.34543900	-1.69238100
C	-5.45952900	-1.07151400	-1.21884500
H	-4.64983200	-1.70240100	-1.59146400
C	-0.52057500	-4.33335800	0.16750100
H	-0.05626200	-5.16723200	0.70182300
C	-2.55779200	-1.87914700	3.09573300
H	-1.74040800	-2.36653500	2.54642500
H	-2.90591300	-2.56959200	3.87941800
H	-2.16043500	-0.97346200	3.57954700
C	-6.21144200	0.72577800	0.21848700
H	-5.98800900	1.50069000	0.95514300
C	-1.42219700	-4.58370800	-0.87257200
H	-1.66540900	-5.60900600	-1.16110200

C	-2.00713000	-3.50004000	-1.53892000
H	-2.71003800	-3.67867300	-2.35776700
C	-4.22647800	-2.82669200	1.49035300
H	-5.09613700	-2.64045000	0.84551100
H	-4.53337900	-3.54333300	2.26923400
H	-3.43491400	-3.28903400	0.88378700
C	5.12939100	-4.29804500	0.17150300
H	5.03596100	-5.38465500	0.23755200
C	-2.09822000	1.33448800	-2.55813100
H	-1.07729500	1.11469400	-2.21986100
H	-2.03537300	2.03186100	-3.40902900
H	-2.57042400	0.40657100	-2.91854300
C	-4.33394000	2.28513700	-1.93251500
H	-4.85029100	1.41053200	-2.34876100
H	-4.24110400	3.03139200	-2.73685100
H	-4.96606400	2.71572500	-1.14312500
C	-6.76762000	-1.25317800	-1.67582800
H	-6.98149100	-2.02780400	-2.41651100
C	-7.51964000	0.53919400	-0.23306900
H	-8.32374100	1.16878000	0.15622800
C	1.09671200	-0.80070700	-3.21333000
H	0.09118800	-0.95922500	-2.80099600
H	1.09655800	-1.14775000	-4.25757400
H	1.30682900	0.27683500	-3.20321900
C	1.81004400	-3.08876700	-2.50380000
H	2.57728800	-3.70535000	-2.01761800
H	1.76528600	-3.38910600	-3.56242400
H	0.83826700	-3.30469800	-2.04263500
C	3.53671500	-1.32476400	-3.01673800
H	3.79816000	-0.25873100	-2.95252700
H	3.52730000	-1.60607500	-4.08115900
H	4.32262100	-1.91374900	-2.52526100
C	-2.26018800	3.26753300	-0.96394300
H	-2.83279600	3.72897500	-0.14434300
H	-2.21967700	3.98003300	-1.80269300
H	-1.23938900	3.07823300	-0.61368300
C	6.51035300	-2.31188000	0.04863500
H	7.49680800	-1.84388500	0.00825600
C	6.39516600	-3.70371300	0.12501100
H	7.29295700	-4.32622700	0.14910300
C	-7.79986500	-0.44795400	-1.18419400
H	-8.82331300	-0.59059400	-1.54015900
C	1.59232900	0.07421800	3.24902000
H	1.39349600	1.11855600	2.98461800
H	1.80567500	0.03571900	4.32784400
H	0.67926400	-0.50669900	3.05546300
C	4.04660600	0.33693200	2.77157700
H	4.92511300	-0.06480000	2.24704700
H	4.26185800	0.31767100	3.85164100

H	3.90332600	1.38401400	2.47452200
C	3.01355200	-1.94513500	2.95358900
H	2.18406200	-2.59938600	2.64574300
H	3.05725300	-1.95313300	4.05311900
H	3.95220300	-2.37575700	2.58471300
C	1.56910200	3.77447700	-1.89582600
C	2.01802500	2.49245000	-1.49670400
C	3.40395300	2.21380900	-1.61036400
C	4.28468100	3.15323500	-2.14511800
C	3.82157500	4.41489300	-2.53639200
C	2.46137700	4.71989700	-2.39622400
C	2.57030900	4.34365900	2.11513700
C	1.63464100	5.19196100	2.71338100
C	0.27213900	4.88668000	2.60540200
C	-0.15079200	3.76225600	1.89936900
C	0.78273200	2.88434100	1.30027800
C	2.15480400	3.20197000	1.42930000
N	1.12277100	1.56401400	-1.03120500
N	0.31349800	1.77031700	0.60136300
H	0.50793100	4.00410700	-1.79379000
H	3.77212000	1.24435200	-1.27089900
H	5.34586400	2.90599000	-2.24100900
H	4.51504000	5.15614700	-2.94094400
H	2.09148900	5.70618000	-2.68948600
H	3.63865400	4.56574800	2.18616700
H	1.96166200	6.08094800	3.25849300
H	-0.47260200	5.53829100	3.07081100
H	-1.21067200	3.52214500	1.82402800
H	2.89786300	2.54823900	0.97895100

3

C	3.78323400	0.82022900	0.22434700
N	4.93130300	0.27332700	0.30472500
Si	1.26261600	-0.11439800	-0.14713200
H	1.57104400	0.52323200	-1.44160100
C	-3.64087300	0.14094400	-0.28563700
N	2.66780900	0.21372700	0.87472900
Si	-1.60496800	-0.80805000	0.42676800
C	-0.52269900	1.82467000	0.46090400
N	-2.77548000	-0.61000400	-0.99665600
C	-1.15267700	2.47159400	-0.61886300
H	-1.47475900	1.86959100	-1.46973700
N	-3.10744800	0.35591900	0.90597900
C	-1.35960700	3.85193700	-0.60777000
H	-1.84878400	4.33204400	-1.45981600
N	-0.30927700	0.41376900	0.43766300
C	-0.93822200	4.62034200	0.48308300

H	-1.10158900	5.70066600	0.49374800
N	0.93775800	-1.83240400	-0.39588300
C	-0.29631800	3.99213200	1.55346400
H	0.04839900	4.58083900	2.40773400
N	-0.41009700	-2.15177300	-0.11437000
C	-0.08420600	2.61093600	1.53928200
H	0.43202900	2.12492400	2.36728900
C	-1.81137300	-2.26291800	1.62900900
C	-2.43434300	-2.84229000	2.73134900
H	-3.29214600	-2.37070900	3.21970500
C	-1.91667300	-4.04231600	3.25252700
H	-2.38337500	-4.50310900	4.12682100
C	-0.78508000	-4.63774000	2.67601100
H	-0.38437000	-5.55645700	3.11441500
C	-0.15635300	-4.08861300	1.54815800
H	0.72359600	-4.55058700	1.09676300
C	-0.71406300	-2.91815400	1.02653700
C	1.65912200	-2.66938700	-1.25470500
C	1.07045300	-3.81150600	-1.83438800
H	0.03163600	-4.04359200	-1.60060700
C	1.80973300	-4.62563800	-2.69487000
H	1.33077400	-5.50523600	-3.13474300
C	3.14332500	-4.33301900	-2.99740800
H	3.71616400	-4.97611800	-3.66952500
C	3.73430900	-3.20772500	-2.41115300
H	4.78076900	-2.96596200	-2.61832200
C	3.01318000	-2.38643500	-1.54541000
H	3.51853800	-1.54360000	-1.07062500
C	3.44592800	2.10640300	-0.48409200
C	3.61302600	2.24408800	-1.87102800
H	3.96276100	1.39103900	-2.45463100
C	3.29984700	3.44572100	-2.50732900
H	3.42126700	3.53491700	-3.58992300
C	2.82218900	4.53013600	-1.76222000
H	2.57603600	5.47178000	-2.25960600
C	2.64526900	4.39856400	-0.38224000
H	2.25370100	5.23305000	0.20341000
C	2.94410200	3.18912400	0.24971500
H	2.78498300	3.07563500	1.32285900
C	6.25326000	0.76126900	-0.09490600
C	6.58112100	0.18302500	-1.48601100
H	5.93970200	0.62356400	-2.26228900
H	6.43028700	-0.90780400	-1.49238400
H	7.62991300	0.39126300	-1.75176900
C	7.23219600	0.14674200	0.93107300
H	7.10776400	-0.94572400	0.96888700
H	7.03585000	0.54496000	1.93917000
H	8.27771100	0.37700300	0.67002400
C	6.45271000	2.29192800	-0.08562800

H	7.52644600	2.52200200	-0.17344900
H	6.08808200	2.72296400	0.85974300
H	5.93462000	2.79776200	-0.90889600
C	2.88989000	-0.51430900	2.18345600
C	1.53450400	-0.71369800	2.88546700
H	0.86226500	-1.37263100	2.32389800
H	1.01718000	0.23883700	3.06326000
H	1.70434600	-1.19810600	3.85845200
C	3.53567400	-1.89854300	1.97845800
H	3.67245800	-2.39525800	2.95295200
H	4.50761700	-1.79831500	1.48320600
H	2.88715600	-2.53552900	1.36072700
C	3.75933400	0.35783800	3.10772700
H	3.82120000	-0.10969900	4.10236700
H	3.31075100	1.35751900	3.22707100
H	4.77857300	0.47437700	2.72355100
C	-2.78090700	-1.13523900	-2.38359000
C	-3.77336800	-0.41213500	-3.30884000
H	-4.82110000	-0.59710600	-3.04322700
H	-3.62046800	-0.77958900	-4.33470500
H	-3.60443200	0.67474900	-3.31021600
C	-3.11958200	-2.63615400	-2.32114700
H	-2.39657200	-3.15914700	-1.67895800
H	-3.08466500	-3.08423800	-3.32686900
H	-4.13020000	-2.78619400	-1.90903300
C	-1.36684200	-0.94072000	-2.96701500
H	-1.09414500	0.12558700	-2.98783000
H	-1.33316500	-1.32226400	-3.99877000
H	-0.61485200	-1.48570500	-2.38740300
C	-3.67836900	0.97657400	2.12126100
C	-2.64369900	0.79673100	3.24647900
H	-1.69457600	1.27873200	2.98254700
H	-2.44446300	-0.26255900	3.45046800
H	-3.01663500	1.26167900	4.17149800
C	-3.92086300	2.48384400	1.91583200
H	-4.23198800	2.94220300	2.86780300
H	-4.71671400	2.66934800	1.18266000
H	-3.00399300	2.98331400	1.57541400
C	-4.99024200	0.28091600	2.53427000
H	-4.84468300	-0.80695800	2.61491600
H	-5.79705000	0.46955500	1.81326800
H	-5.32270900	0.65773600	3.51420900
C	-6.07139200	-0.24730300	-0.74312200
H	-5.95021200	-1.26681200	-0.36990600
C	-4.96937500	0.61898200	-0.75906800
C	-5.11590800	1.92644000	-1.24031500
H	-4.25191900	2.59428100	-1.25271800
C	-6.35829100	2.36261100	-1.70720900
H	-6.46765100	3.38127900	-2.08703000

C	-7.45812100	1.49887000	-1.68846500
H	-8.42956500	1.84233300	-2.05242200
C	-7.31374700	0.19444800	-1.20328600
H	-8.17118000	-0.48259800	-1.18619700

3´ (hypothetic isomer of **3** with a five-membered SiN_2C_2 ring)

C	3.78323400	0.82022900	0.22434700
N	4.93130300	0.27332700	0.30472500
Si	1.26261600	-0.11439800	-0.14713200
H	1.57104400	0.52323200	-1.44160100
C	-3.64087300	0.14094400	-0.28563700
N	2.66780900	0.21372700	0.87472900
Si	-1.60496800	-0.80805000	0.42676800
C	-0.52269900	1.82467000	0.46090400
N	-2.77548000	-0.61000400	-0.99665600
C	-1.15267700	2.47159400	-0.61886300
H	-1.47475900	1.86959100	-1.46973700
N	-3.10744800	0.35591900	0.90597900
C	-1.35960700	3.85193700	-0.60777000
H	-1.84878400	4.33204400	-1.45981600
N	-0.30927700	0.41376900	0.43766300
C	-0.93822200	4.62034200	0.48308300
H	-1.10158900	5.70066600	0.49374800
N	0.93775800	-1.83240400	-0.39588300
C	-0.29631800	3.99213200	1.55346400
H	0.04839900	4.58083900	2.40773400
N	-0.41009700	-2.15177300	-0.11437000
C	-0.08420600	2.61093600	1.53928200
H	0.43202900	2.12492400	2.36728900
C	-1.81137300	-2.26291800	1.62900900
C	-2.43434300	-2.84229000	2.73134900
H	-3.29214600	-2.37070900	3.21970500
C	-1.91667300	-4.04231600	3.25252700
H	-2.38337500	-4.50310900	4.12682100
C	-0.78508000	-4.63774000	2.67601100
H	-0.38437000	-5.55645700	3.11441500
C	-0.15635300	-4.08861300	1.54815800
H	0.72359600	-4.55058700	1.09676300
C	-0.71406300	-2.91815400	1.02653700
C	1.65912200	-2.66938700	-1.25470500
C	1.07045300	-3.81150600	-1.83438800
H	0.03163600	-4.04359200	-1.60060700
C	1.80973300	-4.62563800	-2.69487000
H	1.33077400	-5.50523600	-3.13474300
C	3.14332500	-4.33301900	-2.99740800
H	3.71616400	-4.97611800	-3.66952500
C	3.73430900	-3.20772500	-2.41115300
H	4.78076900	-2.96596200	-2.61832200

C	3.01318000	-2.38643500	-1.54541000
H	3.51853800	-1.54360000	-1.07062500
C	3.44592800	2.10640300	-0.48409200
C	3.61302600	2.24408800	-1.87102800
H	3.96276100	1.39103900	-2.45463100
C	3.29984700	3.44572100	-2.50732900
H	3.42126700	3.53491700	-3.58992300
C	2.82218900	4.53013600	-1.76222000
H	2.57603600	5.47178000	-2.25960600
C	2.64526900	4.39856400	-0.38224000
H	2.25370100	5.23305000	0.20341000
C	2.94410200	3.18912400	0.24971500
H	2.78498300	3.07563500	1.32285900
C	6.25326000	0.76126900	-0.09490600
C	6.58112100	0.18302500	-1.48601100
H	5.93970200	0.62356400	-2.26228900
H	6.43028700	-0.90780400	-1.49238400
H	7.62991300	0.39126300	-1.75176900
C	7.23219600	0.14674200	0.93107300
H	7.10776400	-0.94572400	0.96888700
H	7.03585000	0.54496000	1.93917000
H	8.27771100	0.37700300	0.67002400
C	6.45271000	2.29192800	-0.08562800
H	7.52644600	2.52200200	-0.17344900
H	6.08808200	2.72296400	0.85974300
H	5.93462000	2.79776200	-0.90889600
C	2.88989000	-0.51430900	2.18345600
C	1.53450400	-0.71369800	2.88546700
H	0.86226500	-1.37263100	2.32389800
H	1.01718000	0.23883700	3.06326000
H	1.70434600	-1.19810600	3.85845200
C	3.53567400	-1.89854300	1.97845800
H	3.67245800	-2.39525800	2.95295200
H	4.50761700	-1.79831500	1.48320600
H	2.88715600	-2.53552900	1.36072700
C	3.75933400	0.35783800	3.10772700
H	3.82120000	-0.10969900	4.10236700
H	3.31075100	1.35751900	3.22707100
H	4.77857300	0.47437700	2.72355100
C	-2.78090700	-1.13523900	-2.38359000
C	-3.77336800	-0.41213500	-3.30884000
H	-4.82110000	-0.59710600	-3.04322700
H	-3.62046800	-0.77958900	-4.33470500
H	-3.60443200	0.67474900	-3.31021600
C	-3.11958200	-2.63615400	-2.32114700
H	-2.39657200	-3.15914700	-1.67895800
H	-3.08466500	-3.08423800	-3.32686900
H	-4.13020000	-2.78619400	-1.90903300
C	-1.36684200	-0.94072000	-2.96701500

H	-1.09414500	0.12558700	-2.98783000
H	-1.33316500	-1.32226400	-3.99877000
H	-0.61485200	-1.48570500	-2.38740300
C	-3.67836900	0.97657400	2.12126100
C	-2.64369900	0.79673100	3.24647900
H	-1.69457600	1.27873200	2.98254700
H	-2.44446300	-0.26255900	3.45046800
H	-3.01663500	1.26167900	4.17149800
C	-3.92086300	2.48384400	1.91583200
H	-4.23198800	2.94220300	2.86780300
H	-4.71671400	2.66934800	1.18266000
H	-3.00399300	2.98331400	1.57541400
C	-4.99024200	0.28091600	2.53427000
H	-4.84468300	-0.80695800	2.61491600
H	-5.79705000	0.46955500	1.81326800
H	-5.32270900	0.65773600	3.51420900
C	-6.07139200	-0.24730300	-0.74312200
H	-5.95021200	-1.26681200	-0.36990600
C	-4.96937500	0.61898200	-0.75906800
C	-5.11590800	1.92644000	-1.24031500
H	-4.25191900	2.59428100	-1.25271800
C	-6.35829100	2.36261100	-1.70720900
H	-6.46765100	3.38127900	-2.08703000
C	-7.45812100	1.49887000	-1.68846500
H	-8.42956500	1.84233300	-2.05242200
C	-7.31374700	0.19444800	-1.20328600
H	-8.17118000	-0.48259800	-1.18619700

4

C	3.04618100	-0.83692300	0.35145300
N	2.24562600	-0.21550500	1.26297200
Si	1.01576400	0.35008500	-0.04210600
C	-3.77397300	-0.03871300	0.00346700
N	2.41431600	-0.87372700	-0.79728200
Si	-1.54248100	0.14834500	0.38183000
C	-0.41565900	-2.22835500	-0.56966600
N	-3.07428100	-0.61794800	1.00740000
C	0.34946100	-3.15904900	0.16060800
H	1.01758900	-2.78533600	0.93834100
N	-2.92781800	0.75140300	-0.65504700
C	0.24278100	-4.52657300	-0.09674000
H	0.84542800	-5.23192300	0.48207400
N	-0.35493200	-0.86138300	-0.27359100
C	-0.63144500	-4.99992400	-1.08244300
H	-0.71308000	-6.07120600	-1.28059400
N	-0.41497900	1.19334200	1.02934600
C	-1.39535500	-4.08467600	-1.81370600
H	-2.07546600	-4.43930700	-2.59307600

N	1.20458000	1.61304300	-1.10095100
C	-1.28611200	-2.71397700	-1.56462100
H	-1.86254700	-1.99448400	-2.15054900
C	-0.60490600	2.48631700	1.52663400
C	-1.72326500	2.76801600	2.34318900
H	-2.41533200	1.95613900	2.59124900
C	-1.94746100	4.04976300	2.84918900
H	-2.82362700	4.23509600	3.47755500
C	-1.05256700	5.08665100	2.56766500
H	-1.22140000	6.08984500	2.96659600
C	0.06344700	4.81593400	1.76771700
H	0.77097400	5.61504800	1.52959900
C	0.29005500	3.54057000	1.24709000
H	1.13691800	3.35280500	0.58993300
C	2.26319600	2.28350100	-1.59999500
C	3.45645200	2.54718900	-0.85251100
H	3.49749900	2.19972400	0.18242400
C	4.54151600	3.22923000	-1.40205800
H	5.43270900	3.39858400	-0.78739400
C	4.50561900	3.70124600	-2.71987600
H	5.35813900	4.23183300	-3.15060700
C	3.33626900	3.48854600	-3.46708400
H	3.27828000	3.85918000	-4.49611000
C	2.24587200	2.81115000	-2.92889800
H	1.34251900	2.65341200	-3.52481400
C	4.43805700	-1.31090900	0.60048000
C	5.49366900	-0.38744900	0.56670300
H	5.28263300	0.65837300	0.33622600
C	6.80241300	-0.81493400	0.80069500
H	7.62128500	-0.09218100	0.76667100
C	7.06487000	-2.16202100	1.07297400
H	8.08947100	-2.49388100	1.25889300
C	6.01394500	-3.08441900	1.10106100
H	6.21438800	-4.13820300	1.31008100
C	4.70451100	-2.66097800	0.86116400
H	3.88043400	-3.37727500	0.88674800
C	2.43212300	0.01608000	2.71802900
C	1.09268600	-0.27166600	3.42475200
H	0.29334500	0.36979700	3.04064300
H	1.19356200	-0.10034900	4.50778300
H	0.79978100	-1.32175000	3.26763200
C	2.84785700	1.48047900	2.94835100
H	3.82204500	1.68084000	2.47633900
H	2.93753200	1.69464300	4.02544000
H	2.11016100	2.17362800	2.52293000
C	3.48023300	-0.90848000	3.36589600
H	3.26700500	-1.96760100	3.15965500
H	3.44014200	-0.76215600	4.45614100
H	4.50493000	-0.69373500	3.04216300

C	2.86283200	-1.30227600	-2.14404800
C	4.14368900	-0.57422800	-2.59657900
H	4.02264100	0.51513300	-2.53796200
H	4.36291800	-0.84155700	-3.64238700
H	5.01324400	-0.86530700	-1.99214900
C	3.09199800	-2.82628600	-2.17692600
H	3.94768700	-3.12081300	-1.55437800
H	3.30809200	-3.14385800	-3.20938700
H	2.19925500	-3.36588300	-1.83053100
C	1.72594100	-0.95802000	-3.12478500
H	0.80420800	-1.49313200	-2.85707400
H	2.01422300	-1.26200300	-4.14283700
H	1.51709200	0.11862100	-3.11726000
C	-5.19833900	-0.28259900	-0.33201200
C	-6.21652000	0.44242100	0.30034000
H	-5.96385700	1.18108700	1.06379200
C	-7.54791200	0.22482500	-0.06115800
H	-8.34159900	0.79442100	0.42770700
C	-7.86338100	-0.71523000	-1.04772500
H	-8.90591300	-0.88264000	-1.32854900
C	-6.84532200	-1.44002200	-1.67631900
H	-7.08939900	-2.17475200	-2.44700400
C	-5.51157900	-1.22369600	-1.32325900
H	-4.71041100	-1.78350300	-1.81081700
C	-3.00882500	1.62345400	-1.85469400
C	-4.43081500	2.15048700	-2.08675500
H	-4.81640300	2.65487000	-1.18779000
H	-5.13702000	1.36006900	-2.37461300
H	-4.40161800	2.88780100	-2.90251900
C	-2.04778900	2.79991300	-1.60440300
H	-2.06850100	3.47765600	-2.47130700
H	-1.00640500	2.45832400	-1.46772600
H	-2.35025300	3.36718200	-0.71111800
C	-2.52286600	0.80203000	-3.06320200
H	-3.18042200	-0.06470800	-3.24069000
H	-1.49460300	0.44691000	-2.89722600
H	-2.52586500	1.42711400	-3.96915700
C	-3.42629800	-1.66636400	1.99302800
C	-2.15944300	-1.89193400	2.83339800
H	-1.84414700	-0.96432700	3.33400800
H	-1.32925200	-2.24954600	2.20607900
H	-2.35150500	-2.65157700	3.60511900
C	-4.56660000	-1.18427400	2.90488200
H	-4.74102100	-1.92109600	3.70386900
H	-5.50711700	-1.06395500	2.35020600
H	-4.30780700	-0.22229900	3.37435900
C	-3.80904700	-2.97559300	1.28087300
H	-3.00425800	-3.30055900	0.60471700
H	-4.73390000	-2.86063500	0.69792500

H	-3.97811300	-3.76859800	2.02560400

4´

Si	-1.15021900	0.26514800	-0.14184200
Si	1.15745400	-0.08121800	-0.13414100
N	0.24801000	1.12083400	-1.05743400
N	-2.62516500	0.77699400	-1.20293700
N	-2.84971000	-0.25877000	0.69821500
N	2.54858300	-0.96250800	-1.06294800
N	2.90289200	0.54450600	0.45190000
C	0.57243100	2.46462000	-1.09224000
C	-3.50667200	0.21480500	-0.34912100
C	3.49851200	-0.30945300	-0.38349900
C	4.95754100	-0.60672400	-0.48238100
C	-4.98244800	0.18398800	-0.55759500
C	-2.82094400	0.98311800	-2.67724600
C	2.63295100	-1.85012800	-2.26082300
C	3.55380200	1.53045500	1.37280300
C	1.76624100	2.89018800	-1.72540000
H	2.40150000	2.13150800	-2.18954900
C	-3.42096100	-0.85331700	1.94943900
C	5.43916400	-1.71796200	0.22387200
H	4.75414900	-2.30206700	0.84237500
C	5.83364000	0.13632800	-1.28482300
H	5.45820600	0.99265900	-1.84620400
C	-2.80689800	-0.39239700	-3.37275100
H	-1.86196100	-0.91361600	-3.15938500
H	-2.90123300	-0.27101100	-4.46333600
H	-3.63865800	-1.02611100	-3.03404600
C	-0.25260400	3.47060500	-0.53523400
H	-1.19004200	3.18073400	-0.05340800
C	-5.74564200	1.28269100	-0.14175000
H	-5.25274100	2.13559100	0.32933400
C	2.12971300	4.23482400	-1.76297700
H	3.06346400	4.52556500	-2.25370700
C	-1.67007200	1.82298400	-3.25004700
H	-1.61267400	2.80582900	-2.76340600
H	-1.85168000	1.98024800	-4.32383300
H	-0.70571300	1.32159400	-3.12000300
C	-5.61108300	-0.90425500	-1.17767500
H	-5.02118700	-1.77034200	-1.48031400
C	1.31038700	5.21428800	-1.18658200
H	1.59614100	6.26823600	-1.21668800
C	0.11296800	4.81713400	-0.58222800
H	-0.54438600	5.56581900	-0.13045400
C	-4.12903300	1.74041700	-2.98797800
H	-5.03217600	1.13854700	-2.84036400

H	-4.11266700	2.05081600	-4.04354300
H	-4.20786700	2.64693200	-2.36913100
C	7.17668100	-0.22842300	-1.37800100
H	7.85214400	0.35265500	-2.01028200
C	-3.90119400	0.27882700	2.87425500
H	-3.06678000	0.93072900	3.16093500
H	-4.33051100	-0.14622200	3.79512600
H	-4.68122400	0.88259700	2.38633600
C	-4.59418500	-1.81667500	1.66876400
H	-5.52647200	-1.30373000	1.40728500
H	-4.78624000	-2.39610100	2.58428100
H	-4.33299900	-2.52869400	0.87286500
C	-7.12761100	1.29295400	-0.34583200
H	-7.71711100	2.15300500	-0.01938500
C	-6.99066400	-0.88855400	-1.38617700
H	-7.47457700	-1.74020800	-1.87012900
C	2.50967600	2.55551500	1.83754400
H	2.09036200	3.10891300	0.98845800
H	3.00087900	3.27628600	2.50828200
H	1.69314800	2.09538100	2.39370700
C	4.66217200	2.33313400	0.65714900
H	5.58084100	1.75542900	0.50924300
H	4.92364900	3.20299800	1.27783600
H	4.30253800	2.71083600	-0.31113200
C	4.14683500	0.79618900	2.58828000
H	3.35927200	0.27783100	3.15095600
H	4.62639300	1.52006400	3.26591100
H	4.91001600	0.06581700	2.28007200
C	-2.33745300	-1.68696200	2.64799100
H	-2.08307200	-2.57534100	2.05811400
H	-2.72245100	-2.02129800	3.62318600
H	-1.42587900	-1.11768100	2.82974600
C	6.78709900	-2.07723500	0.13468300
H	7.15508200	-2.94234900	0.69134500
C	7.65729200	-1.33448400	-0.66779000
H	8.71015400	-1.61733100	-0.74206400
C	-7.75198200	0.20990200	-0.97165300
H	-8.83238100	0.22005500	-1.13490600
C	1.43785500	-1.51961100	-3.17671900
H	0.48731600	-1.63714100	-2.64435800
H	1.45610700	-2.18479500	-4.05373300
H	1.49848400	-0.47692800	-3.52437500
C	2.58423000	-3.32244700	-1.81958600
H	3.42188500	-3.54920400	-1.14080600
H	2.66822200	-3.98310800	-2.69697800
H	1.64552100	-3.55572000	-1.30304300
C	3.91170300	-1.62508600	-3.09038500
H	4.05370000	-0.56111100	-3.33101000
H	3.80219500	-2.17017000	-4.03998700

H	4.81741400	-2.00115300	-2.60005600
C	-0.68235600	1.65063800	3.21734000
C	0.05145300	0.59035200	2.62595700
C	0.82103800	-0.21997300	3.50083900
C	0.86065100	0.02495500	4.87241700
C	0.11902800	1.06965400	5.43756600
C	-0.65548000	1.87456600	4.59303800
C	-0.08285900	-4.48523100	1.16107000
C	-1.26097400	-5.10018700	0.72496600
C	-2.08795800	-4.40928100	-0.17093900
C	-1.74959600	-3.13229700	-0.61368500
C	-0.57235000	-2.48135500	-0.16559300
C	0.25659500	-3.20267900	0.72738600
N	0.02714900	0.36746800	1.27696400
N	-0.27109900	-1.20798400	-0.60869500
H	-1.24489900	2.31624400	2.55828600
H	1.36805900	-1.06382400	3.07399000
H	1.46777700	-0.62135600	5.51338000
H	0.14559200	1.25398600	6.51388000
H	-1.23428300	2.70381300	5.01077200
H	0.57775200	-5.00561900	1.86058400
H	-1.52744000	-6.10154700	1.07066200
H	-3.00811700	-4.87702600	-0.53387900
H	-2.39143200	-2.60492700	-1.32250700
H	1.17314200	-2.73426200	1.09545800

2

Si	-1.93621200	-0.20361200	0.78828200
O	-0.00005000	1.89183600	-0.00032600
N	3.73313800	-0.75494800	-1.19012800
C	-1.13785600	2.55639500	0.38069600
Si	1.93625100	-0.20363000	-0.78849500
N	2.92019100	-0.66843300	0.80221300
N	-2.92032400	-0.66841600	-0.80228600
C	-3.41471600	2.35722100	1.09726800
H	-4.28114500	1.74608800	1.36124100
N	-3.73303800	-0.75492500	1.19015200
C	-3.52278500	3.75279000	1.11301800
H	-4.46450600	4.23061600	1.39626400
C	-2.42340700	4.53537300	0.76436400
H	-2.51625100	5.62381400	0.78320400
C	-1.20048400	3.95621400	0.38475200
C	0.00000800	4.82877500	-0.00021200
C	1.20063900	3.95619600	-0.38470600
C	1.13792000	2.55638100	-0.38088900
C	2.21752000	1.72184900	-0.73969500
C	3.41496300	2.35716900	-1.09686700

H	4.28142800	1.74601900	-1.36068400
C	3.52311900	3.75273400	-1.11239300
H	4.46494200	4.23054400	-1.39532700
C	2.42369600	4.53533500	-0.76391700
H	2.51660000	5.62377200	-0.78261700
C	0.38334400	5.72236500	1.20604300
H	-0.45639800	6.36948700	1.50251000
H	1.23681400	6.37350100	0.96219000
H	0.66098900	5.10307700	2.07233700
C	-0.38351200	5.72151000	-1.20706100
H	-0.66103100	5.10159200	-2.07294600
H	0.45612700	6.36857800	-1.50395000
H	-1.23713500	6.37260400	-0.96361700
C	-3.95273200	-1.11333500	-0.08846900
C	3.95268200	-1.11334700	0.08851400
C	-4.36354700	-1.22442300	2.43724200
C	-4.15714200	-2.74089300	2.62219800
H	-4.67621100	-3.31178100	1.83871400
H	-4.55132700	-3.06973300	3.59735900
H	-3.08432200	-2.98530500	2.57947600
C	-5.86556700	-0.88538200	2.47434200
H	-6.02687500	0.18518600	2.27325900
H	-6.27583400	-1.11252900	3.47102400
H	-6.43576500	-1.46714300	1.73852100
C	-3.66199400	-0.47546400	3.58500900
H	-2.58192900	-0.69202900	3.59077400
H	-4.07846700	-0.78636400	4.55524600
H	-3.79176400	0.61227400	3.48251700
C	-2.61688300	-0.68257200	-2.24385500
C	-1.77890400	-1.93393700	-2.57100000
H	-0.85646200	-1.94224100	-1.97015900
H	-1.49775100	-1.94064200	-3.63664200
H	-2.35111100	-2.85123000	-2.36215800
C	-3.88430200	-0.64549600	-3.11607000
H	-4.45581000	-1.58156900	-3.07321700
H	-3.59250700	-0.48053300	-4.16473800
H	-4.54599700	0.17967100	-2.81086000
C	-1.78393700	0.57989300	-2.53318800
H	-2.35927800	1.48815100	-2.29848900
H	-1.49492700	0.60743100	-3.59524900
H	-0.86310500	0.59058500	-1.93334900
C	2.61661000	-0.68245500	2.24375000
C	1.77857700	-1.93377900	2.57091200
H	0.85622500	-1.94214800	1.96993500
H	1.49726900	-1.94035700	3.63651400
H	2.35081700	-2.85109500	2.36226000
C	3.88391500	-0.64527600	3.11613700
H	4.45541600	-1.58136200	3.07351800
H	3.59196500	-0.48014900	4.16473600

H	4.54567000	0.17983400	2.81090500
C	1.78363200	0.58005100	2.53281400
H	2.35902800	1.48828100	2.29813800
H	1.49441800	0.60769400	3.59481700
H	0.86291500	0.59069900	1.93279700
C	4.36376200	-1.22458100	-2.43710000
C	3.66239100	-0.47570900	-3.58504100
H	2.58230500	-0.69217000	-3.59088500
H	4.07892300	-0.78677500	-4.55519900
H	3.79225200	0.61203000	-3.48268000
C	4.15730400	-2.74106500	-2.62186600
H	4.67616100	-3.31184700	-1.83815900
H	4.55169200	-3.07008900	-3.59688200
H	3.08446100	-2.98540800	-2.57934400
C	5.86580900	-0.88564200	-2.47402400
H	6.02715300	0.18494200	-2.27305200
H	6.27619800	-1.11293700	-3.47062300
H	6.43587400	-1.46734200	-1.73804800
C	5.08037300	-1.94476300	0.60085500
C	4.87963100	-3.30485200	0.87950900
H	3.90226600	-3.75358400	0.68918300
C	5.92243900	-4.07901200	1.39482500
H	5.75900700	-5.13863100	1.60652600
C	7.17050500	-3.49806900	1.64239000
H	7.98446500	-4.10243400	2.05054900
C	7.37457500	-2.14168100	1.36724900
H	8.34724700	-1.68331200	1.56225900
C	6.33486300	-1.36850200	0.84600200
H	6.48686100	-0.30764100	0.63588800
C	-5.08045700	-1.94474600	-0.60075000
C	-4.87967700	-3.30479400	-0.87959000
H	-3.90226900	-3.75349800	-0.68942300
C	-5.92250100	-4.07894500	-1.39488700
H	-5.75904000	-5.13853200	-1.60672600
C	-7.17062000	-3.49803300	-1.64225800
H	-7.98459300	-4.10239100	-2.05040000
C	-7.37471700	-2.14168100	-1.36696700
H	-8.34742400	-1.68333000	-1.56184500
C	-6.33498600	-1.36850900	-0.84574700
H	-6.48700500	-0.30767100	-0.63553000
C	-2.21741000	1.72187400	0.73967600

TS1 (xanthene-based)

Si	-2.33335400	-0.16724900	0.25526400
Si	1.96458200	-0.63276000	-0.04969500
O	-0.16941400	1.18048700	-1.27099100
N	-4.15283700	-0.59079100	0.66562300

N	-2.98678800	-1.61160900	-0.83612000
N	3.68546200	-0.57288500	0.82306300
N	3.28559400	-1.71198900	-0.95354800
N	-1.94066300	2.64076200	1.67462400
N	-1.83019000	1.49690100	2.20062900
C	-4.11865300	-1.67147600	-0.13722200
C	4.19447800	-1.50237300	0.00323600
C	-0.85453800	3.51921400	1.47348500
C	-1.14841400	4.89547600	1.46137900
H	-2.17309400	5.20759100	1.67419300
C	-0.14703800	5.82435100	1.18095600
H	-0.37967900	6.89242600	1.19190400
C	1.14424400	5.39075200	0.85371600
H	1.92060100	6.11677200	0.60052100
C	1.42639500	4.02003000	0.81653500
H	2.41349900	3.67037100	0.50550100
C	0.44383200	3.08808300	1.14403200
H	0.65759300	2.02254600	1.09900000
C	-0.80912400	1.12259800	3.11806500
C	-0.43356400	2.01785800	4.14383100
H	-0.80945200	3.04184700	4.12588600
C	0.38151600	1.58931400	5.19018700
H	0.65861700	2.29669500	5.97645500
C	0.83279100	0.26530200	5.24531200
H	1.47327500	-0.06699600	6.06590800
C	0.45735000	-0.62452900	4.23359900
H	0.80552400	-1.66024700	4.25879900
C	-0.34487800	-0.20574400	3.17212400
H	-0.60161100	-0.87851500	2.35415200
C	-1.34465800	1.81443500	-1.60251900
C	-2.50388100	1.27011400	-1.01620300
C	-3.73080300	1.83800500	-1.37918600
H	-4.65001300	1.43078100	-0.95457100
C	-3.79691800	2.91353400	-2.26977400
H	-4.76291500	3.34581500	-2.54382400
C	-2.62179300	3.44206400	-2.80102700
H	-2.68106300	4.29001600	-3.48717400
C	-1.36451300	2.90506400	-2.48043200
C	-0.08278400	3.49101500	-3.07848200
C	-0.01447700	5.00177200	-2.74587800
H	-0.88177000	5.53647900	-3.16099800
H	0.88867200	5.46425500	-3.17212300
H	-0.00139300	5.15660400	-1.65804500
C	-0.10286000	3.29333100	-4.61556700
H	-0.15524100	2.22309400	-4.86760200
H	0.80498600	3.71077000	-5.07881100
H	-0.97436000	3.79392800	-5.06541700
C	1.15113200	2.78223300	-2.50797700
C	2.44121500	3.22423300	-2.83296700

H	2.55883000	4.09257300	-3.48492100
C	3.57979000	2.58396000	-2.34260300
H	4.57352500	2.95523400	-2.60687200
C	3.43550700	1.46402900	-1.52085600
H	4.33012000	0.96190100	-1.14230700
C	2.17282900	0.96723300	-1.16438900
C	1.05057200	1.65961700	-1.66776300
C	-5.08557500	-2.80620300	-0.16433200
C	-6.23615900	-2.75355900	-0.96212400
H	-6.43490300	-1.86062800	-1.55898900
C	-7.11628200	-3.83663400	-0.99579500
H	-8.01123700	-3.79036300	-1.62124100
C	-6.85346500	-4.97913700	-0.23177800
H	-7.54369800	-5.82605300	-0.25840400
C	-5.70567500	-5.03563500	0.56562000
H	-5.49734500	-5.92552500	1.16487000
C	-4.82207700	-3.95342800	0.59748300
H	-3.92508300	-3.99121000	1.22008600
C	-5.01670600	-0.26337700	1.82389100
C	-5.18776800	1.26775300	1.85618600
H	-5.69094800	1.56682500	2.78938100
H	-5.81336800	1.60279300	1.01405700
H	-4.22724200	1.79408800	1.79906000
C	-4.31918800	-0.74768700	3.11085700
H	-3.34671200	-0.25176400	3.22959300
H	-4.15917100	-1.83732400	3.07213200
H	-4.93728600	-0.52178100	3.99485700
C	-6.41944100	-0.89044300	1.74084400
H	-7.03679000	-0.46841000	2.54882200
H	-6.40944200	-1.98069500	1.86528700
H	-6.90788000	-0.65488600	0.78391400
C	-2.37642800	-2.48361400	-1.84962200
C	-1.46276100	-3.50804900	-1.14664500
H	-2.05326600	-4.16612300	-0.49032100
H	-0.70730100	-2.99054700	-0.53503800
H	-0.94424500	-4.13881600	-1.88688200
C	-3.41294800	-3.21337400	-2.72151000
H	-2.89443800	-3.70377200	-3.56006900
H	-4.14362000	-2.50363800	-3.13837300
H	-3.95939600	-3.98767300	-2.16805800
C	-1.53086200	-1.56817600	-2.75475000
H	-1.00942600	-2.16211000	-3.52059200
H	-0.77455300	-1.02435600	-2.17178100
H	-2.16915400	-0.82467200	-3.25494400
C	5.48045900	-2.24164300	0.17873200
C	6.67315800	-1.73835200	-0.36058500
H	6.65907500	-0.79051400	-0.90291200
C	7.87161300	-2.43792800	-0.19713600
H	8.79578000	-2.03747000	-0.62094500

C	7.88821900	-3.64495700	0.50894500
H	8.82588000	-4.19130600	0.63774500
C	6.70180600	-4.15143600	1.04982400
H	6.70942500	-5.09475700	1.60150100
C	5.50262600	-3.45418600	0.88411100
H	4.57287500	-3.85179800	1.29674900
C	4.15239700	-0.06060500	2.12332700
C	5.59935300	0.45913400	2.03313800
H	6.32272100	-0.35217300	1.87811200
H	5.86709500	0.97189600	2.97025900
H	5.69864700	1.18047700	1.20697700
C	4.04379500	-1.14693700	3.21000300
H	3.01835700	-1.54154800	3.24680700
H	4.28672400	-0.72869300	4.19986600
H	4.73674700	-1.97753700	3.01166200
C	3.22913400	1.11653500	2.47678300
H	3.30030800	1.90448700	1.71395700
H	3.50600900	1.54771100	3.44988400
H	2.18129400	0.79279800	2.54924600
C	3.33979500	-2.48522300	-2.20267800
C	4.42278900	-1.90708900	-3.13443400
H	4.40017300	-2.41671200	-4.11098400
H	5.42713800	-2.04204100	-2.70846200
H	4.25104100	-0.83207500	-3.29576300
C	1.96489900	-2.32488900	-2.87141400
H	1.74270000	-1.26458700	-3.06632300
H	1.16950200	-2.72987400	-2.23012800
H	1.94054200	-2.86259600	-3.83144400
C	3.59785200	-3.98198900	-1.94377000
H	2.85934900	-4.37957900	-1.23008200
H	4.60338300	-4.15945200	-1.53888000
H	3.50925800	-4.54863000	-2.88449500

Int1 (xanthene-based)

Si	-2.34862900	0.23898000	0.27503000
Si	2.00938400	-0.11515100	-0.62358600
O	-0.08621200	2.11968600	-0.56982300
N	-3.91977000	-0.71629800	0.39702200
N	-2.43622500	-1.11318400	-1.13649900
N	3.49869400	-0.09908500	0.58204200
N	3.58702600	-0.89177300	-1.41290700
N	-2.03689000	0.77730000	1.98523200
N	-1.06456200	-0.17916400	1.38160600
C	-3.57613000	-1.54251400	-0.61908700
C	4.22850300	-0.86389200	-0.22980100
C	-1.53498200	1.98193900	2.47171900
C	-2.36998100	3.12266700	2.46377200

H	-3.35936200	3.05206900	2.00792200
C	-1.93251400	4.32770900	3.01070300
H	-2.59630100	5.19680500	2.98729600
C	-0.65229600	4.43970800	3.56760600
H	-0.30977200	5.38776700	3.98883800
C	0.18491300	3.31920100	3.56108800
H	1.19253300	3.38890200	3.98011200
C	-0.24451200	2.10437800	3.02343100
H	0.41300600	1.23694200	3.02009600
C	-0.70047400	-1.30426100	2.11377800
C	-0.77255200	-1.31947100	3.52244800
H	-1.17807200	-0.44867800	4.03826200
C	-0.33107800	-2.43349400	4.23970800
H	-0.39206000	-2.42070600	5.33177400
C	0.18460600	-3.55583700	3.58300700
H	0.53006400	-4.42298700	4.15093700
C	0.24558100	-3.54800900	2.18331500
H	0.64175800	-4.41637400	1.64797300
C	-0.18865700	-2.44206300	1.45319600
H	-0.12054300	-2.43021200	0.36910800
C	-1.30730900	2.64358400	-0.91085500
C	-2.43293400	1.83212600	-0.69851100
C	-3.67913800	2.32433400	-1.11706500
H	-4.57636300	1.71744600	-0.96495500
C	-3.78911300	3.57849700	-1.72281100
H	-4.76292400	3.94428100	-2.05760700
C	-2.65426200	4.38043900	-1.87192400
H	-2.76037300	5.37419500	-2.30885700
C	-1.39452400	3.93710300	-1.44940000
C	-0.13204800	4.80047400	-1.41591500
C	0.03804000	5.31962100	0.03964500
H	-0.81831900	5.95056600	0.32505400
H	0.96299400	5.91218100	0.12523700
H	0.09094200	4.49018900	0.75883700
C	-0.22012800	5.99919200	-2.36898300
H	-0.33925400	5.67949300	-3.41567100
H	0.68255400	6.62257600	-2.29616200
H	-1.06762200	6.64742000	-2.10278600
C	1.05874100	3.89081600	-1.73018200
C	2.20897000	4.30700800	-2.41105800
H	2.25848000	5.31278100	-2.83042500
C	3.31060800	3.45648100	-2.54522400
H	4.20313300	3.80332700	-3.07317900
C	3.27042600	2.17399100	-1.99534700
H	4.13862500	1.51624000	-2.09325300
C	2.13086700	1.69340500	-1.32578800
C	1.04550800	2.58039500	-1.22071800
C	-4.29820800	-2.78029600	-1.02140600
C	-3.79183400	-4.00757000	-0.57067500

H	-2.90276200	-4.02293900	0.06455700
C	-4.43237800	-5.19837000	-0.92646000
H	-4.03935300	-6.15252100	-0.56751700
C	-5.56891200	-5.16686800	-1.73954200
H	-6.06649100	-6.09828800	-2.02067500
C	-6.06984900	-3.94095400	-2.19414200
H	-6.95676400	-3.91410700	-2.83169900
C	-5.44009500	-2.74997600	-1.83328900
H	-5.82871200	-1.79246100	-2.18450500
C	-4.91640100	-0.90315100	1.49099900
C	-5.18501800	0.47980200	2.11021400
H	-5.95077900	0.38843000	2.89521000
H	-5.55592100	1.18371700	1.34830100
H	-4.27306500	0.88834900	2.55862000
C	-4.33704300	-1.85179900	2.55429100
H	-3.40527700	-1.44512800	2.96891600
H	-4.12298000	-2.84094100	2.12023100
H	-5.05718000	-1.98868100	3.37670300
C	-6.25603000	-1.45595100	0.97140900
H	-7.00329100	-1.37703700	1.77536500
H	-6.19808800	-2.51181100	0.68218400
H	-6.62098800	-0.87363200	0.11141600
C	-1.79258200	-1.43987500	-2.43537400
C	-0.79787100	-2.59996200	-2.25294900
H	-1.29895300	-3.48817000	-1.84085500
H	0.02027200	-2.30011100	-1.58307300
H	-0.35687300	-2.87397100	-3.22412600
C	-2.81428600	-1.80366400	-3.52924200
H	-2.30349000	-1.80576100	-4.50424600
H	-3.62534100	-1.06031400	-3.57422200
H	-3.25479000	-2.79791900	-3.38561800
C	-1.03803500	-0.18062200	-2.89288900
H	-0.47071300	-0.39882900	-3.80990900
H	-0.32736900	0.15025700	-2.12477400
H	-1.73316800	0.64464500	-3.10199700
C	5.40910900	-1.67957300	0.17453100
C	5.20623000	-2.82523300	0.95983100
H	4.18889300	-3.11047700	1.23833700
C	6.29667200	-3.58900300	1.38293100
H	6.13130900	-4.48113600	1.99210500
C	7.59673200	-3.20868900	1.03279700
H	8.45018500	-3.80274900	1.36914400
C	7.80281400	-2.06520200	0.25377400
H	8.81731000	-1.76251100	-0.01721100
C	6.71299500	-1.30447000	-0.17634000
H	6.86947200	-0.40695500	-0.77851100
C	3.70850000	0.41561700	1.94469200
C	5.19361000	0.47434000	2.34089600
H	5.63227800	-0.52111200	2.48625100

H	5.28635900	1.02265100	3.29107700
H	5.78476400	1.00648900	1.57989700
C	2.93136500	-0.46536400	2.94072300
H	1.87332200	-0.54508900	2.65571300
H	2.98668100	-0.04538700	3.95754200
H	3.34607000	-1.48456700	2.96623700
C	3.14635400	1.84933300	1.94967800
H	3.71448400	2.48915700	1.25787000
H	3.20261300	2.28097800	2.96069000
H	2.09466300	1.86934100	1.63382200
C	3.74985400	-1.78647900	-2.57093000
C	5.17210100	-1.70183000	-3.15458000
H	5.21711800	-2.24843600	-4.10981300
H	5.91864800	-2.14391800	-2.48229000
H	5.44892300	-0.65312100	-3.34502400
C	2.75333200	-1.28786600	-3.63273900
H	2.96760400	-0.24447100	-3.90874500
H	1.72300600	-1.33289700	-3.25035500
H	2.80927000	-1.91202600	-4.53771000
C	3.41389100	-3.24384400	-2.19699300
H	2.39798400	-3.30369100	-1.77701700
H	4.12107800	-3.63424800	-1.45055000
H	3.46324300	-3.89451400	-3.08518400

Ts2 (xanthene-based)

Si	-1.89028500	0.17550300	0.80503900
Si	1.63737800	0.03305900	-0.90436900
O	-0.01759200	2.33186900	-0.24215000
N	-3.46255100	-0.79492400	0.95349900
N	-2.58943200	-0.66634900	-1.03917000
N	3.13522400	-0.00841300	0.25548700
N	3.11907500	-0.89659700	-1.71535500
N	-1.36560200	0.15974300	2.46913700
N	-0.21331800	-0.42511700	0.83443700
C	-3.58302300	-1.16470900	-0.34975100
C	3.81509000	-0.81451700	-0.57270800
C	-0.67710200	0.97626400	3.31046100
C	-0.61470100	2.39432300	3.18468900
H	-1.14153800	2.87377800	2.36051700
C	0.09683400	3.16694100	4.09860800
H	0.12358400	4.25396400	3.97722700
C	0.78563200	2.56645800	5.16146300
H	1.34954600	3.17859900	5.86996700
C	0.73429100	1.17339000	5.31245200
H	1.25931200	0.69606400	6.14506700
C	0.00891500	0.39423100	4.41581000
H	-0.04915100	-0.69012100	4.52961500

C	0.10167300	-1.76070100	1.14537600
C	-0.53096600	-2.87697000	0.55542200
H	-1.32365500	-2.70574300	-0.16958000
C	-0.12995300	-4.17913500	0.85938900
H	-0.62901500	-5.02350200	0.37495700
C	0.90007500	-4.41014700	1.77718700
H	1.20860200	-5.43035700	2.01878700
C	1.52587700	-3.31456600	2.38685700
H	2.32333100	-3.47620800	3.11822200
C	1.14000700	-2.01357700	2.06911300
H	1.62213200	-1.16028600	2.54056500
C	-1.31142900	2.79326000	-0.27754500
C	-2.30078700	1.91585500	0.18008000
C	-3.62543400	2.37841800	0.11868000
H	-4.43500700	1.72842900	0.46103900
C	-3.92555100	3.64382000	-0.39470200
H	-4.96400900	3.98141100	-0.44362600
C	-2.90238800	4.48606400	-0.84396200
H	-3.15607700	5.47260400	-1.23470600
C	-1.56387600	4.07761000	-0.78224700
C	-0.35172600	4.94629500	-1.14787900
C	0.24089800	5.51059200	0.17406700
H	-0.49818500	6.15095000	0.68158500
H	1.14389900	6.10628400	-0.03451800
H	0.51710100	4.69637200	0.86091900
C	-0.72845400	6.11292200	-2.06881000
H	-1.15535700	5.75621700	-3.01855000
H	0.15245900	6.73162800	-2.29432200
H	-1.46164300	6.77389300	-1.58386700
C	0.69972400	4.02580100	-1.78113700
C	1.58462900	4.37430400	-2.81024800
H	1.52165300	5.35909200	-3.27600400
C	2.56501200	3.47300400	-3.24521000
H	3.25173700	3.76900400	-4.04284200
C	2.67080500	2.20304700	-2.66775300
H	3.43948700	1.50822300	-3.01656300
C	1.79001300	1.80013400	-1.64875500
C	0.83622800	2.74129100	-1.24093400
C	-4.66565700	-2.03257600	-0.89921200
C	-4.41949200	-3.40049200	-1.07537500
H	-3.45465100	-3.81713000	-0.77794700
C	-5.40928600	-4.22830700	-1.61289200
H	-5.21299300	-5.29534900	-1.74322800
C	-6.64625200	-3.69130200	-1.98215200
H	-7.41956000	-4.33742700	-2.40496900
C	-6.89237200	-2.32419800	-1.80908300
H	-7.85747600	-1.90101500	-2.09829500
C	-5.90726200	-1.49815800	-1.26759200
H	-6.09393300	-0.43036400	-1.13472700

C	-4.17256900	-1.28621800	2.17385800
C	-4.32928100	-0.09721700	3.14243300
H	-4.84027100	-0.42940800	4.05942300
H	-4.93486500	0.69883100	2.68088600
H	-3.35601900	0.31887500	3.42487400
C	-3.33316000	-2.40528300	2.81988300
H	-2.32922600	-2.03278600	3.06114900
H	-3.23327000	-3.25625300	2.12746700
H	-3.81718900	-2.76588600	3.74207800
C	-5.58987300	-1.82182800	1.90016300
H	-6.06193700	-2.03771500	2.87063400
H	-5.59789400	-2.75128400	1.31886100
H	-6.21401800	-1.07997900	1.38189800
C	-2.33619000	-0.67720200	-2.49434700
C	-1.49670300	-1.92261800	-2.83829500
H	-2.05839500	-2.84423400	-2.62278600
H	-0.57106400	-1.92889900	-2.24676000
H	-1.23060000	-1.92709600	-3.90726300
C	-3.60423900	-0.65126300	-3.37066900
H	-3.30974500	-0.44463600	-4.41154300
H	-4.28720200	0.14861300	-3.04706200
H	-4.15087800	-1.60214200	-3.36509900
C	-1.52627800	0.58939100	-2.81264800
H	-1.19918700	0.58298400	-3.86363000
H	-0.63343700	0.63737300	-2.17951200
H	-2.12376300	1.49510700	-2.63707400
C	5.03870300	-1.58351900	-0.21315300
C	4.91268900	-2.70459400	0.62194000
H	3.92560900	-2.98454000	0.99693000
C	6.04410000	-3.44365700	0.97381900
H	5.94028100	-4.31850600	1.62052900
C	7.30644500	-3.06144900	0.50654400
H	8.19173600	-3.63723000	0.78800000
C	7.43471000	-1.94049300	-0.32037500
H	8.41930600	-1.63849100	-0.68578000
C	6.30336200	-1.20585000	-0.68443700
H	6.39640700	-0.33735600	-1.33993900
C	3.66185500	0.85222600	1.34205200
C	4.68288700	1.84029800	0.74325800
H	5.56866000	1.31278500	0.35749300
H	5.02172500	2.55106000	1.51374500
H	4.22664300	2.40887200	-0.08084300
C	4.32642300	0.03786000	2.46878900
H	3.64966200	-0.73965800	2.84941100
H	4.57550300	0.71069500	3.30379500
H	5.25508400	-0.44650200	2.13992500
C	2.47587400	1.63572200	1.91656200
H	2.04034100	2.30986400	1.16870700
H	2.79602800	2.24244800	2.77510400

H	1.67709000	0.96516400	2.24949000
C	3.11199400	-1.95576900	-2.74566400
C	4.51693900	-2.22077600	-3.31481600
H	4.43532300	-2.87050500	-4.20029200
H	5.17118000	-2.72352400	-2.59136000
H	4.99691900	-1.27897300	-3.62311700
C	2.21353100	-1.43069900	-3.87910600
H	2.63774400	-0.51662700	-4.32133600
H	1.20805300	-1.19105600	-3.50416700
H	2.10989600	-2.18805800	-4.67103800
C	2.52189900	-3.25516000	-2.16298400
H	1.52182300	-3.07599200	-1.73998000
H	3.16127600	-3.64817700	-1.35851600
H	2.44108700	-4.02879800	-2.94352700

Int2 (xanthene-based)

Si	-1.12654400	-0.05919100	0.94957200
Si	1.60421600	0.31659300	-0.73785700
O	-0.00376600	2.41397900	-0.03575100
N	-2.40954900	-1.37483400	0.83698700
N	-2.48340400	-0.67955800	-1.32309700
N	3.08252800	0.38817300	0.37944000
N	2.92319900	-0.71849200	-1.47362700
N	-0.31599900	0.04605000	2.38492800
N	0.25814200	-0.58060700	-0.20807100
C	-3.07929000	-1.32792300	-0.38244700
C	3.73906200	-0.43860700	-0.43571500
C	-0.38119000	0.71636400	3.56608200
C	-0.87757700	2.04796800	3.71780600
H	-1.28225900	2.55758600	2.84152100
C	-0.85150000	2.70967700	4.94481100
H	-1.24149700	3.73152900	5.00578100
C	-0.33623600	2.09339500	6.09268300
H	-0.31799000	2.61778900	7.05148100
C	0.14985300	0.78431800	5.97835100
H	0.55218800	0.27472300	6.86052700
C	0.12841800	0.11426900	4.75627400
H	0.51156100	-0.90738100	4.68402800
C	0.49002400	-2.00204500	-0.09558700
C	-0.21219400	-2.90144800	-0.91469300
H	-0.92846900	-2.49404800	-1.62490600
C	-0.01231700	-4.27887500	-0.79492500
H	-0.57348200	-4.96125100	-1.43913700
C	0.89816400	-4.78652800	0.13866700
H	1.05367800	-5.86428900	0.22971700
C	1.59467900	-3.89835300	0.96363000

H	2.28805000	-4.27967600	1.71845200
C	1.38371400	-2.52154000	0.85628500
H	1.87354600	-1.83350000	1.53941500
C	-1.37584300	2.61049400	0.03006500
C	-2.09805600	1.52509700	0.50043300
C	-3.47938500	1.73937100	0.63084400
H	-4.10648000	0.94807400	1.04215000
C	-4.06235700	2.93966300	0.21273800
H	-5.14202100	3.07978900	0.31052500
C	-3.28788000	3.95731800	-0.36440200
H	-3.77601700	4.86261200	-0.72868400
C	-1.89899300	3.80844800	-0.46522800
C	-0.90836600	4.81752400	-1.07643600
C	-0.21290200	5.58413200	0.08118500
H	-0.95809500	6.15630700	0.65538600
H	0.54099400	6.28263000	-0.31606000
H	0.28574500	4.88806100	0.77159000
C	-1.60343500	5.81618600	-2.00830800
H	-2.10952600	5.30519100	-2.84148300
H	-0.87871200	6.53194900	-2.42484800
H	-2.35118600	6.40526400	-1.45775400
C	0.16002400	3.99529200	-1.81747800
C	0.79430900	4.30282300	-3.02637300
H	0.53121500	5.21421200	-3.56633400
C	1.77570900	3.45157500	-3.56250200
H	2.26324500	3.72338500	-4.50172500
C	2.11960200	2.25372500	-2.92755600
H	2.86421500	1.59609400	-3.37877100
C	1.47912000	1.89496000	-1.72902300
C	0.56311400	2.80309000	-1.20993500
C	-4.43676800	-1.94966200	-0.56805100
C	-4.57065000	-3.11474500	-1.33650800
H	-3.67400100	-3.60789700	-1.71746700
C	-5.83002800	-3.66159700	-1.59003500
H	-5.91656400	-4.57611100	-2.18258500
C	-6.97800000	-3.04168600	-1.08541700
H	-7.96532100	-3.46528600	-1.28610700
C	-6.85387200	-1.87718400	-0.32049800
H	-7.74534500	-1.38496800	0.07668000
C	-5.59208600	-1.33804900	-0.06139700
H	-5.50548300	-0.42725400	0.53124200
C	-2.75804100	-2.23466900	2.03430200
C	-3.45796900	-1.35180500	3.08531600
H	-3.70703300	-1.94044900	3.98291300
H	-4.39631800	-0.94172100	2.67957000
H	-2.81429900	-0.51744900	3.39804000
C	-1.46260300	-2.83427100	2.62064600
H	-0.72141300	-2.05502000	2.83065400
H	-1.03030900	-3.55869900	1.91618800

H	-1.69901300	-3.36401400	3.55692300
C	-3.65433300	-3.45181000	1.72663400
H	-3.64817600	-4.09696500	2.61840700
H	-3.25619200	-4.04125700	0.88870200
H	-4.69903000	-3.20154500	1.51660500
C	-2.92392600	-0.27622300	-2.65958700
C	-2.56403800	-1.37875500	-3.67866000
H	-3.11919600	-2.30552200	-3.47814700
H	-1.48610400	-1.60168400	-3.64248000
H	-2.80750000	-1.05158400	-4.70266300
C	-4.41572800	0.09777000	-2.80345100
H	-4.57419600	0.60874200	-3.76709700
H	-4.71321800	0.78714600	-1.99920600
H	-5.08013400	-0.77495500	-2.77666700
C	-2.09030600	0.97841300	-3.00184500
H	-2.24284900	1.28340500	-4.04995600
H	-1.02066000	0.77884500	-2.84317700
H	-2.37462600	1.81850500	-2.35377000
C	5.10010200	-0.99281000	-0.21521200
C	5.23940100	-2.15303700	0.55928600
H	4.35016200	-2.64610600	0.95628900
C	6.51225100	-2.67537400	0.80224500
H	6.61923400	-3.57992600	1.40531900
C	7.64301900	-2.04290600	0.27621400
H	8.63757700	-2.45176700	0.47006700
C	7.50163100	-0.88601300	-0.49793500
H	8.38383200	-0.38872300	-0.90771000
C	6.23240700	-0.35949500	-0.74497500
H	6.11818100	0.55032400	-1.33765400
C	3.43460300	1.01778300	1.68474700
C	4.88819400	1.51602100	1.69138900
H	5.61574000	0.69426100	1.71564600
H	5.04706600	2.12653100	2.59277700
H	5.09551500	2.14589400	0.81178100
C	3.19396400	0.01048100	2.82064800
H	2.13639800	-0.29235600	2.83150700
H	3.41610900	0.48117300	3.78965700
H	3.84180400	-0.87297300	2.70996400
C	2.48142100	2.20892500	1.86048000
H	2.62033000	2.95011800	1.05776800
H	2.67490400	2.69799400	2.82576800
H	1.43718400	1.86997400	1.86956200
C	3.10830100	-1.56829700	-2.67680500
C	4.30601800	-1.08285200	-3.51351100
H	4.34557200	-1.64351200	-4.45988200
H	5.25838300	-1.24363900	-2.99145300
H	4.21738500	-0.01310800	-3.75657600
C	1.82035400	-1.42975100	-3.50666600
H	1.65818500	-0.39214800	-3.83369800

H	0.94374800	-1.75359200	-2.92996200
H	1.88634300	-2.06113600	-4.40471000
C	3.30754000	-3.04107100	-2.28075700
H	2.46837500	-3.40178500	-1.67383500
H	4.23745000	-3.18064700	-1.71266500
H	3.37230800	-3.66093500	-3.18840800

Ts3 (xanthene-based)

Si	-1.24282000	0.44305400	0.72490400
Si	1.55099200	0.40187200	-0.21676600
O	0.02002300	2.76680100	-0.07631900
N	-2.55263700	-0.78792400	1.05848900
N	-3.10926500	-0.50188900	-1.13851300
N	2.95258900	-0.24164200	0.81717800
N	2.70827300	-0.70709000	-1.27886600
N	-0.06360100	0.74715700	1.91030200
N	-0.03017500	-0.32136000	-0.43215400
C	-3.25079100	-1.21499000	-0.08169600
C	3.47054300	-0.93170800	-0.21424000
C	-0.07037800	1.57442800	3.02776700
C	-0.16782000	2.99188300	2.96360900
H	-0.28030600	3.46329100	1.99142200
C	-0.10595400	3.79168600	4.10583400
H	-0.18444800	4.87836900	3.99932900
C	0.05350500	3.22639500	5.37514400
H	0.10253700	3.85606500	6.26718000
C	0.14039400	1.83382700	5.47442700
H	0.25283300	1.36143000	6.45543600
C	0.07864400	1.03249300	4.33419500
H	0.13147800	-0.05043600	4.44404000
C	-0.04258300	-1.75578100	-0.52473000
C	-0.57978000	-2.40916900	-1.64228400
H	-1.01753400	-1.80629900	-2.42998900
C	-0.55322100	-3.80203500	-1.75182500
H	-0.98164500	-4.27911300	-2.63672900
C	0.02130700	-4.58064300	-0.74388900
H	0.04955800	-5.66923200	-0.83255300
C	0.53640100	-3.94615000	0.39215200
H	0.96047000	-4.53878900	1.20769800
C	0.49256500	-2.55381900	0.50670700
H	0.86517300	-2.06536600	1.40551500
C	-1.28198100	2.99156400	-0.42126200
C	-2.14602500	2.00809300	0.04335100
C	-3.50584800	2.27881100	-0.17267600
H	-4.26194900	1.57139100	0.17178300
C	-3.89768000	3.42111900	-0.88308400
H	-4.96115600	3.61086900	-1.05095500

C	-2.95410900	4.30892300	-1.43033200
H	-3.30078000	5.15897500	-2.02173000
C	-1.58689700	4.10896900	-1.20084900
C	-0.41640300	5.01690300	-1.64652000
C	-0.14663100	6.03788200	-0.50835900
H	-1.03950200	6.65843000	-0.33311700
H	0.69816400	6.69513600	-0.77010400
H	0.09939700	5.52000500	0.43091200
C	-0.75024000	5.76566900	-2.94408600
H	-0.94526000	5.06539800	-3.77045800
H	0.07434900	6.43243700	-3.23805000
H	-1.63867500	6.40018200	-2.81073500
C	0.85447600	4.15253100	-1.80995300
C	1.94969100	4.38621200	-2.64953400
H	1.95398400	5.24301500	-3.32656200
C	3.06772600	3.53231500	-2.62058100
H	3.92507800	3.75593900	-3.26028800
C	3.08654400	2.38207200	-1.82188500
H	3.94442200	1.71017700	-1.86593900
C	1.98405400	2.07665200	-1.00571300
C	0.97361500	3.03290800	-0.98192700
C	-3.96673700	-2.53651000	-0.02652600
C	-3.22123800	-3.68957600	0.25476900
H	-2.16189700	-3.59190900	0.48205500
C	-3.82952300	-4.94650700	0.25803800
H	-3.22900300	-5.83587600	0.46662500
C	-5.19987800	-5.06458200	0.00480700
H	-5.67998200	-6.04656800	0.01576400
C	-5.95556700	-3.91533400	-0.25356500
H	-7.03015100	-3.99585700	-0.43716400
C	-5.34027900	-2.66230800	-0.27551300
H	-5.93684000	-1.76874800	-0.46405600
C	-3.00702400	-0.99142400	2.48596300
C	-3.21004500	0.40289100	3.12246000
H	-3.45884100	0.30460500	4.19039900
H	-4.03844400	0.92709600	2.62142600
H	-2.32296200	1.04044400	3.06723600
C	-1.93171800	-1.80211400	3.22982300
H	-0.95092200	-1.32588200	3.11510400
H	-1.87361700	-2.81862700	2.81153900
H	-2.16769000	-1.87897300	4.30339000
C	-4.36182600	-1.70544300	2.66586600
H	-4.63454400	-1.62273000	3.72929400
H	-4.33864000	-2.77110000	2.41691500
H	-5.16071800	-1.22610100	2.08250700
C	-3.61602300	-0.60888800	-2.50554200
C	-3.76614600	-2.02666200	-3.10664100
H	-4.61259100	-2.57580400	-2.67740200
H	-2.86818600	-2.63570200	-2.94690800

H	-3.93637600	-1.94544200	-4.19228200
C	-4.98573200	0.10548700	-2.58346900
H	-5.35009700	0.11458400	-3.62361600
H	-4.90039600	1.14449100	-2.23855200
H	-5.73878900	-0.40533300	-1.96726700
C	-2.62796000	0.20988600	-3.36987600
H	-2.98378200	0.29106900	-4.40961100
H	-1.62933100	-0.24959400	-3.38606800
H	-2.51423300	1.21948900	-2.94854100
C	4.62831300	-1.86429500	-0.13750500
C	4.38554200	-3.16806600	0.31870200
H	3.36663200	-3.46612600	0.57394700
C	5.44214300	-4.07495500	0.42765300
H	5.24920400	-5.09085500	0.78036300
C	6.74095700	-3.68384100	0.08650500
H	7.56727400	-4.39330000	0.17536000
C	6.98183900	-2.38310600	-0.36839700
H	7.99575500	-2.07323400	-0.63217200
C	5.92819300	-1.47386000	-0.48191500
H	6.11740500	-0.45383800	-0.82172800
C	3.44100900	0.00490500	2.20129000
C	4.93777300	-0.29227500	2.38718200
H	5.17949500	-1.35962000	2.31165000
H	5.22029700	0.04136200	3.39702700
H	5.55680200	0.26146500	1.66613300
C	2.62026500	-0.87710300	3.15279200
H	1.55247300	-0.70248800	2.97384300
H	2.84938400	-0.63827200	4.20189800
H	2.85206900	-1.94030500	2.97683300
C	3.22709000	1.50350400	2.47870300
H	3.87762100	2.10661100	1.82572500
H	3.46857500	1.73391900	3.52670000
H	2.18801800	1.80117000	2.30816500
C	2.84325100	-1.19053300	-2.67886200
C	4.18444300	-0.76683100	-3.31284200
H	4.18924500	-1.07000900	-4.37120700
H	5.04034300	-1.25379600	-2.82956200
H	4.32416400	0.32201300	-3.28025600
C	1.70928600	-0.53199600	-3.48299100
H	1.83193600	0.56028300	-3.50995100
H	0.72963000	-0.74916000	-3.04623000
H	1.72087300	-0.90560700	-4.51757200
C	2.73685900	-2.72513800	-2.74169800
H	1.83982900	-3.08368200	-2.22798600
H	3.61442200	-3.20366500	-2.28560500
H	2.68719000	-3.05063400	-3.79252100

5

C	3.78938800	-0.97106400	-0.55344400

N	3.04455300	-1.17358200	0.64081700
O	-0.19212100	2.14054300	0.60521300
Si	1.70677900	-0.03468500	0.92244900
C	-3.71703200	-0.71347300	-0.38500700
N	3.13971900	-0.58694100	-1.58403800
Si	-2.40278400	0.38922500	1.50657200
H	-2.74904700	0.63031200	2.92992200
C	0.99787000	2.60900700	0.14038700
N	-3.75302900	-0.69812100	1.00261700
C	2.06541700	1.68796500	0.20484300
N	-2.65262800	-0.15566200	-0.85645800
C	3.33270000	2.17749300	-0.13367800
H	4.20104200	1.52694700	-0.07798600
N	0.06260900	-0.73196200	0.65092900
C	3.51589000	3.49089600	-0.57323900
H	4.51533400	3.84437700	-0.83723800
N	-0.80617500	-0.41248600	1.74935700
C	2.41747200	4.34030000	-0.69689000
H	2.56772900	5.35654400	-1.06807200
C	1.12779400	3.92096100	-0.33886600
C	-0.07703300	4.86399100	-0.46730200
C	-0.20950600	5.30509800	-1.94655600
H	-0.37550600	4.43185600	-2.59538400
H	0.70051400	5.82023100	-2.28946200
H	-1.05313000	5.99888700	-2.08042400
C	0.14152200	6.10433800	0.43363400
H	-0.70828900	6.79998400	0.35599900
H	1.05159200	6.65089500	0.14169400
H	0.24394200	5.80442200	1.48736700
C	-1.36641000	4.15460100	-0.03836600
C	-2.62126600	4.77770200	-0.14326000
H	-2.68129500	5.79760700	-0.52966900
C	-3.79498000	4.12577600	0.23703700
H	-4.75593400	4.63807000	0.14652700
C	-3.73586300	2.81703500	0.72988400
H	-4.65902300	2.30525100	1.01915900
C	-2.51054400	2.15147100	0.83943600
C	-1.35260000	2.85078100	0.46841000
C	5.28876300	-1.10200000	-0.45671300
C	6.00682000	-1.99976900	-1.26034800
H	5.46793700	-2.65769500	-1.94327700
C	7.40033900	-2.06453400	-1.19308800
H	7.94072600	-2.77693700	-1.82144200
C	8.10130300	-1.21768600	-0.32880000
H	9.19228200	-1.26091000	-0.28137100
C	7.39617200	-0.32326400	0.48274700
H	7.93339200	0.33437900	1.17073200
C	6.00172600	-0.27901500	0.42947400
H	5.45446800	0.38693100	1.09815500

C	3.27025000	-2.45989500	1.43551200
C	2.05284700	-2.81700500	2.31339600
H	1.12783200	-2.89642400	1.73183100
H	1.90142700	-2.11117200	3.13615000
H	2.24026200	-3.80433300	2.76123200
C	3.45076600	-3.63528500	0.45458800
H	3.53116300	-4.57635200	1.01990800
H	4.36066300	-3.54669500	-0.14930400
H	2.58340000	-3.70845500	-0.21697600
C	4.48404300	-2.34095800	2.37827400
H	4.39065200	-1.44062400	3.00443200
H	5.43843900	-2.29691500	1.84160800
H	4.51923900	-3.21609100	3.04622100
C	3.57850300	-0.11956300	-2.90664900
C	3.56178200	-1.33513300	-3.85704200
H	4.34685000	-2.05984600	-3.59851200
H	3.72915200	-1.00789800	-4.89547100
H	2.58919300	-1.84886100	-3.80910400
C	4.94244300	0.59904900	-2.98793200
H	5.00258500	1.40910600	-2.24824900
H	5.04872700	1.05055200	-3.98680000
H	5.79588900	-0.07099300	-2.83490900
C	2.48656000	0.86805300	-3.37024200
H	1.50574000	0.37609000	-3.36328700
H	2.69374800	1.22656000	-4.39104200
H	2.43393400	1.73640900	-2.69850600
C	-0.24077800	-1.91459300	-0.04005300
C	-1.22415900	-2.82198700	0.40666400
H	-1.78121800	-2.59633400	1.31070500
C	-1.49384200	-3.99224000	-0.30449400
H	-2.26542300	-4.67163800	0.07036800
C	-0.78822700	-4.30916700	-1.46974700
H	-1.00105000	-5.22778600	-2.02136600
C	0.20178300	-3.42129200	-1.90605600
H	0.77497000	-3.64127800	-2.81165800
C	0.46870300	-2.24003000	-1.21493600
H	1.24792900	-1.56581300	-1.57386500
C	-0.05760600	-0.09985400	2.91609000
C	-0.57557600	-0.10686400	4.22508400
H	-1.60483700	-0.40355300	4.41358600
C	0.24999600	0.23945700	5.29603300
H	-0.16333000	0.22181000	6.30830900
C	1.59239700	0.59149300	5.09833600
H	2.22241100	0.86547600	5.94800300
C	2.11401000	0.56570800	3.80340700
H	3.16671400	0.81481700	3.63543900
C	1.30518200	0.21162200	2.71817500
C	-4.85415800	-1.26566700	-1.18718400
C	-6.04661800	-0.53976900	-1.31265600

H	-6.13150100	0.44052000	-0.83878500
C	-7.11342500	-1.05761300	-2.05231500
H	-8.03761400	-0.48235700	-2.14919100
C	-6.99685000	-2.30854000	-2.66663500
H	-7.83264200	-2.71706700	-3.24030200
C	-5.80517100	-3.03283800	-2.54899400
H	-5.70803400	-4.00936600	-3.02993700
C	-4.73588800	-2.51257400	-1.81812800
H	-3.80597300	-3.07528600	-1.71617500
C	-4.68488700	-1.42902300	1.92535100
C	-5.21479100	-2.75125500	1.33320600
H	-4.39421700	-3.35052900	0.91246100
H	-5.97205800	-2.60436100	0.55664900
H	-5.68456700	-3.33387400	2.13987900
C	-5.86643100	-0.51035600	2.28862100
H	-6.54146400	-1.00495600	3.00583900
H	-6.45137400	-0.25321200	1.39412400
H	-5.50253800	0.42229300	2.74757900
C	-3.93654200	-1.81288800	3.22268000
H	-3.64685000	-0.93489100	3.80954200
H	-3.03078200	-2.39860900	3.00686300
H	-4.59676300	-2.42767400	3.85235200
C	-2.37963000	0.25686800	-2.25362600
C	-3.42686600	1.27982600	-2.74229400
H	-4.42013300	0.82627100	-2.86141100
H	-3.12376300	1.68236800	-3.72239000
H	-3.50481000	2.11869800	-2.03438500
C	-2.31377800	-0.93897000	-3.22670400
H	-1.68046400	-1.73696100	-2.81716200
H	-1.87774200	-0.61005200	-4.18331500
H	-3.30653800	-1.35025000	-3.44457000
C	-1.00827700	0.95150800	-2.25507800
H	-1.05619700	1.90529700	-1.72060400
H	-0.69534300	1.16651900	-3.28735600
H	-0.24698300	0.32894000	-1.77390100

5´ (hypothetic isomer of **5** with a four-membered $SiNC_2$ ring)

C	3.80483600	-0.89540600	-0.14621000
N	3.16806600	-1.04559800	1.09259900
O	-0.06582900	2.23867500	0.17279700
Si	1.75731800	0.04937900	1.30862300
C	-3.66841800	-0.80542200	-0.70716200
N	3.11209200	-0.32589300	-1.06551700
Si	-2.41843100	0.69850900	0.70620700
H	1.68989800	0.25280400	2.77556400
C	1.21091400	2.70783900	0.21426100
N	-4.01505700	-0.24357300	0.47845400
C	2.15848500	1.79884000	0.71012000

N	-2.48582300	-0.35873000	-1.06234500
C	3.47524100	2.25872300	0.84562200
H	4.23888800	1.59125400	1.25227400
N	0.20100700	-0.76341000	0.89812100
C	3.83181300	3.55214300	0.45578500
H	4.86449400	3.89337300	0.56008900
N	-0.97010800	-0.17687700	1.38393700
C	2.86711100	4.40323800	-0.08673600
H	3.16426300	5.40055700	-0.41787700
C	1.52899800	4.00343400	-0.21901400
C	0.48220100	4.91235500	-0.88188000
C	0.84234800	5.05311300	-2.38339500
H	0.84590700	4.06765500	-2.87345500
H	1.84199200	5.49846600	-2.50660700
H	0.11369900	5.69467700	-2.90337700
C	0.49018700	6.30470000	-0.20864600
H	-0.24162400	6.97697000	-0.68074700
H	1.47631800	6.78450600	-0.29671800
H	0.24139400	6.22102000	0.85986000
C	-0.91834700	4.29672200	-0.76712800
C	-2.07019400	4.96856900	-1.21274100
H	-1.97456300	5.96918800	-1.64049000
C	-3.33520700	4.38500400	-1.12797400
H	-4.21025500	4.93318000	-1.48591700
C	-3.48205700	3.10285300	-0.58129300
H	-4.47689500	2.65387800	-0.50827500
C	-2.36581800	2.39476700	-0.12660600
C	-1.11055200	3.01570100	-0.24396500
C	5.22645000	-1.34445600	-0.32064100
C	5.51144000	-2.44781400	-1.13857600
H	4.68782400	-3.00204800	-1.59322100
C	6.83018600	-2.84874600	-1.35371400
H	7.03918300	-3.71656300	-1.98428200
C	7.88338300	-2.13873200	-0.76552000
H	8.91734100	-2.44843400	-0.93687800
C	7.60732500	-1.02880800	0.03878700
H	8.42536800	-0.46327900	0.49200200
C	6.28466400	-0.63699100	0.26303300
H	6.07384300	0.23888400	0.87913600
C	3.58571600	-1.98352500	2.20755300
C	2.32386200	-2.54886900	2.89360900
H	1.74194600	-3.17066100	2.20067700
H	1.67208900	-1.76203700	3.28506200
H	2.62592900	-3.17365300	3.74728200
C	4.36675300	-3.22102900	1.72079100
H	4.37233600	-3.96314300	2.53337300
H	5.41063700	-3.01038800	1.46886700
H	3.87354200	-3.68032600	0.85192000
C	4.42728900	-1.21323700	3.24149000

H	3.86321500	-0.36265300	3.65299800
H	5.35237500	-0.83116000	2.78797400
H	4.70915000	-1.87261100	4.07812500
C	3.50669300	0.16338900	-2.39659500
C	3.00886900	-0.87170300	-3.42564400
H	3.54902100	-1.82411200	-3.31125600
H	3.17126100	-0.50792000	-4.45297900
H	1.93590100	-1.06804200	-3.28996100
C	5.00311600	0.46118700	-2.64156000
H	5.40916800	1.09910200	-1.84315100
H	5.09916400	1.01010500	-3.59174600
H	5.62585200	-0.43760500	-2.71557700
C	2.75914700	1.49887200	-2.60335100
H	1.70323800	1.40181800	-2.33830800
H	2.83517600	1.82479200	-3.65306400
H	3.19056700	2.28427000	-1.96824700
C	0.05860400	-2.10767800	0.48815100
C	-0.96067400	-2.91821100	1.02983100
H	-1.64142900	-2.49240000	1.76673700
C	-1.08851200	-4.25207300	0.63991300
H	-1.87765400	-4.86349200	1.08753000
C	-0.21693800	-4.81406000	-0.30095100
H	-0.32199000	-5.85857800	-0.60343000
C	0.78398100	-4.00758000	-0.85154300
H	1.46108000	-4.41540300	-1.60768200
C	0.91906000	-2.66992200	-0.47405700
H	1.66787900	-2.03861800	-0.94852500
C	-1.23276100	0.18160100	2.72241600
C	-0.68650200	-0.18706000	3.95259400
H	0.11968400	-0.91367100	4.03403400
C	-1.22000900	0.42336500	5.10321400
H	-0.80054800	0.16555700	6.08010200
C	-2.25964200	1.35094000	5.02187800
H	-2.63772100	1.82970300	5.92878800
C	-2.83221100	1.65656200	3.76809800
H	-3.66409200	2.36733800	3.72592200
C	-2.34077700	1.05412400	2.61958100
C	-4.47328300	-1.81537400	-1.45485700
C	-5.50800000	-1.44747700	-2.32337800
H	-5.75384100	-0.39195300	-2.45698100
C	-6.23168700	-2.42847000	-3.00575800
H	-7.03695100	-2.13516100	-3.68372000
C	-5.93020400	-3.78117900	-2.81760300
H	-6.49919500	-4.54756300	-3.34985000
C	-4.89651100	-4.15015000	-1.94997500
H	-4.65117200	-5.20501100	-1.80454300
C	-4.16578500	-3.17172300	-1.27344700
H	-3.34173100	-3.45093700	-0.61454900
C	-5.12223500	-0.56256300	1.42372400

C	-4.57431000	-1.43447300	2.57190600
H	-3.77147700	-0.92783800	3.12127600
H	-4.17892600	-2.37935700	2.16572100
H	-5.37744600	-1.67847600	3.28530900
C	-6.31133900	-1.30598900	0.78778700
H	-7.10385500	-1.37952100	1.54807200
H	-6.06367700	-2.32536100	0.46908200
H	-6.72345600	-0.76209200	-0.07258300
C	-5.67158600	0.78011100	1.94132200
H	-6.14505700	1.33591600	1.11637900
H	-4.88446300	1.40571700	2.36925000
H	-6.42957000	0.60797100	2.72113600
C	-1.88004800	-0.39736000	-2.41603400
C	-2.74515700	0.41566400	-3.40172400
H	-3.71431500	-0.06673300	-3.58599800
H	-2.22862000	0.50365200	-4.37093300
H	-2.92141400	1.42940800	-3.01027700
C	-1.68176200	-1.83527600	-2.93648400
H	-1.18569600	-2.45805000	-2.17990900
H	-1.04073700	-1.81072100	-3.83189100
H	-2.62691500	-2.31253000	-3.22277600
C	-0.51086000	0.27998400	-2.32379700
H	-0.61659900	1.33881400	-2.06304500
H	-0.01066000	0.22635700	-3.30057200
H	0.13021000	-0.19477300	-1.57291500

8

Si	1.46128100	0.15346200	0.22919300
Si	-1.21836400	-0.03815800	0.29741600
O	-0.04326000	1.87254100	-1.46583200
N	2.93704300	-0.67825300	-0.98861500
N	2.87493300	-0.61476900	1.19640900
N	-3.18070400	-0.97571100	-1.20325000
N	-2.51174400	-1.11078400	0.97232000
N	0.14973800	-0.85230900	-0.48106700
N	0.05960400	0.67086600	1.32779900
C	3.62977700	-0.93473300	0.08954400
C	-3.53120700	-1.25992700	0.00338200
C	0.22121900	-2.12755200	-1.08141500
C	-0.41216400	-2.41126100	-2.30273500
H	-0.95800800	-1.61807600	-2.79591100
C	-0.34574400	-3.68349100	-2.87216300
H	-0.84371000	-3.86891900	-3.82113600
C	0.35915400	-4.71081600	-2.24175100
H	0.40947400	-5.70053100	-2.68746600
C	0.99851000	-4.44520800	-1.02894600
H	1.54633500	-5.23371200	-0.51746000
C	0.92824700	-3.17454500	-0.45668000

H	1.41835000	-2.97934400	0.49266000
C	-0.12326700	1.67291000	2.28778800
C	0.88372900	2.59441600	2.65073400
H	1.83270100	2.57914900	2.13100000
C	0.67321400	3.54956500	3.64409300
H	1.47763300	4.24089500	3.88602400
C	-0.54589500	3.63421600	4.31728500
H	-0.70479000	4.38071800	5.09041800
C	-1.55579500	2.73461900	3.97290300
H	-2.51750900	2.77392600	4.47987800
C	-1.34924700	1.77288600	2.98724000
H	-2.14996900	1.07911400	2.74961700
C	1.05448800	2.61152100	-1.06983900
C	2.02842700	1.89884800	-0.37856700
C	3.18697000	2.62968400	-0.06714200
H	4.01005400	2.15278900	0.46030800
C	3.27523800	3.99344600	-0.36879200
H	4.17613800	4.54356100	-0.10844300
C	2.20142500	4.67795100	-0.95373900
H	2.27806000	5.74927100	-1.11361600
C	1.04212100	3.98502200	-1.31547800
C	-0.25885300	4.56904400	-1.90954400
C	-0.27351400	4.31985800	-3.44320000
H	0.56837400	4.83502500	-3.92047600
H	-1.20447400	4.69952700	-3.88014200
H	-0.19777700	3.25335600	-3.67172000
C	-0.36788600	6.07899800	-1.64789600
H	-0.36125300	6.30546700	-0.57655600
H	-1.29030100	6.48091700	-2.07995500
H	0.46353100	6.61373100	-2.11908400
C	-1.42718700	3.79348600	-1.25889400
C	-2.64554900	4.31667600	-0.81676500
H	-2.87861400	5.36826000	-0.95385100
C	-3.56891100	3.48846600	-0.16763500
H	-4.51199500	3.90833700	0.17278000
C	-3.27552800	2.14774000	0.09935800
H	-3.98801700	1.56081600	0.66895800
C	-2.06423000	1.57371400	-0.31767400
C	-1.23233800	2.42871200	-1.03589700
C	5.01561000	-1.50685000	0.12369500
C	5.18940700	-2.88996300	-0.02599400
H	4.31884300	-3.52669800	-0.15095900
C	6.46706100	-3.44861400	-0.00030300
H	6.58630200	-4.52283200	-0.11116100
C	7.58705900	-2.63180900	0.16623400
H	8.58227700	-3.06716100	0.18608700
C	7.42282900	-1.25208400	0.30016000
H	8.28961400	-0.60770700	0.41804600
C	6.14479800	-0.69195700	0.27853100

H	6.02686000	0.38374200	0.36273100
C	3.35009700	-0.70164000	-2.42159700
C	2.22529000	-0.03030000	-3.23279400
H	2.45279900	-0.11579900	-4.30115400
H	2.14042500	1.03070300	-2.98895200
H	1.26140900	-0.50555800	-3.04724500
C	3.52343800	-2.14686300	-2.93190100
H	2.61498100	-2.73241600	-2.77226600
H	4.35897700	-2.65287200	-2.44191800
H	3.73371000	-2.12682200	-4.00784600
C	4.64561500	0.10168200	-2.66684800
H	4.80489500	0.20507000	-3.74650800
H	5.52798500	-0.38789500	-2.24959300
H	4.56299300	1.10685100	-2.24049500
C	3.31185600	-0.71026600	2.63231600
C	4.06951500	-2.02254900	2.94028700
H	5.08815200	-2.04068900	2.55403900
H	3.52963300	-2.88981000	2.54497900
H	4.13092000	-2.13669800	4.02815200
C	4.19678100	0.49579200	3.01129800
H	4.49656100	0.42288100	4.06339000
H	3.65213800	1.43551600	2.88440300
H	5.10907100	0.53307400	2.40989800
C	2.07580900	-0.71123000	3.54231200
H	2.41049300	-0.71888600	4.58537300
H	1.47462000	-1.60688500	3.37714300
H	1.44826600	0.16575400	3.40080600
C	-4.92593900	-1.62955000	0.44700700
C	-5.51106300	-2.84040800	0.05010000
H	-4.93220500	-3.54699900	-0.53544800
C	-6.82456300	-3.15203800	0.40544600
H	-7.25535600	-4.09965900	0.09342200
C	-7.58111700	-2.24989000	1.15415000
H	-8.60647900	-2.48714300	1.42437800
C	-7.00811900	-1.04261200	1.55999600
H	-7.58598600	-0.33520400	2.14878500
C	-5.69031200	-0.74207900	1.21916300
H	-5.24801100	0.18715200	1.56207900
C	-4.02929500	-0.77992100	-2.40610900
C	-5.41353400	-0.13528700	-2.17095600
H	-6.11743900	-0.79754600	-1.66502900
H	-5.84924100	0.12996500	-3.14166300
H	-5.31716300	0.78606300	-1.58752500
C	-4.19357300	-2.13400700	-3.12973400
H	-3.22607000	-2.63200900	-3.24374300
H	-4.62282000	-1.97746500	-4.12654100
H	-4.86082800	-2.80399900	-2.58095200
C	-3.23879700	0.17275000	-3.33110300
H	-3.12614700	1.15733300	-2.87075200

H	-3.76614700	0.29511500	-4.28431000
H	-2.23840700	-0.21519200	-3.54207800
C	-2.36918500	-2.11636700	2.09975600
C	-2.42683400	-3.55579400	1.54726200
H	-2.22765300	-4.26788600	2.35702600
H	-1.67166900	-3.70328900	0.76921000
H	-3.40722000	-3.80182100	1.13503700
C	-3.42822800	-1.92681000	3.20824500
H	-4.43190200	-2.21500500	2.89712400
H	-3.45397400	-0.88531300	3.54614600
H	-3.15798400	-2.55022000	4.06881000
C	-0.99969100	-1.93711800	2.76412300
H	-0.89287100	-0.95660800	3.23091600
H	-0.19060000	-2.07016200	2.04492300
H	-0.88755400	-2.69787800	3.54445000

C. References

[1] Y. Xiong, S. Dong, S. Yao, C. Dai, J. Zhu, S. Kemper, M. Driess, *Angew. Chem. Int. Ed.* **2022**, *61*, e202209250.

[2] Y. Wang, A. Kostenko, S. Yao, M. Driess, *J. Am. Chem. Soc.* **2017**, *139*, 13499–13506.

[3] G. M. Sheldrick, *Acta Crystallogr. Sect. C Struct. Chem.* **2015**, *71*, 3–8.

[4] L. J. Bourhis, O. V. Dolomanov, R. J. Gildea, J. A. K. Howard, H. Puschmann, *Acta Crystallogr. Sect. Found. Adv.* **2015**, *71*, 59–75.

[5] O. V. Dolomanov, L. J. Bourhis, R. J. Gildea, J. A. K. Howard, H. Puschmann, *J. Appl. Crystallogr.* **2009**, *42*, 339–341.

[6] A. H. Cook, *J. Chem. Soc. Resumed* **1938**, 876.

[7] M. J. Frisch, G. W. Trucks, H. B. Schlegel, G. E. Scuseria, M. A. Robb, J. R. Cheeseman, G. Scalmani, V. Barone, G. A. Petersson, H. Nakatsuji, X. Li, M. Caricato, A. V. Marenich, J. Bloino, B. G. Janesko, R. Gomperts, B. Mennucci, H. P. Hratchian, J. V. Ortiz, A. F. Izmaylov, J. L. Sonnenberg, Williams, F. Ding, F. Lipparini, F. Egidi, J. Goings, B. Peng, A. Petrone, T. Henderson, D. Ranasinghe, V. G. Zakrzewski, J. Gao, N. Rega, G. Zheng, W. Liang, M. Hada, M. Ehara, K. Toyota, R. Fukuda, J. Hasegawa, M. Ishida, T. Nakajima, Y. Honda, O. Kitao, H. Nakai, T. Vreven, K. Throssell, J. A. Montgomery Jr., J. E. Peralta, F. Ogliaro, M. J. Bearpark, J. J. Heyd, E. N. Brothers, K. N. Kudin, V. N. Staroverov, T. A. Keith, R. Kobayashi, J. Normand, K. Raghavachari, A. P. Rendell, J. C. Burant, S. S. Iyengar, J. Tomasi, M. Cossi, J. M. Millam, M. Klene, C. Adamo, R. Cammi, J. W. Ochterski, R. L. Martin, K. Morokuma, O. Farkas, J. B. Foresman, D. J. Fox, *Gaussian 16 Rev C01* **2016**.

[8] C. Lee, W. Yang, R. G. Parr, *Phys. Rev. B* **1988**, *37*, 785–789.

[9] K. Raghavachari, *Theor. Chem. Acc. Theory Comput. Model. Theor. Chim. Acta* **2000**, *103*, 361–363.

[10] J. Tomasi, B. Mennucci, R. Cammi, *Chem. Rev.* **2005**, *105*, 2999–3094.

[11] S. Grimme, J. Antony, S. Ehrlich, H. Krieg, *J. Chem. Phys.* **2010**, *132*, 154104.

[12] A. Bondi, *J. Phys. Chem.* **1964**, *68*, 441–451.

[13] G. Knizia, *J. Chem. Theory Comput.* **2013**, *9*, 4834–4843.

[14] G. Knizia, J. E. M. N. Klein, *Angew. Chem. Int. Ed.* **2015**, *54*, 5518–5522.

[15] F. Neese, *WIREs Comput. Mol. Sci.* **2012**, *2*, 73–78.

[16] M. Mitoraj, A. Michalak, *J. Mol. Model.* **2008**, *14*, 681–687.

[17] A. Michalak, M. Mitoraj, T. Ziegler, *J. Phys. Chem. A* **2008**, *112*, 1933–1939.
